# Feasibility, Enjoyment, and Language Comprehension Impact of a Tablet- and GameFlow-Based Story-Listening Game for Kindergarteners: Methodological and Mixed Methods Study

**DOI:** 10.2196/34698

**Published:** 2022-03-23

**Authors:** Femke Vanden Bempt, Maria Economou, Ward Dehairs, Maaike Vandermosten, Jan Wouters, Pol Ghesquière, Jolijn Vanderauwera

**Affiliations:** 1 Parenting and Special Education Research Unit Faculty of Psychology and Educational Sciences KU Leuven Leuven Belgium; 2 Research Group Experimental Oto-Rhino-Laryngology Department of Neurosciences KU Leuven Leuven Belgium; 3 Psychological Sciences Research Institute Université Catholique de Louvain Louvain-la-Neuve Belgium; 4 Institute of Neuroscience Université Catholique de Louvain Louvain-la-Neuve Belgium

**Keywords:** serious gaming, language comprehension, enjoyment, feasibility, GameFlow

## Abstract

**Background:**

Enjoyment plays a key role in the success and feasibility of serious gaming interventions. Unenjoyable games will not be played, and in the case of serious gaming, learning will not occur. Therefore, a so-called GameFlow model has been developed, which intends to guide (serious) game developers in the process of creating and evaluating enjoyment in digital (serious) games. Regarding language learning, a variety of serious games targeting specific language components exist in the market, albeit often without available assessments of enjoyment or feasibility.

**Objective:**

This study evaluates the enjoyment and feasibility of a tablet-based, serious story-listening game for kindergarteners, developed based on the principles of the GameFlow model. This study also preliminarily explores the possibility of using the game to foster language comprehension.

**Methods:**

Within the framework of a broader preventive reading intervention, 91 kindergarteners aged 5 years with a cognitive risk for dyslexia were asked to play the story game for 12 weeks, 6 days per week, either combined with a tablet-based phonics intervention or control games. The story game involved listening to and rating stories and responding to content-related questions. Game enjoyment was assessed through postintervention questionnaires, a GameFlow-based evaluation, and in-game story rating data. Feasibility was determined based on in-game general question response accuracy (QRA), reflecting the difficulty level, attrition rate, and final game exposure and training duration. Moreover, to investigate whether game enjoyment and difficulty influenced feasibility, final game exposure and training duration were predicted based on the in-game initial story ratings and initial QRA. Possible growth in language comprehension was explored by analyzing in-game QRA as a function of the game phase and baseline language skills.

**Results:**

Eventually, data from 82 participants were analyzed. The questionnaire and in-game data suggested an overall enjoyable game experience. However, the GameFlow-based evaluation implied room for game design improvement. The general QRA confirmed a well-adapted level of difficulty for the target sample. Moreover, despite the overall attrition rate of 39% (32/82), 90% (74/82) of the participants still completed 80% of the game, albeit with a large variation in training days. Higher initial QRA significantly increased game exposure (*β*=.35; *P*<.001), and lower initial story ratings significantly slackened the training duration (*β*=−0.16; *P*=.003). In-game QRA was positively predicted by game phase (*β*=1.44; *P*=.004), baseline listening comprehension (*β*=1.56; *P*=.002), and vocabulary (*β*=.16; *P*=.01), with larger QRA growth over game phases in children with lower baseline listening comprehension skills (*β*=−0.08; *P*=.04).

**Conclusions:**

Generally, the story game seemed enjoyable and feasible. However, the GameFlow model evaluation and predictive relationships imply room for further game design improvements. Furthermore, our results cautiously suggest the potential of the game to foster language comprehension; however, future randomized controlled trials should further elucidate the impact on language comprehension.

## Introduction

### Background

Over the past 2 decades, there has been a major increase in the development of serious digital games for children with special educational needs [1,2]. This clear trend presumably relates to the popularity and motivational aspects of noneducational digital games and the increasing exposure to technological devices in children’s current daily lives [3]. Within the field of education, serious digital games are described as games for electronic devices with certain game rules and player outcomes, which aim to entertain but pursue at least one additional learning objective (eg, fostering mathematics, language, or reading) [2,4]. To obtain the desired educational impact from such games, children are often required to engage in interventions on a recurrent basis and for a considerable amount of time. Hence, game enjoyment, which triggers gameplay and increases the feasibility of maintaining playing [5,6], is key to the success of serious gaming interventions [7]. Thus, when evaluating serious games, it is of high relevance to assess not only the educational efficacy of interest but also the related aspects of game enjoyment and feasibility [2]. This study will address the enjoyment and feasibility of a newly designed serious story-listening game suitable for children aged 5 years. In addition, given the focus of the game on story listening, this study aims to preliminarily investigate its potential to train language comprehension [8].

Language comprehension is the process of interpreting spoken language input [9]. It involves speech processing at the basic auditory level, derivation of word meaning, knowledge of syntactic and morphological structures, and the integration of information within a broader language context [10]. Language comprehension creates opportunities for verbal communication in daily life and lays the foundations for reading comprehension long before receiving reading instruction [11,12]. Hence, unsurprisingly, language comprehension is a well-known precursor to academic achievement [8,13,14], employment [14,15], and social participation [14,16]. Individual differences in language comprehension already exist at an early age and remain relatively stable over time [17]. Hence, dramatically, without remediation, young children with poor comprehension are more likely to lag behind their peers throughout their entire development [17].

Problems with language comprehension are apparent in a high proportion of children with developmental language disorders (DLDs) [18]. DLD is characterized by severe problems with expressive and/or receptive language development in the absence of a clear-cut neural, cognitive, or auditory cause, affecting communication in daily life [19]. Remarkably, children diagnosed with DLD who experience comprehension deficits more often have persistent language problems [18], respond less well to interventions, and require more extensive support than children with DLD who exhibit language deficits in the expressive domain only [20]. In addition to DLD, lower comprehension skills have also been observed in second language learners [21], children in low socioeconomic status environments [22], children with hearing impairments [23], and children diagnosed with developmental dyslexia [24,25].

Language comprehension deficiencies benefit from interventions early in childhood [14]. Indeed, a recent systematic scoping review by Tarvainen et al [18] revealed that comprehension in young children is effectively tackled by (1) guidance in parent-child or clinician-child communication strategies or (2) by targeting receptive and expressive language aspects, such as vocabulary, morphology, and inferential language. Moreover, in young children aged <6 years, implicit therapy techniques, in which the child is exposed to optimal language without explicitly explaining certain rules, are preferred over explicit instructional approaches [26]. An example of a rather implicit approach concerns the method of storybook listening.

Given the evidence of storybook listening for fostering several language comprehension components in young children (eg, receptive vocabulary, morphosyntax, and narrative comprehension [27-29]), both in physical [30-33] or digital settings [34,35], to the best of our knowledge, no study has ever embedded the story listening method in a serious gaming context. Digital games often offer stimulating audiovisual game worlds and appealing rewards [36,37]. Given these motivational aspects [38,39], serious gaming is already widely applied in educational, psychological, and medical contexts [40-44], including the field of language learning (eg, game-based interventions specifically targeting vocabulary [45]). However, when designing a commercial serious digital game, detailed knowledge of game enjoyment is of crucial relevance. A digital game that is not experienced as fun will not be played [5], and consequently, in the case of serious gaming, learning will not occur. Hence, when evaluating serious games, both the aspects of enjoyment and educational impact must be considered [2]. However, despite its high relevance, the factor of enjoyment is hardly ever evaluated in existing serious games because of the lack of proper frameworks. To clarify the concept of game enjoyment as well as to facilitate its evaluation, Sweetser and Wyeth [5] proposed a summarizing framework—the GameFlow model—which is based on extensive gaming literature and the general theory of flow [46]. The model intends to guide serious game developers in the process of creating and improving games that are both educational and enjoyable [6]. More specifically, the GameFlow model [5] proposes eight interrelated gaming elements that are important for attaining overall game enjoyment: (1) concentration, (2) challenge, (3) player skills, (4) control, (5) clear end or intermediate goals, (6) feedback, (7) immersion, and (8) social interaction. The concentration principle states that a game becomes enjoyable when a player is able to concentrate on the game itself without being constantly distracted by surrounding background factors such as game and volume settings. The challenge principle argues that to enjoy a game, it must be sufficiently challenging. The player’s skills principle reasons that a game should provide opportunities to improve playing skills at a pace that is adjusted to the capacities of the individual player. The control principle asserts that players must experience a feeling of control over their actions, decisions, the appearance of their avatars and strategies, and the game world. The fifth clear end or intermediate goal principle states that a game must inform the player of clear end or intermediate goals from the start. The principle of feedback emphasizes the importance of appropriate feedback on game progress or errors at appropriate times. The immersion principle states that players must be completely absorbed by the game world. Finally, the social interaction principle underlines the relevance of social interactions during gameplay. To successfully embed each GameFlow element in a digital game, the GameFlow model [5] proposes a set of predefined implementation criteria for each element. For example, one of the criteria to attain the concentration principle states that game developers should not add irrelevant game tasks on top of the main learning task (refer to the study by Sweetser and Wyeth [5] for a full overview of the elements and their corresponding criteria). When evaluating games based on these GameFlow criteria, Sweetser and Wyeth [5] were able to successfully distinguish between low- and high-rated games. Thus, the validity of the GameFlow model in evaluating enjoyment in digital games is warranted.

### Objectives

This study addresses the enjoyment and feasibility of a newly developed serious tablet- and game-based story-listening intervention (henceforth, story game), for which the GameFlow model served as a guideline in the design process. Moreover, given the focus of the game on story listening, which is a frequently used method of increasing young children’s language comprehension [8], its potential to foster language comprehension will also be preliminarily investigated. The story game was originally developed to enhance basic auditory speech processing by modifying the recorded speech signals of stories with a so-called envelope enhancement (EE) algorithm [47-51] and, as such, boost phonology and reading in kindergarteners at cognitive risk for dyslexia. The EE algorithm automatically detects and amplifies important rhythmic acoustic cues of the speech envelope (ie, onset rise times), which are considered important for phonological skill (and potentially reading) development [52,53]. The story game was implemented in a broader preventive reading intervention aiming to (1) evaluate the efficacy of a tablet-based phonics intervention specifically targeting reading and phonology—GraphoGame Flemish (GG-FL) [54,55]—and (2) investigate the possible educational boosting effect of the EE story game on top of GG-FL, which will be addressed in a future study. Story game enjoyment will be evaluated in three ways based on (1) postintervention child and parental questionnaires, (2) fulfillment of the GameFlow criteria, and (3) in-game data indicative of game enjoyment. Feasibility will be addressed by analyzing the difficulty level of the game, attrition rate, and individuals’ final game exposure and training duration, together with their corresponding gaming profiles. Language comprehension growth will be addressed by exploring the accuracy of in-game story content-related questions as a function of game progress and baseline language subskills important for overall language comprehension (eg baseline vocabulary, morphological awareness, and listening comprehension [27-29]). Thus, we will test the hypothesis of whether language comprehension improves when progressing through the game while controlling for baseline language skills and also whether children with lower baseline language skills show larger growth potentials.

## Methods

### Participants and Ethics Approval

Following a school-based screening of 1225 children in the third year of kindergarten, 149 (12.16%) children aged 5 years (n=119, 79.9% children with and n=30, 20.1% children without an elevated cognitive risk for dyslexia) were enrolled in a game-based preventive reading intervention study (trial registration number S60962; assigned by the Clinical Trial Center of Universitair Ziekenhuis Leuven, Belgium). For the completed checklist on CONSORT-EHEALTH (Consolidated Standards of Reporting Trials of Electronic and Mobile Health Applications and Online Telehealth; version 1.6.1), consider Multimedia Appendix 1. A cognitive risk for dyslexia was assigned when a child scored above the 10th percentile on a nonverbal reasoning test [56] and below percentile 30 on minimally 2 out of 3 assessments of robust reading predictors (ie, letter knowledge [57], phonological awareness [57,58], and rapid automatized naming [59]). Typically developing children scored above percentile 40 on all reading precursors and were matched to the risk sample based on nonverbal reasoning ability, school environment, and gender. Consider the studies by Van Herck et al [49], Vanden Bempt et al [55], and Verwimp et al [60] for a more detailed description of the screening tasks, procedures, and participant selection. All the selected participants were in their third year of kindergarten, Flemish monolingual Dutch speaking, born in 2013, and had a schooling period of minimally 20 months. None of the selected children reported an additional behavioral or familial risk of attention deficit hyperactivity disorder, language or articulatory problems, severe hearing impairments, or neurological deficits. Furthermore, as Flemish schools only provide reading instructions from the first grade onward, all participants were considered to be prereading. This so-called prereading phase was also confirmed by a unanimous floor effect on a preintervention reading test [55]. Signed informed consent was obtained from all participants, and the study was approved by the medical ethical committee of Universitair Ziekenhuis Leuven, KU Leuven (Katholieke Universiteit Leuven; approval number B322201836276).

### Study Design and Procedure

Of the 149 children in the entire intervention study sample, 91(61.1%) at-risk children were asked to play the story game. Within the framework of the reading intervention study, these 91 children were randomly assigned to 1 of 3 experimental groups. The first group (GG-FL EE group; 31/91, 34%) played the story game with envelope-enhanced stories and combined it with a phonics-based GG-FL intervention [54]. For technical details of the EE algorithm, refer to the study by Van Herck et al [49]. The second group received the same intervention as the first, with the only difference being that no EE was applied to the stories in the story game (GG-FL nonenhanced group; 31/91, 34%). The third group also played the nonenhanced story game and combined it with tablet-based commercial Lego Duplo-, and Playmobil applications (henceforth active control [AC] games), which did not train any reading-related skills (AC nonenhanced group; 29/91, 32%). The intervention lasted 12 weeks/84 days in total and took place in the second semester of the last and third year of kindergarten. Of the 149 children, the remaining 28 (18.8%) at-risk and 30 (20.1%) non–at-risk children served as the at-risk passive control and the typically developing control group of the reading intervention study, respectively, and did not receive any type of digital gaming intervention. As this study mainly focuses on story game feasibility and enjoyment, the last 2 control groups, who did not play the story game, will not be discussed any further in this research paper. Before the intervention period, all the necessary games, including the story game, were installed on 91 tablets (Samsung Galaxy E9.6). All story sound levels of the story game were calibrated to be played at 60 dB-A over ATH-m20x headphones using speech-weighted noise that was constructed based on an average spectrum of the different storytellers [61]. Then, a variety of cognitive linguistic tasks, including baseline language skills (listening comprehension, receptive vocabulary, and morphological awareness), were individually assessed in all participants at school in a quiet test room. After this baseline test phase, the 91 at-risk children who were assigned to 1 of the 3 intervention groups received a tablet, a corresponding calibrated headphone, and a manual for parents with instructions to start the tablet intervention at home. With the support of a reward calendar with stickers, they were instructed to combine story game sessions with 15-minute GG-FL or AC sessions for 6 days per week over 12 weeks. This equaled 72 gaming sessions for both GG-FL or AC and the story game. After the intervention, the parents and children received a questionnaire to assess their motivation, enjoyment, and engagement during the intervention period.

### Story Game Intervention

#### Overall Game Description

The tablet-based story game application was developed in Unity 3D [62] and programmed entirely using the C# scripting language. For an overview of the rationale, developmental design-related decisions, stories, and programming details of the game, refer to the technical story game development guide (Multimedia Appendix 2 [5,47-53,58,62-79]). The story game provided 72 gaming sessions, each of which mainly comprised listening to 1 long (eg, approximately 10 minutes) or 2 short (eg, twice approximately 5 minutes) stories accompanied by story illustrations. Game settings prevented children from playing ≥1 session every 24 hours. The 72 sessions were categorized into 18 play phases of 4 grouped sessions containing 4 (longer) to 8 (shorter) stories from the same book series and author. In total, 87 stories from 14 different book series were implemented in the game. Generally, the game contained three main game modes: (1) the main intervention task environment, which involved actual story listening, story rating, and responding to content-related questions; (2) the virtual hub world, in which players consulted their game progress on a map; and (3) an avatar customization system, where players could buy accessories for their game avatars or buy new avatars. Most of the artwork in the game, such as 3D models, textures, and animations that were used to create the virtual hub world, the avatars, and their accessories, were custom-made; acquired from the Unity Asset Store [80]; or acquired from child-friendly projects of the Dyslexia Research Collaboration team at KU Leuven, such as Diesel-X [58]. The resulting art style was characterized by flat primary colors, avatars with exaggerated cartoon-like proportions, and simplified basic models and animations suitable for the chosen target age group [63,81].

#### Main Intervention Task Environment

**Figure 1:**
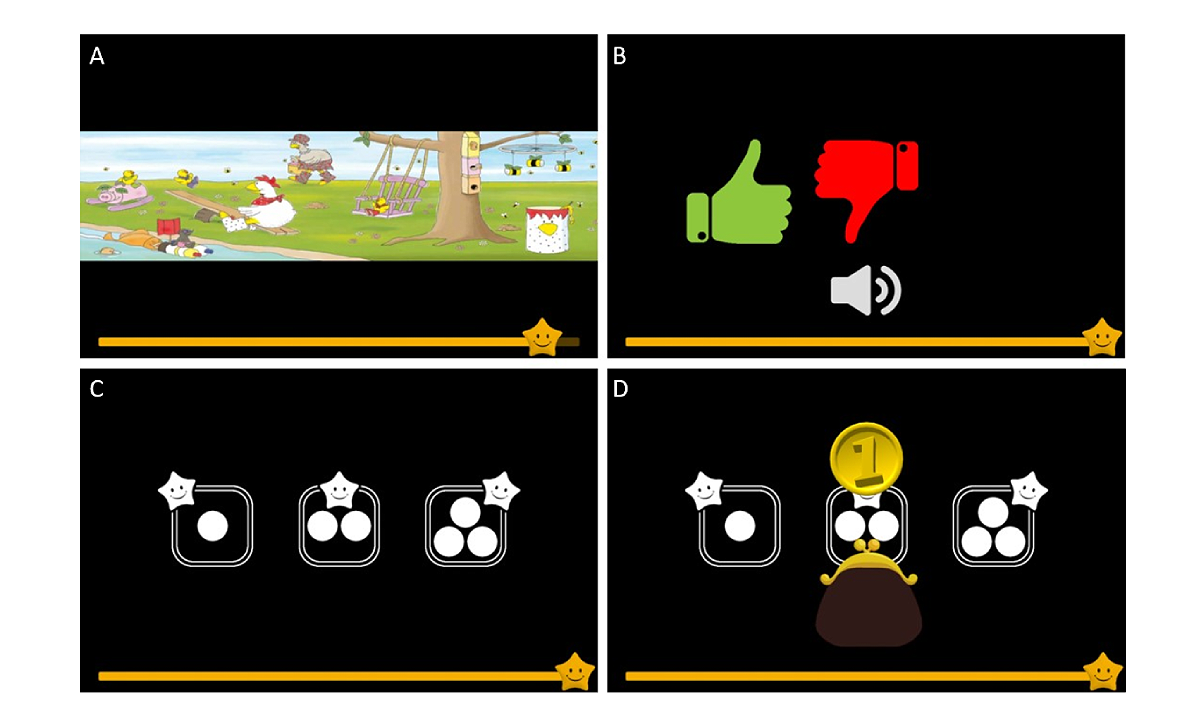
Main intervention task: (A) story listening (© 2016 Clavis Publishing, Hasselt–Alkmaar, New York; illustrations from Lotta ruimt het op from Diane Put and Rik De Wulf); (B) story rating; (C) multiple-choice questions; (D) reward for each correct response.

The main intervention task presented recorded story audio, all in Dutch, with accompanying illustrations (Figure 1A), followed by a story appreciation rating (Figure 1B) and audio questions with a simple multiple-choice system (Figure 1C). Questions were formulated by the research group, and listening comprehension was examined by recalling information explicitly mentioned in the story text. Although not directly assessed, the language level of the questions was intended to match the language and cognitive capacities of children aged 5 years. The question part put a strong emphasis on feedback by awarding 1 coin per correct response, with added flair in the form of animations and sound effects (Figure 1D). Each session contained 3 content-related questions. Hence, given the total amount of 72 game sessions, players could maximally earn 216 coins throughout the game.

#### Virtual Hub World

**Figure 2:**
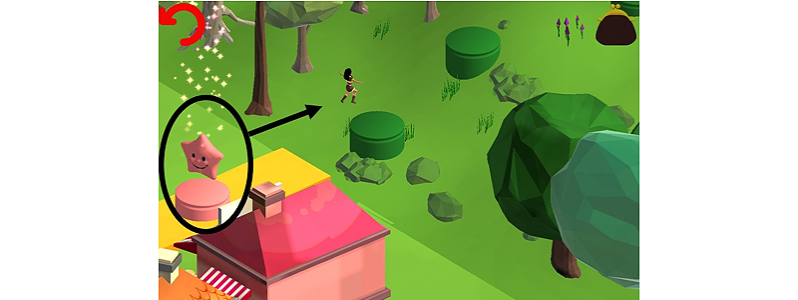
Completion of a game session shown in the virtual hub world.

Similar to classic digital games such as Super Mario Bros, the different game sessions were tied together in a so-called virtual hub world. The virtual hub world provided an overview of each game session in the form of a cylindrical stage. Every time a player successfully completed a game session, his or her avatar jumped from one cylindrical stage to the next, leaving a star on the stage that was just abandoned (Figure 2). As such, players gained information on their progress throughout the game and could earn a maximum of 72 stars (ie, the total number of game sessions).

#### Avatar Customization System

**Figure 3:**
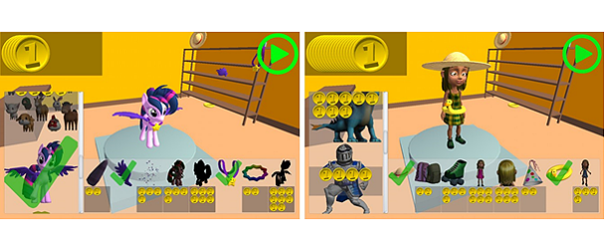
Avatar customization system of the story game.

To provide added incentives and rewards to keep the player engaged, an avatar customization system was implemented, which featured a virtual store where players could spend their coins (Figure 3). These coins were earned through correct responses to the content-related story questions in the main intervention task. The store offered new avatars and accessories for current avatars, which were displayed in the virtual hub world. The items put an emphasis on quantity to make sure that the player could acquire roughly one new item per story session without running out long before reaching the end of the story game.

#### Story Game Session

In a single game session, first, players watched a short cut-scene of their avatar positioned on one of the 72 possible stages in the virtual hub world, representing the 72 game sessions and, thus, their progress throughout the game. Second, the main intervention task occurred, in which the player was required to actively listen to 1 or 2 stories, depending on the particular story session. After listening, the player rated the story based on a green or red thumb (ie, like or dislike, respectively) and received 3 content-related multiple-choice questions for which one coin could be earned per correct response. Following the short quiz, the player automatically ended up in the avatar customization system, where the earned coins could be spent on accessories for the avatar or to buy extra avatars. Finally, the player was sent back to the hub world in which the new or newly decorated avatar jumped to the subsequent cylindrical stage while a dancing star appeared on the one that was just abandoned (for a demonstration movie of the story game, see Multimedia Appendix 3). Useful player data for individual player accounts (eg, game progress, question response accuracy [QRA], play dates and hours, and story rating information) were automatically logged on our university server and sent daily to the research group.

### Test Battery and Questionnaires

#### Baseline Listening Comprehension

The subtest *Understanding Spoken Paragraphs* was adapted from the Clinical Evaluation of Language Fundamentals (CELF)-fourth edition, Dutch version [82] and was used to evaluate baseline listening comprehension. The experimenter read 2 short stories aloud. Each story was followed by 5 content-related questions. The child received 1 point per correct response. The maximum score on the test was 10.

#### Baseline Receptive Vocabulary

Receptive vocabulary was measured using the Peabody Picture Vocabulary Test–III, Dutch version [83], which contains 17 subtests with 12 test trials each. In each trial, the child heard a target word and was asked to select the corresponding picture from the 4 alternatives. The test was interrupted when the child made ≥9 errors in 1 subtest. The raw receptive vocabulary score was computed by subtracting the sum of the errors across all administered subtests from the number of trials that was last assessed. This raw score was then converted into a standard score and represented the final receptive vocabulary score (mean 100, SD 15).

#### Baseline Morphological Awareness

The *Word Structure* subtest from the CELF Preschool–second edition, Dutch version [84] was used to evaluate morphological awareness. In this task, the ability to correctly apply Dutch morphological rules (eg, conjugation, derivation, flexion, pronouns, and degrees of comparisons) was measured. The test contained 23 items, each containing 2 pictures. The first picture was fully described by the experimenter (eg, “Dit meisje eet” meaning “This girl eats”) followed by an incomplete description of the second picture (eg, “en dit meisje” meaning “and this girl…”), which had to be completed by applying a certain morphological rule of Dutch (eg, “and this girl sleeps” or “en dit meisje slaapt”; morphological rule: conjugation of a regular verb in third singular form). On the basis of the instructions in the test manual, the test was interrupted when the child made 7 consecutive mistakes. The maximum score was 23.

#### Postintervention Child and Parental Questionnaires

After the intervention period, parents independently filled out a short questionnaire that included three questions related to motivation, encouragement, and sustained attention during story gameplay. Accompanied by a member of the research group at school, intervention enjoyment was also measured in all children using 2 components of the Fun Toolkit survey instrument [85], which was developed to gather children’s opinions on technology. On the one hand, children were asked to assign story game enjoyment on a 5-point Likert scale–based smiley-o-meter, in which the 5 scales were represented by smileys (ranging from a very unhappy smiley meaning *I did not like the game at all* to a very happy smiley meaning *I liked the game very much*). On the other hand, participants were asked whether they were willing to redo the intervention (see Multimedia Appendix 4 for an overview of the 5 child and parental categorical questions and their response possibilities).

### Statistical Analysis

#### Data Exclusion

Of the 91 children who were asked to play the story game, 7 (8%) were excluded from the data set because of technical game problems during the intervention period (bugs; explained in (Multimedia Appendix 2 [5,47-53,58,62-79]). Approximately 2% (2/91) of children were excluded as they never started the general digital gaming intervention properly (ie, they played <10% of the GG-FL or AC and the story game). Hence, useful story game data were available for 90% (82/91) of the children. Data visualization and statistical analyses were conducted using RStudio [86,87].

#### Enjoyment Analysis

Story game enjoyment was addressed in 3 ways. First, the postintervention child and parental questionnaire outcomes were analyzed by investigating the relative frequencies per response category of each question. Second, 2 members of the research group independently evaluated the GameFlow criteria by assigning a state of fulfilled, partly fulfilled, or not fulfilled. Then, after reaching a consensus, all criteria received a final, single state. Third, in-game enjoyment–related data were evaluated, such as general story appreciation, which was measured as the proportion of given likes and dislikes per story.

#### Feasibility Analysis

To gain insights into the feasibility of the intervention, we first defined the general QRA, which indicated the difficulty level and thus the feasibility of the story-listening part of the game. This was computed as the proportion of correct and incorrect responses per question. Second, we visualized the attrition rate, which determined the number of children who completed the intervention and at what point eventual dropouts occurred. Third, we calculated the individuals’ final game exposure and training duration. Final game exposure was computed as the ratio (percentage) of the number of actually played sessions to the total number of available sessions (ie, n=72). The individuals’ corresponding final training duration represented the number of days the child played, starting from the day of the first story session until the day on which the participant played for the last time, irrespective of game completion. The measure of the final training duration was particularly informative about the feasibility of the training intensity. By analyzing the players’ final game exposure and training duration, we categorized players into different gaming profiles based on intervention completion (complete or incomplete) and schedule compliance (compliant or noncompliant). Categorizing participants based on these criteria provided information on the possible need to adjust the training schedule and intervention duration to increase the feasibility of future studies. A total of 4 additional nonparametric median-based Theil–Sen regressions were performed using the *mblm* package in R [88] to predict individuals’ final game exposure or final training duration, either based on initial individual story appreciation or QRA. These analyses provided information on how to keep players engaged from the start and prevent them from dropping out or slowing down. The Theil–Sen technique allows for a robust line fitting as the estimation is obtained by calculating the median of the slopes of all possible pairs of data points [89]. This nonparametric regression technique was opted for, given the relatively small sample size and violated assumptions to perform ordinary least square regressions. Initial individual story appreciation and individual response accuracy at the start of the intervention were defined as a player’s mean story appreciation and mean QRA in the first two game phases, respectively (ie, mean ratings of the first 12 stories and mean response accuracy for the first 24 questions). As for predicting game exposure based on initial story appreciation or initial individual QRA, the analyses were restricted to a sample of players who did not completely finish the story game (32/82, 39%), as the inclusion of participants who completed the full intervention would render a ceiling effect in the results. As for predicting the training duration based on initial story appreciation or individual response accuracy, we excluded 10% (8/82) of participants who played <80% of the total game content, as their training duration in days did not represent a reliable intervention trajectory. As such, the training duration was predicted based on the data of 90% (74/82) of participants.

#### Analyzing Growth in Language Comprehension

The third and last part of the *Results* section tackles a possible growth in language comprehension. We considered the mean QRA (percentage) per game phase as an indicator of language comprehension. However, we acknowledge that stories and questions do not belong to a validated language instrument, and we did not directly test the stability of the difficulty of the questions and stories. However, we were obliged to select measures beyond the existing language instruments, given the long intervention period. Each game phase contained 12 content-related questions. Using the *lmerTest* package in R [90], we performed a multilevel linear growth model in the sample that finished at least 80% of the game (74/82, 90%), with game phase as a within-subjects variable and baseline listening comprehension, morphological awareness, and receptive vocabulary as between-subjects covariates. The game phase was coded such that the fixed intercept of the model represented the mean QRA of the first game phase if a score of zero was obtained for all 3 baseline language covariates. We also modeled a randomly varying intercept and slope across individuals and added 3 additional phase×baseline language interaction terms (1 per baseline language covariate). As such, we could test the hypothesis that the mean QRA, which was our indication of language comprehension, improved over the game phase when controlling for each baseline language skill, as well as whether children with lower baseline language skills showed larger growing potentials concerning mean QRA.

## Results

### Enjoyment

#### Postintervention Questionnaires

Postintervention child questionnaire data revealed that 74% (61/82) of participants liked the game very much. Moreover, 66% (54/82) of participants confirmed that they were willing to redo the training. Postintervention parental questionnaires revealed (1) that most parents observed a high (39/82, 47%) or relatively high play motivation (32/82, 39%) in their child, (2) that most of the players never (43/82, 52%) or only sometimes (25/82, 30%) needed encouragement to play, and (3) that more than three-quarters of the parents (65/82, 79%) observed a state of sustained attention during gameplay.

#### GameFlow Criteria Fulfillment

Although the GameFlow model was applied in the design process, Tables 1 to 8 clarify the extent to which the game criteria were properly implemented and whether our game did, only partly, or did not fulfill each GameFlow criterion [5]. As shown in Tables 1 to 8, the GameFlow model–based evaluation revealed that only the criteria belonging to the concentration principle were completely fulfilled. As for the other elements, except for the elements of social interaction (as it was intentionally not implemented, given the young age of our participants) and challenge, we concluded that most of their corresponding criteria were completely or partially fulfilled. To avoid plagiarizing from Sweetser and Wyeth [5], the wording of the descriptions of the GameFlow criteria in Tables 1 to 8 differs from the criteria wording used in their article [5], although the meaning of the criteria did not change. However, we want to emphasize that we considered the original wordings when developing and evaluating the story game, as we wanted to take into account the importance of applying evaluation tools in the exact same way as they are validated.

**Table 1:** Corresponding criteria of the GameFlow element concentration and their implementation and fulfillment in the story game.

Concentration criteria	Implementation in the story game	Fulfillment status
Stimuli are provided in different modalities	Stimuli were aurally and visually presented both in the main intervention task (eg, story audio and accompanying illustrations) as well as in the virtual hub world and avatar customization system (eg, simple tunes and visual animations) at all times.	Yes
Stimuli are worth attending to	Story texts and corresponding story images were selected based on the target age. The virtual hub world and the avatar customization system provided joyful, simple tunes and appeared with flat primary colors and simplified basic models and animations, which are suitable for our target age group [63,81]. Avatars were designed with exaggerated cartoon-like proportions. As such, stimuli seemed appealing, eye catching, and worth attending to for a long time.	Yes
Games must quickly catch and hold the players’ attention and must be able to maintain focus at all times	Story texts and corresponding story images were selected based on the target age. The virtual hub world and the avatar customization system provided joyful, simple tunes and appeared with flat primary colors and simplified basic models and animations, which are suitable for our target age group [63,81]. Avatars were designed with exaggerated cartoon-like proportions. As such, stimuli seemed appealing, eye catching, and worth attending to for a long time.	Yes
There are no irrelevant game tasks on top of the main task	When children were required to listen to the story (ie, the main learning task), the game did not require performing other irrelevant side tasks.	Yes
Games should require cognitive workload but should not exceed perceptual and cognitive limits	Players were required to actively listen and stay focused during the whole story-listening phase, as they could only collect rewards (coins) when they responded correctly to the content-related questions. However, stories and content-related questions were chosen to correspond to the cognitive limits of kindergarteners aged 5 years. The virtual hub world and avatar customization system warranted easy-to-use mechanics and simple, colorful animations so that players did not become *lost in the game world* or overstimulated.	Yes
There are no distractors during the game tasks	Tasks in the main intervention and avatar customization phase were clearly defined, such that, when performing the tasks (eg, actively listening to the story, responding to the questions, or spending coins in the avatar customization system), players were not able to perform any other tasks and were not distracted by irrelevant stimuli.	Yes

**Table 2:** Corresponding criteria of the GameFlow element challenge and their implementation and fulfillment in the story game.

Challenge criteria	Implementation in the story game	Fulfillment status
Challenges are adjusted to players’ skills	As the research group selected age-appropriate stories and invented literal questions that were supposed to match the cognitive skills of kindergarten children aged 5 years and as the virtual hub world and avatar customization system appeared self-explanatory without any form of written language, we concluded that the game tasks were adapted to the skills of the target prereading age group.	Yes
Challenges are player-adjusted	The selection and the order of the stories, along with their corresponding content-related questions, were fixed and the same for all players. Moreover, although we have no direct proof of the stability of the difficulty of the stories and questions, the game was originally not designed to increase in difficulty. In that sense, we concluded that the challenge levels were not individually adapted and did not increase along with progress in player skills.	No
Challenge levels increase to improve the players’ skills	The selection and the order of the stories, along with their corresponding content-related questions, were fixed and the same for all players. Moreover, although we have no direct proof of the stability of the difficulty of the stories and questions, the game was originally not designed to increase in difficulty. In that sense, we concluded that the challenge levels were not individually adapted and did not increase along with progress in player skills.	No
The game provides new challenges at appropriate times	Although not directly tested, we assumed that the difficulty level of the stories, the questions, and other tasks remained relatively stable throughout the game. However, book series and storytellers switched every 4 sessions. This required adaptability and possibly formed a new challenge for some (but not all) players.	Partly

**Table 3:** Corresponding criteria of the GameFlow element player skills and their implementation and fulfillment in the story game.

Player skills criteria	Implementation in the story game	Fulfillment status
The game is playable without an instructive manual	Although the research group provided a short manual for the players’ parents or caregivers, the game was designed in a way that children aged 5 years could pass the first and following sessions without any form of explanation. Game mechanics were easy to use, transfers from one game mode to another occurred automatically, and as the target group was considered prereading, there was no form of written text implemented in the game.	Yes
Learning the game is not boring	A learning phase or tutorial game was not implemented, as game sessions were self-explanatory, and players learned to play by doing. When opening the application on the tablet for the first time, players gained enough information on how to progress. Hence, we believed that this criterion did not necessarily need to be implemented.	No
Absorbing tutorial games should teach players how to play the game	A learning phase or tutorial game was not implemented, as game sessions were self-explanatory, and players learned to play by doing. When opening the application on the tablet for the first time, players gained enough information on how to progress. Hence, we believed that this criterion did not necessarily need to be implemented.	No
Web-based help is provided for players in need	The research group did not provide a web-based help service tool. However, parents or caregivers could contact the research group via email or telephone anytime in case of technical or motivational problems. If necessary, a member of the research group visited the players at home to fix possible technical or player-related problems.	Partly
Skill progress occurs gradually at an appropriate pace	Although not directly tested, we assumed that the difficulty level of the questions and other tasks remained relatively stable throughout the game and that they were adapted to the target age. The game did not primarily intend to specifically improve language comprehension (measured based on mean question response accuracy per game phase) of the target group. However, it is possible that for some of the players, mean question response accuracy would gradually increase with higher story game exposure. This will be explored in this paper.	Partly
Skill effort and development is rewarded	Skill effort was rewarded by means of stars. After finishing a session, regardless of whether the responses to the content-related questions were correct or not, all players earned a star, which appeared on a stage in the virtual hub world and indicated the players’ progress in the game. Skill development was rewarded with coins, such that the more correct responses were given, the more coins were earned.	Yes
Game mechanics and interface are simple and easy to apply	All actions required the use of a touch screen, as the game was tablet based. Nowadays, in Western society, most children aged 5 years are familiar with these devices. Furthermore, players only had a limited set of actions, as advanced game options were locked with a password. These limited actions (eg, stopping, starting, or continuing the story game; selecting a response for the questions; buying accessories in the avatar customization system) were all self-explanatory (eg, selecting the correct response or a desired accessory by touch screen) or assigned with clear symbols on the screen (red arrow to stop the game and large green play symbol to start or continue the story recording).	Yes

**Table 4:** Corresponding criteria of the GameFlow element control and their implementation and fulfillment in the story game.

Control criteria	Implementation in the story game	Fulfillment status
A sense of control over the game avatar and its movements and interactions	During the main intervention task, it was not possible to exercise control over the game avatar. However, following the main intervention task, players were able to customize their avatar or buy new avatars in the avatar customization system, all of which or whom appeared later on the platforms in the virtual hub world. However, as the avatars automatically jumped to the next stage when finishing a session, their movement and interaction control was limited.	Partly
A sense of control over the game interface and device	As children aged 5 years are relatively familiar with tablets in recent times, we believed that the tablet was a suitable device for the intervention program. Moreover, individual player profiles were set in advance on each tablet, and the main menu contained no other option than starting or stopping the game, making the game interface intuitive and self-explanatory.	Yes
A sense of control over the game mechanics (saving and stopping)	Players were able to start and stop the game anytime. However, when interrupting the game during story listening, the progress of the ongoing session was not registered or saved, and players should restart the whole session.	Partly
Errors that will harm the game (eg, bugs) should not occur, and if they occur, support must be available	The game was piloted many times on members of the research group and on children with a similar age as the target group. Although the game appeared bug free at the end of the pilot studies, some players in the actual intervention study (not included in the final data analysis) experienced bugs causing a total crash of the game and loss of in-game data. In those cases, a member of the research group provided support as soon as possible by reinstalling the game at the homes of the players.	Partly
A sense of control over the game world (possibility to shape the game world)	Following the main intervention task and the time spent in the avatar customization system to change the game avatar, the newly dressed or new game avatar appeared in the virtual hub world on one of the stages. In that sense, players exercised control over altering their game world. However, the background animations and models in the hub world were preprogrammed and could not be altered.	Partly
A sense of control over the game actions and strategies	As the order and goal of the game, along with the framed intervention task, was fixed for all players, it was not possible to make use of different strategies and actions to reach the end of the game.	No

**Table 5:** Corresponding criteria of the GameFlow element clear goals and their implementation and fulfillment in the story game.

Clear goals criteria	Implementation in the story game	Fulfillment status
Final goal is clear from the start	Before the very first session, players viewed the virtual hub world from a *drone’s perspective* and flew over all 72 stages that their game avatar had to pass to finish the game. That way, the player was immediately informed of the total amount of sessions. However, as the drone cut-scene was only presented at the start without any form of explanation, the final goal (eg, earning 72 stars) was not clearly emphasized during the entire intervention period.	Partly
Intermediate goals are clear	After playing the first session, it was clear for the player what should be reached during one game session.	Yes

**Table 6:** Corresponding criteria of the GameFlow element feedback and their implementation and fulfillment in the story game.

Feedback criteria	Implementation in the story game	Fulfillment status
Feedback on progress	After every session, feedback on game progress was visually provided by the game avatar, who jumped from one stage to the next in the virtual hub world. A star appeared on the platform that was just left. When the avatar reached the 72nd platform, all game sessions were finished.	Yes
Immediate feedback on actions	A star was given to all players as immediate feedback on the session completion. When responding correctly to a content-related question, the player received a coin, which was immediately presented after assigning the correct answer with animations and sound effects. Moreover, newly bought accessories or avatars immediately appeared in the virtual hub world.	Yes
Status or score is presented at all times	The number of collected stars was only visible in the main menu. The amount of earned coins was visible in the avatar customization system or could be considered when clicking on a little wallet that was visible in the virtual hub world. Thus, during the main intervention task, no score status was presented as we wanted to prevent players from being distracted by irrelevant stimuli.	No

**Table 7:** Corresponding criteria of the GameFlow element immersion and their implementation and fulfillment in the story game.

Immersion criteria	Implementation in the story game	Fulfillment status
Decreased awareness of surroundings when playing the game	Although not measured and not measurable with questionnaires in our young target age group, child and parental questionnaire outcomes revealed that most of the players liked the game very much, were motivated to play, and would be willing to play again. Moreover, most of the parents or caregivers indicated a state of full focus when their child played the game, suggesting that some form of immersion was present in most of the players.	NM^a^
Decreased self-awareness and less everyday worries when playing the game	Although not measured and not measurable with questionnaires in our young target age group, child and parental questionnaire outcomes revealed that most of the players liked the game very much, were motivated to play, and would be willing to play again. Moreover, most of the parents or caregivers indicated a state of full focus when their child played the game, suggesting that some form of immersion was present in most of the players.	NM
A changed sense of time	Although not measured and not measurable with questionnaires in our young target age group, child and parental questionnaire outcomes revealed that most of the players liked the game very much, were motivated to play, and would be willing to play again. Moreover, most of the parents or caregivers indicated a state of full focus when their child played the game, suggesting that some form of immersion was present in most of the players.	NM
Emotional involvement in the game	Although not measured and not measurable with questionnaires in our young target age group, child and parental questionnaire outcomes revealed that most of the players liked the game very much, were motivated to play, and would be willing to play again. Moreover, most of the parents or caregivers indicated a state of full focus when their child played the game, suggesting that some form of immersion was present in most of the players.	NM
Visceral involvement in the game	Although not measured and not measurable with questionnaires in our young target age group, child and parental questionnaire outcomes revealed that most of the players liked the game very much, were motivated to play, and would be willing to play again. Moreover, most of the parents or caregivers indicated a state of full focus when their child played the game, suggesting that some form of immersion was present in most of the players.	NM

^a^NM: not measured.

**Table 8:** Corresponding criteria of the GameFlow element social interaction and their implementation and fulfillment in the story game.

Social interaction criteria	Implementation in the story game	Fulfillment status
Competition between different players	The game did not intend to create competition between different players as participants in this paper were recruited anonymously and independently from each other.	No
Social interaction between different players (eg, chat services)	This was not applicable for our target age group of children aged 5 years.	No
Available social communities about the game	This was not applicable for our target age group of children aged 5 years.	No

#### In-Game Data Related to Game Enjoyment

**Figure 4:**
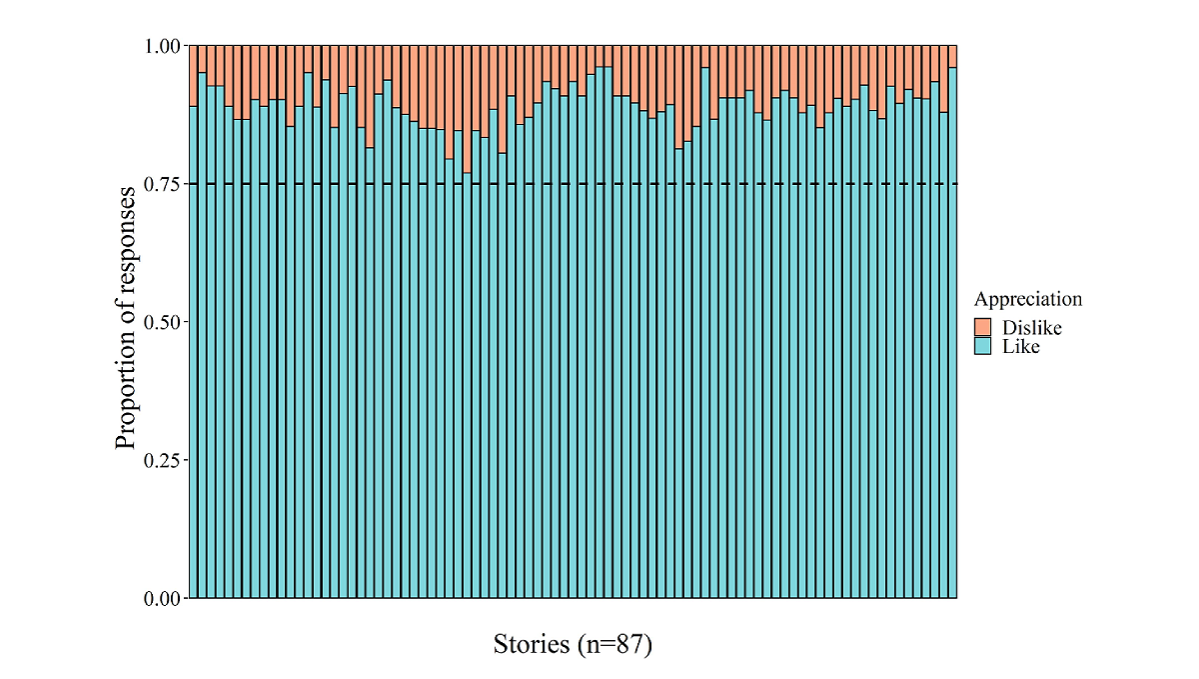
Overview of the general story appreciation. Each vertical bar represents one story, and the stories are ordered based on their occurrence in the game.

Figure 4 visualizes the general story appreciation, calculated as the proportion of available likes and dislikes per story. Each story was liked by >75% (ie, >62/82 listeners) of the listeners for whom data were available, indicating that the stories were enjoyable.

### Feasibility

#### General QRA

**Figure 5:**
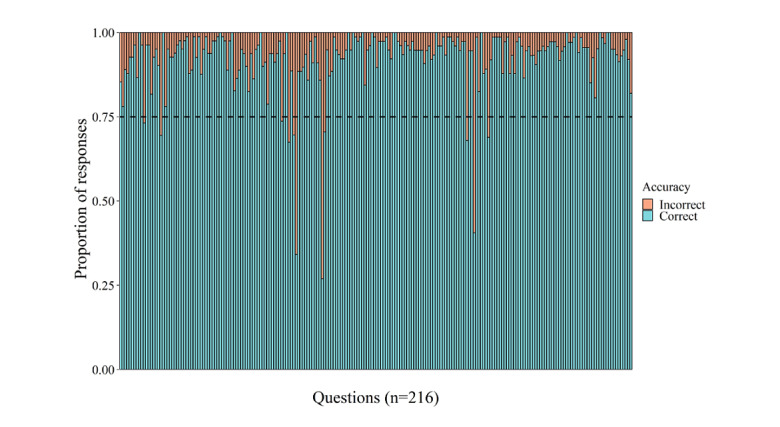
Overview of the general question response accuracy. Each vertical bar represents one question, and the questions are ordered based on their occurrence in the game.

Figure 5 visualizes the general QRA, calculated as the proportion of correct and incorrect responses per question. All but 11 questions were answered correctly by at least 75% (62/82) of the listeners for whom data were available, suggesting a rather stable difficulty level for the questions.

#### Attrition Rate

**Figure 6:**
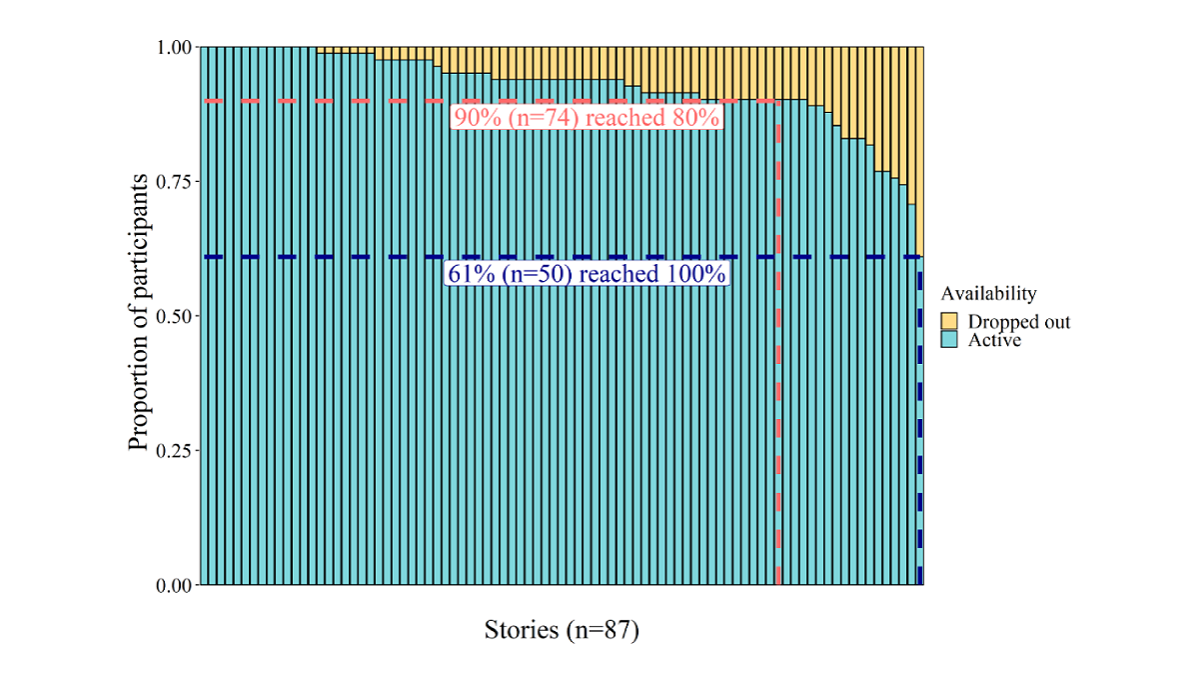
Attrition rate and dropout occurrence throughout the story game. Each vertical bar represents one story, and the stories are ordered based on their occurrence in the game.

Figure 6 shows the attrition rate and the proportion of active participants and dropouts throughout the entire story game progress. Of the 82 players, 74 (90%) listened to 80% of the stories (approximately 57th session of the total 72 story sessions), and 50 (61%) managed to finish the game completely. Hence, 39% (32/82) of players dropped out at some point during the 12-week intervention. Figure 6 also shows that the first player dropped out after the 12th story, corresponding to the start of the third game phase. From that point onward, the dropout proportion stayed relatively stable until the 80% completion point, after which it gradually increased toward the end of the game.

#### Final Game Exposure, Training Duration, and Gaming Profiles

**Figure 7:**
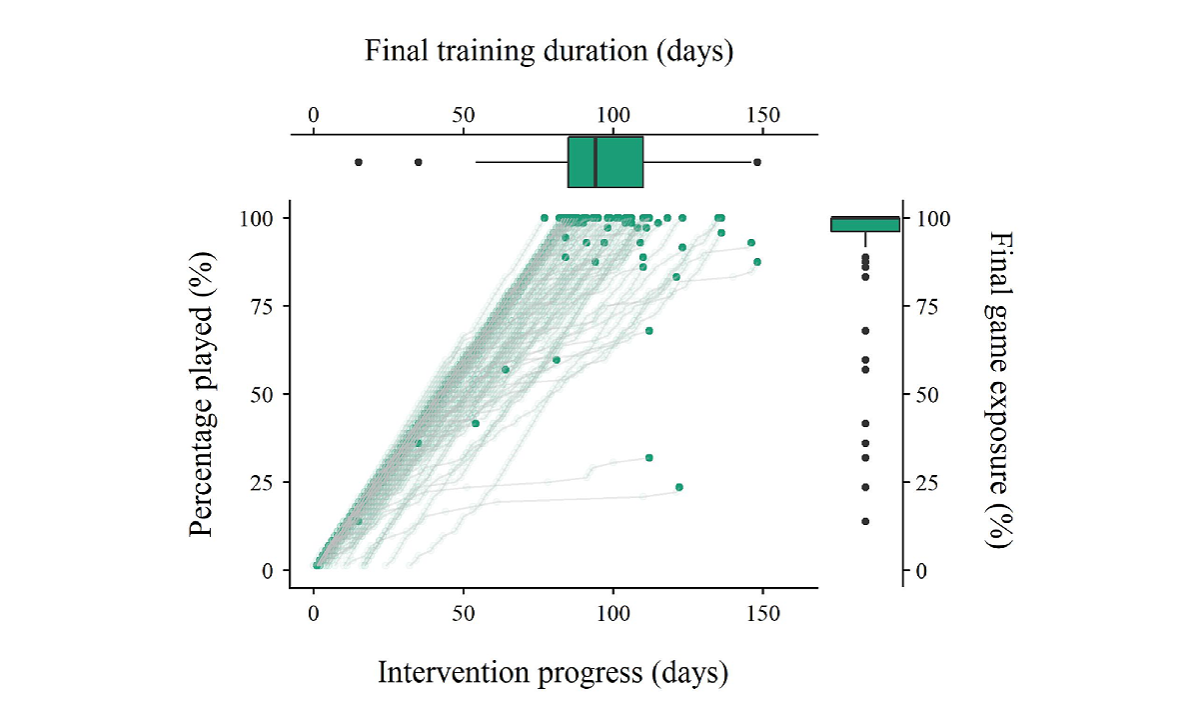
Individuals’ game exposure and training duration and their overall distributions. Bold dots represent the final training duration and final game exposure.

**Figure 8:**
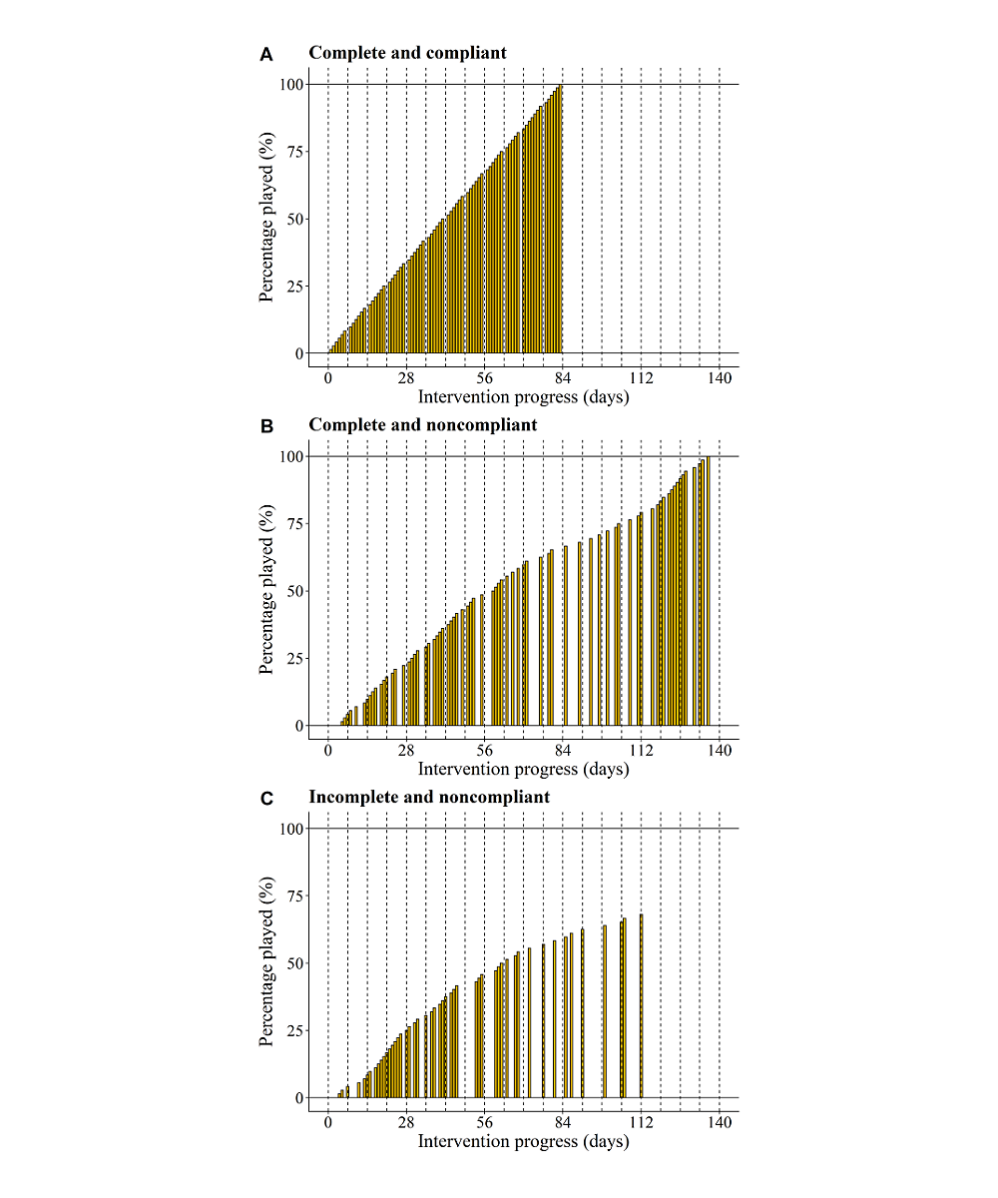
Examples of the different gaming profiles of the story game. Complete and compliant player (A). Complete and noncompliant player (B). Incomplete and noncompliant player (C). Dotted lines represent the number of intervention weeks.

Figure 7 demonstrates individual story game trajectories, individuals’ final game exposure and corresponding training duration, and their overall distributions. Apart from the visible finding that a few participants had a low training duration as they dropped out early, Figure 7 also shows a large variation in the training duration in children who nearly or completely finished the story game. On the basis of the categorization criteria (complete or incomplete and compliant or noncompliant), we established three different gaming profiles: (1) complete and compliant players (18/82, 22%; Figure 8A), (2) complete but noncompliant players (32/82, 39%; Figure 8B), and (3) incomplete and noncompliant players (32/82, 39%; Figure 8C). Note that we did not observe any players who perfectly followed the training schedule but dropped out at some point.

**Figure 9:**
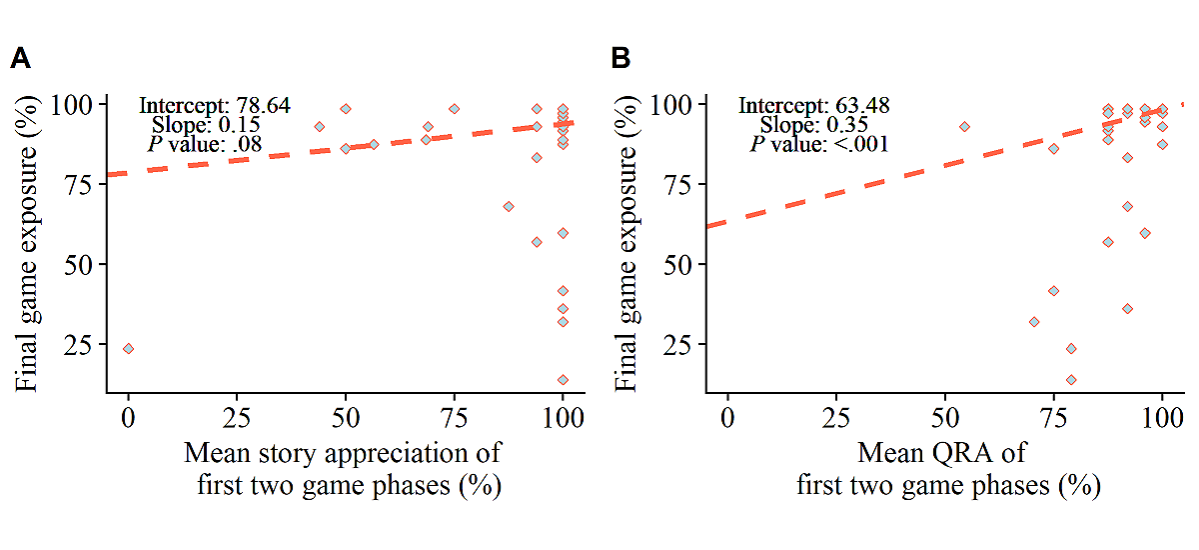
Theil–Sen regression outcomes. Predictive relationship between initial mean story appreciation and final game exposure (A). Predictive relationship between initial mean question response accuracy (QRA) and final game exposure (B).

The Theil–Sen regression analysis revealed that the mean story appreciation of the first 2 game phases was not significantly predictive of final game exposure among the 39% (32/82) of children who dropped out at some point (*β*=.15; *P*=.08). Figure 9A shows that most children who quit at some point still assigned positive ratings to all stories of the first 2 phases. In contrast, the Theil–Sen regression analysis revealed that the mean QRA of the first 2 game phases significantly predicted final game exposure, such that a lower accuracy at the start accelerated the dropout point (*β*=.35; *P*<.001; Figure 9B).

**Figure 10:**
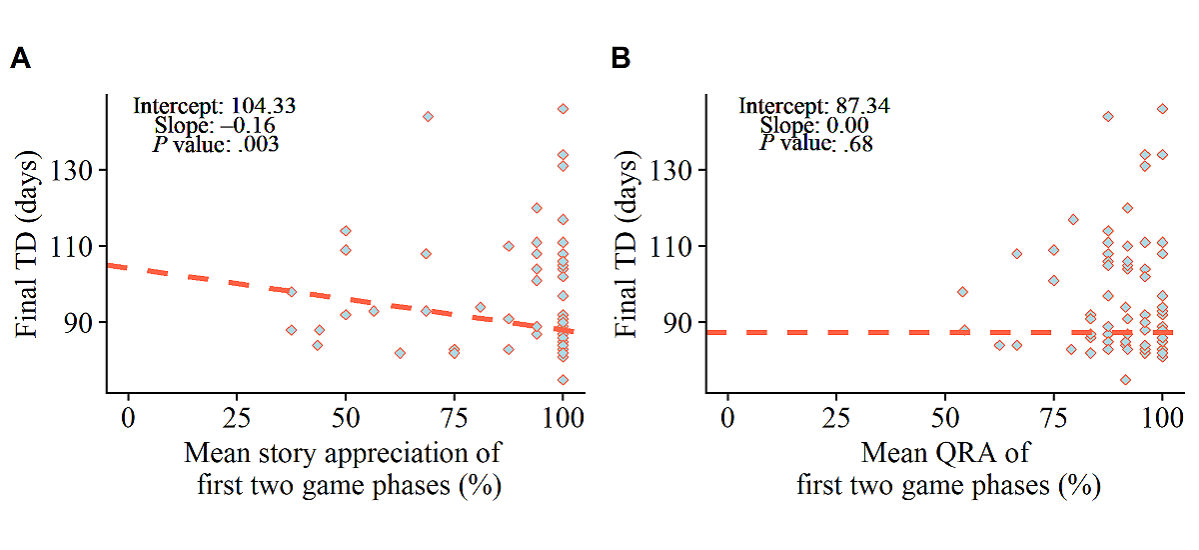
Theil–Sen regression outcomes. Predictive relationship between initial mean story appreciation and final training duration (A). Predictive relationship between initial mean question response accuracy (QRA) and final training duration (B). TD: training duration.

Story appreciation in the first 2 game phases significantly predicted the final training duration in a negative way among the 90% (74/82) of children who finished 80% of the game (*β*=−0.16; *P*=.003; Figure 10A). These results indicate that players who liked the stories at the start of the game were more likely to play the game at a higher rate. The mean QRA at the start did not significantly predict the final training duration (*β*=.00; *P*=.68) among children who finished or almost completely finished the story game (Figure 10B).

### Growth in Language Comprehension

**Figure 11:**
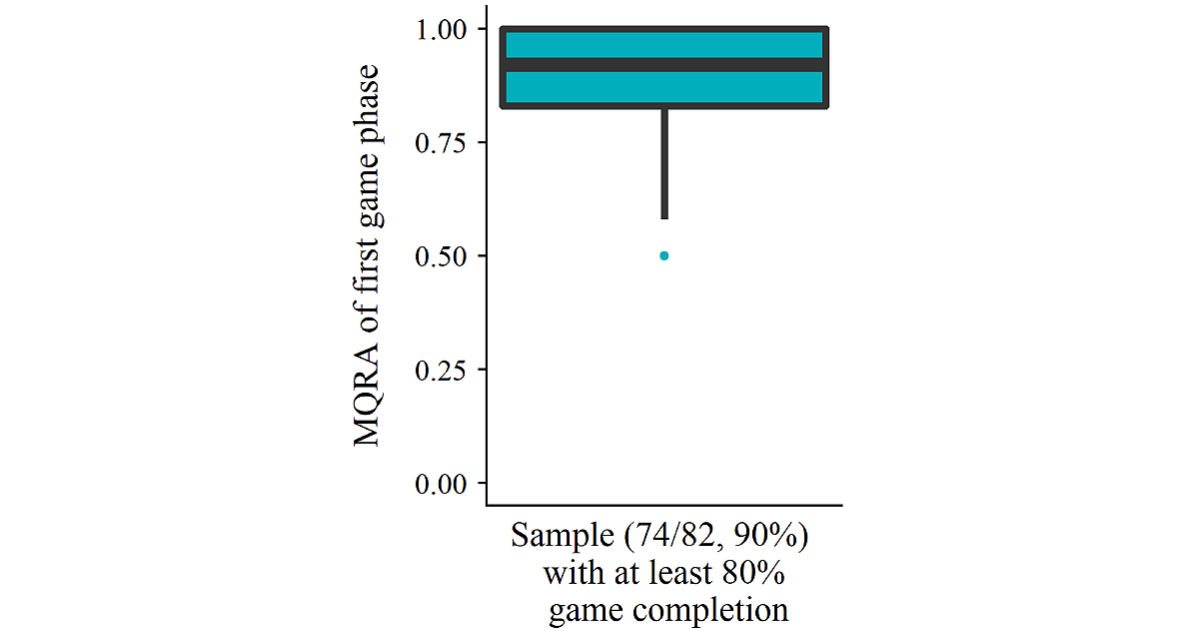
Distribution of the mean question response accuracy (MQRA) of the first game phase.

Figure 11 presents the distribution of the mean QRA of the first game phase in all children (74/82, 90%) who completed at least 80% of the game and shows overall high initial accuracy scores, suggesting a small growing potential in a large proportion of children. The outcomes for the linear mixed effects model are presented in Table 9. The game phase significantly predicted mean QRA in a positive direction (*β=*1.44, SE 0.49; *t*_120.12_=2.974; *P*=.004). Moreover, there was a positive predictive relationship between baseline listening comprehension (*β=*1.56, SE 0.49; *t*_71.91_=3.168; *P*=.002) and receptive vocabulary (*β=*.16, SE 0.06; *t*_72.27_=2.64; *P*=.01) on mean QRA. Remarkably, the results also revealed a significant interaction between the game phase and listening comprehension, such that children with lower baseline listening comprehension underwent a significantly larger growth in mean QRA throughout the game (*β=*−0.08, SE 0.04; *t*_119.83_=−2.03; *P*=.04).

**Table 9:** Results of the linear mixed model analysis.

Model term	Estimate (SE)	*t* test (*df*)^a^	*P* value^b^
Intercept	63.38 (6.49)	9.77 (71.92)	<.001
Game phase	1.44 (0.49)	2.97 (120.12)	.004
Listening comprehension	1.56 (0.49)	3.17 (71.91)	.002
Receptive vocabulary	0.16 (0.06)	2.64 (72.27)	.01
Morphological awareness	0.23 (0.32)	0.73 (72.19)	.47
Game phase×listening comprehension	−0.08 (0.04)	−2.03 (119.83)	.04
Game phase×morphological awareness	−0.02 (0.02)	−0.60 (123.13)	.55
Game phase×receptive vocabulary	−0.01 (0.01)	−1.27 (123.71)	.21

^a^2-tailed.

^b^Significant at the .05 level.

## Discussion

### Principal Findings

This study addressed the enjoyment and feasibility of a story-listening game and preliminarily assessed the possible growth in language comprehension. The principal findings on game enjoyment, feasibility, and the impact of the game on language comprehension are discussed in the following sections.

Regarding the enjoyment evaluation, child and parental questionnaires and in-game enjoyment–related data (eg, story appreciation) pointed to a highly positive game experience. Indeed, questionnaires revealed high game enjoyment, motivation, and sustained attention during gameplay for most of the participants. The in-game data that were indicative of game enjoyment also showed that each story was appreciated by minimally three-quarters of the listeners, indicating that the stories fit the interests of kindergarteners aged 5 years with a cognitive risk for dyslexia and that most of the participants experienced the story game as enjoyable. Nevertheless, although a considerable number of GameFlow criteria were fully and partly implemented in the story game, probably benefiting the game enjoyment experience to a large extent, the GameFlow model–based evaluation also implied that there was room for optimizing the game design. Indeed, although the GameFlow model [5] was taken into account during the design process of the story game, the concentration principle remained the only principle for which all criteria were completely fulfilled.

Concerning the feasibility of the intervention in terms of difficulty, the results suggested that the game was adjusted to the cognitive capacities of children aged 5 years with an elevated risk for dyslexia, as almost all questions were answered correctly by minimally three-quarters of the listeners. This was not surprising, as we intended to develop a game that was not too easy or too difficult to ascertain that players were cognitively able to gather many coins but only if they actively listened to the stories. This state of active listening was confirmed by the general QRA results. The fact that only 11 questions were not answered correctly by 75% (62/82) of the listeners also suggests a rather stable difficulty level of the questions. As for feasibility in terms of intervention completion and duration, outcomes indicated a positive evaluation on the one hand, as most participants were able to fully or nearly complete the story game, but a negative evaluation on the other hand, as we observed a large variation in training duration (eg, days needed to nearly or completely finish the intervention). Thus, these findings mainly raised doubts about the feasibility of the training intensity, as many participants could finish but were simply not able to follow the rather highly concentrated advised schedule of 6 days per week during 12 weeks. This was also confirmed by the gaming profile outcomes, which revealed that only 22% (18/82) of participants belonged to the *complete and compliant group* and completed the game according to the postulated schedule compared with 39% (32/82) participants who managed to finish but spaced out the sessions over a longer time (eg, the complete and noncompliant group). As for predicting individuals’ final training duration and game exposure, we established a significantly negative relationship between initial mean story appreciation and final training duration and a positive relationship between initial mean QRA and final game exposure. These findings indicate that the initial compromised story appreciation decelerated the advised training scheme and that a higher response accuracy at the start increased the number of played sessions.

As for the growth in language comprehension, we established an increase in mean QRA along with the game phase, baseline vocabulary, and baseline listening comprehension. However, most strikingly, children with lower listening ability scores at the start of the intervention period made significantly more progress in terms of QRA than did children with higher preintervention listening ability scores. Overall, these results carefully suggest the potential of the game to train language comprehension with larger gains in children who already exhibit lower language levels at the start.

### Limitations and Comparisons to Prior Work

#### Enjoyment

The game enjoyment–related results give rise to 2 important discussion points. First, caution is required when interpreting the positive questionnaire outcomes. On the one hand, the phenomenon of social desirability (ie, the tendency to provide answers that correspond to widely accepted social norms) could have biased the responses of both children and parents [91]. On the other hand, the short attention span and underdeveloped cognitive capacities of young children could have resulted in extremely positive but less reliable responses on the smiley-o-meter. Indeed, in line with our study, Zaman et al [92] established an overrepresentation of positive responses when applying the smiley-o-meter in young children aged <7 years. Accordingly, in another study, the authors stated that children aged ≤7 years do not yet grasp the principles of a visual analog scale, of which the smiley-o-meter is an example [93]. As put forward by Zaman et al [92], the search for how to optimally measure the enjoyment of technology in preschoolers is still ongoing. The second discussion point related to enjoyment concerns the GameFlow-based evaluation. More specifically, we acknowledge that the agreement of the developed game with the GameFlow model contains a form of subjectivity, as the evaluation was performed by 2 members of the research group who were involved in the design process itself. Irrespective of this limitation, the fully and partly implemented GameFlow criteria embedded in the current game were presumably satisfactory enough to attain a certain level of enjoyment. This assumed enjoyment level was also supported by the cautiously interpreted questionnaire and in-game enjoyment–related outcomes, which indicated game appreciation in most players. Optimizing the partly or nonfulfilled criteria (eg, fixing the remaining bugs and clarifying the end goals) could increase the level of enjoyment even more. To deal with subjectivity, a future study could let adults who were not involved in the design process (eg, parents of playing children) rate the game.

#### Feasibility

This paragraph discusses 3 relevant points related to feasibility outcomes. First, although most of the participants (50/82, 61%) completed the intervention, we still acknowledge that 39% (32/82) participants dropped out at some point, mostly in the last game phases. This slightly corresponds to another home-based preventive intervention study in Dutch children at risk for dyslexia, which reported an attrition rate of 34% [94]. Unfortunately, the attrition rate of this paper still exceeded the desirable benchmark of 30% [95], and further research on why participants tended to drop out at some point is necessary. In a study by Justice et al [96], who investigated the feasibility, efficacy, and social validity of a 12-week home-based shared storybook reading intervention in preschoolers with language difficulties, the authors compared completers with noncompleters on a variety of child- and parent-specific characteristics and established risk factors of dropout, such as lower maternal age and lower parental educational levels. Distinguishing completers from noncompleters based on intrinsic and extrinsic features did not fall within the scope of this paper, albeit the research methods applied in the study by Justice et al [96] offer possibilities for future research that could be of added value to optimize the feasibility of the current intervention program. Irrespective of the rather high attrition rate, we still established a completion rate of 80% (approximately 57th session of the total 72 story sessions) in 90% (74/82) of the participants, suggesting that shortening the intervention by approximately 15 sessions, which almost equals 4 weeks in this paper, would result in lower dropout occurrence. However, an important sidenote relates to the fact that the participants in this paper combined the intervention with another tablet game (GG-FL or AC games). This could also have affected general motivation and perseverance. The second discussion point related to feasibility bears upon the variation in training intensity among the participants. As treatment fidelity in terms of quality and quantity has been found to predict learning outcomes in previous studies [94,97], the variation in training intensity cannot be neglected when investigating the educational impact of interest in future studies. However, in their reading intervention study, Katzir et al [98] found larger gains in sight word efficiency in a group of grade 1 to 3 children at risk for reading difficulties who received a 44-hour fluency-based therapy over a 9-month period compared with children who followed the exact same intervention within a more intensive period of 2 months. Hence, albeit the need for statistical confirmation in a future study, spacing out the story game intervention over time in this paper might have even benefited the expected educational learning outcomes. The last feasibility-related discussion point is linked to the prediction of the final game exposure and training duration based on story appreciation and QRA at the start of the intervention. The outcomes of these predictive analyses point to limitations in the current game design but give rise to suggestions for optimizing intervention feasibility in future studies. For example, given the importance of initial story appreciation to maintain the advised training intensity, a possible suggestion involves offering the storybook series in the preferred order of the participant. More specifically, providing a catalog menu in which players could choose which story series occur first might increase the story appreciation at the beginning and, as a result, the engagement to follow the advised schedule. Moreover, given the role of initial QRA in dropout occurrence, offering player-adapted questions based on an individual’s language knowledge and cognitive capacities, which then increase in difficulty, instead of fixed, predefined questions for all participants, might prevent participants from withdrawing from the study. A more interactive approach in which players could request and receive explanations of possible difficult words might also increase the chance of successfully responding to the questions from the start, lowering the chances of early dropout. However, a disadvantage of these proposed adjustments to prevent attrition relates to the fact that the growth of in-game QRA data then becomes less easily interpretable at the group level, as participants would follow an individually adapted trajectory. However, large-scale adaptability has been widely considered as one of the strengths of serious digital gaming interventions [4]. Furthermore, according to our GameFlow-based evaluation in its current form, the criterion of so-called *player-adjusted challenges* belonging to the overall challenge principle is lacking in the current story game. Although this principle was beyond the scope of the story game, its implementation could increase player enjoyment and decrease the attrition rate [5].

#### Growth in Language Comprehension

The results related to growth in language comprehension must be interpreted with extreme caution, and several important limitations should be elucidated. The first and most important limitation relates to the research design. In fact, the actual impact of the game on language comprehension can only be solidly established by conducting a randomized controlled trial (RCT), which was not performed in this study. Hence, although we found a larger growth in mean QRA in children with lower listening comprehension, the current research design does not allow us to draw univocal conclusions on the potential of the game to train language comprehension. Thus, a future RCT study, preferably including (1) a group that is purely playing the story game without combining it with other interventions such as GG-FL or AC games, (2) a no intervention control group, and (3) a control group receiving an alternative placebo treatment that does not specifically train language skills [99], is of crucial relevance to further disentangle the gaming effects on language comprehension. The second important limitation is related to the concept of content validity. We considered the in-game questions as a measure of language comprehension. However, despite the selection of age-appropriate stories based on library visits and the construction of the questions based on the vocabulary of the story content, the stories and questions did not belong to a validated comprehension test instrument, casting doubt on the certainty that we truly measured language comprehension in our participants [100]. In addition, we are unsure whether all questions and stories implemented in the game were of equal difficulty, although we found relatively stable response distributions for most of the questions. Hence, changes in performance might not be because of the intervention effects but simply because of the varying difficulty of the questions and stories. Conducting the pre- and postintervention measurements of independent language comprehension tests within an RCT design with appropriate control groups (as mentioned previously) would eliminate this concern. The third limitation discusses the distribution of the mean QRA in the first game phase, which already showed a tendency toward the ceiling. Hence, children with high accuracy scores from the start, which were related to higher baseline vocabulary and listening comprehension, did not have the potential to increase any further during the intervention, influencing the interpretation of the game phase–listening comprehension interaction. The high accuracy scores at the start of the study are presumably attributed to the fact that none of the participants in this paper experienced language difficulties. Indeed, originally, the story game was not developed to train language comprehension within the framework of the current research project but rather to investigate the potential of envelope-enhanced speech in children with a pure cognitive risk for dyslexia. Another factor that may influence the high initial accuracy scores relates to the type of posed questions. The questions intentionally only involved identifying or recalling items that were explicitly mentioned in the story text and thus examined language comprehension only at the literal language level [101]. Hence, questions at the inferential level, which, for example, require implying emotions, predictions, and connections between information within the text or with the child’s own experiences [102], were not part of the story game in its current form. For preschoolers, inferential questions are certainly more difficult to answer than literal ones [101]. However, given the scope of the story game within the current research project (ie, exposing children at risk for dyslexia, in a joyful way, to either envelope-enhanced or nonenhanced speech), questions were not supposed to be or become difficult but only served as a way of (1) ensuring active listening and (2) ascertaining that players earned as many coins as possible, increasing the chance of maintaining the intervention for 12 weeks. On the basis of these aforementioned limitations, the preliminary and cautiously interpreted results concerning a possible growth in language comprehension give rise to 3 important ideas for story game improvement if it would be used to train language comprehension at some point in populations with specific problems in this domain (eg, children with low socioeconomic status; bilingual children; and children with DLD, hearing impairment, or dyslexia). First, there is a clear need to include inferential questions, as Tarvainen et al [18] emphasized the importance of inferential language training in fostering language comprehension in preschoolers. Second, apart from optimizing the research design by conducting RCTs with adequate intervention and control groups, it is also of crucial relevance to include test batteries that assess comprehension at both the inferential and literal levels. The currently used CELF-4th edition, Dutch version [82] and Peabody Picture Vocabulary Test–III, Dutch version tasks [83] mainly focus on language comprehension at the literal level [101], emphasizing the need to add inferential language comprehension test batteries to the research protocol. Finally, researchers must take into account and tackle the possibility that children with lower language levels might show less motivation in storybook interventions than their typically developing peers [103].

### Conclusions

Overall, this newly developed story game seemed generally feasible and enjoyable for children aged 5 years with an elevated cognitive risk for developmental dyslexia. Moreover, our results preliminarily but carefully point to a potential for the game to train language comprehension. Hence, we hope to apply the story game in follow-up studies with an optimized study design in children who, for whatever reason, exhibit actual language comprehension difficulties. As such, we will be able to (1) generalize our enjoyment and feasibility findings and (2) draw solid conclusions on the actual influence of the game on language comprehension.

## Appendix

Multimedia Appendix 1 CONSORT-eHEALTH checklist (V 1.6.1).

Multimedia Appendix 2 Technical story game development guide.

Multimedia Appendix 3 Demonstration movie of the story game (© 2016 Clavis Publishing, Hasselt–Alkmaar, New York; illustrations and audiotext from *Lotta en de slodderkip* from Diane Put and Rik De Wulf).

Multimedia Appendix 4 Overview of the child and parental categorical questions and response possibilities.

## Abbreviations

AC: active control
CELF: Clinical Evaluation of Language Fundamentals
CONSORT-EHEALTH: Consolidated Standards of Reporting Trials of Electronic and Mobile Health Applications and Online Telehealth
DLD: developmental language disorder
EE: envelope enhancement
GG-FL: GraphoGame Flemish
KU Leuven: Katholieke Universiteit Leuven
QRA: question response accuracy
RCT: randomized controlled trial
