# Supplementary material for: Feasibility, Enjoyment, and Language Comprehension Impact of a Tablet- and GameFlow-Based Story-Listening Game for Kindergarteners: Methodological and Mixed Methods Study
Source: JMIR Serious Games. 2022 Mar 23;10(1):e34698. doi: 10.2196/34698 (PMC8987971; doi:10.2196/34698)
Supplement: Multimedia Appendix 1 [file games_v10i1e34698_app1.pdf]

CONSORT-EHEALTH (V 1.6.1) - Submission/Publication Form

The CONSORT-EHEALTH checklist is intended for authors of randomized trials evaluating web-based and Internet-based applications/interventions, including mobile interventions, electronic games (incl multiplayer games), social media, certain telehealth applications, and other interactive and/or networked electronic applications. Some of the items (e.g. all subitems under item 5 - description of the intervention) may also be applicable for other study designs.

The goal of the CONSORT EHEALTH checklist and guideline is to be
a) a guide for reporting for authors of RCTs,
b) to form a basis for appraisal of an ehealth trial (in terms of validity)

CONSORT-EHEALTH items/subitems are MANDATORY reporting items for studies published in the Journal of Medical Internet Research and other journals / scientific societies endorsing the checklist.

Items numbered 1., 2., 3., 4a., 4b etc are original CONSORT or CONSORT-NPT (non-pharmacologic treatment) items.
Items with Roman numerals (i., ii, iii, iv etc.) are CONSORT-EHEALTH extensions/clarifications.

As the CONSORT-EHEALTH checklist is still considered in a formative stage, we would ask that you also RATE ON A SCALE OF 1-5 how important/useful you feel each item is FOR THE PURPOSE OF THE CHECKLIST and reporting guideline (optional).

Mandatory reporting items are marked with a red *.
In the textboxes, either copy & paste the relevant sections from your manuscript into this form - please include any quotes from your manuscript in QUOTATION MARKS,
or answer directly by providing additional information not in the manuscript, or elaborating on why the item was not relevant for this study.

YOUR ANSWERS WILL BE PUBLISHED AS A SUPPLEMENTARY FILE TO YOUR PUBLICATION IN JMIR AND ARE CONSIDERED PART OF YOUR PUBLICATION (IF ACCEPTED).
Please fill in these questions diligently. Information will not be copyedited, so please use proper spelling and grammar, use correct capitalization, and avoid abbreviations.

DO NOT FORGET TO SAVE AS PDF _AND_ CLICK THE SUBMIT BUTTON SO YOUR ANSWERS ARE IN OUR DATABASE !!!

Citation Suggestion (if you append the pdf as Appendix we suggest to cite this paper in the caption):
Eysenbach G, CONSORT-EHEALTH Group
CONSORT-EHEALTH: Improving and Standardizing Evaluation Reports of Web-based and Mobile Health Interventions
J Med Internet Res 2011;13(4):e126
URL: http://www.jmir.org/2011/4/e126/

doi: 10.2196/jmir.1923
PMID: 22209829

femkevandenbempt@gmail.com (niet gedeeld) Ander account

Concept niet opgeslagen

*Vereist

Your name *

First Last

Vanden Bempt Femke

Primary Affiliation (short), City, Country *

University of Toronto, Toronto, Canada

KU Leuven

Your e-mail address *

abc@gmail.com

femke.vandenbempt@kuleuven.be

Title of your manuscript *

Provide the (draft) title of your manuscript.

Feasibility, enjoyment, and language comprehension impact of a tablet- and GameFlow-based story listening game for kindergarteners: a methodological and mixed method study

Name of your App/Software/Intervention *

If there is a short and a long/alternate name, write the short name first and add the long name in brackets.

Story game (it was called “Yellow star game” ir

Evaluated Version (if any)

e.g. "V1", "Release 2017-03-01", "Version 2.0.27913"

Jouw antwoord

Language(s) *

What language is the intervention/app in? If multiple languages are available, separate by comma (e.g. "English, French")

Dutch (Flemish accent, spoken in Belgium)

URL of your Intervention Website or App

e.g. a direct link to the mobile app on app in appstore (itunes, Google Play), or URL of the website. If the intervention is a DVD or hardware, you can also link to an Amazon page.

Jouw antwoord

URL of an image/screenshot (optional)

Jouw antwoord

Accessibility *

Can an enduser access the intervention presently?

- ○ access is free and open
- ○ access only for special usergroups, not open
- ○ access is open to everyone, but requires payment/subscription/in-app purchases
- ○ app/intervention no longer accessible
- ◉ Anders: Access was provided within the framework of the conducted study. I

Primary Medical Indication/Disease/Condition *

e.g. "Stress", "Diabetes", or define the target group in brackets after the condition, e.g. "Autism (Parents of children with)", "Alzheimers (Informal Caregivers of)"

Dyslexia (pre-readers at risk for dyslexia)

Primary Outcomes measured in trial *

comma-separated list of primary outcomes reported in the trial

The story game is enjoyable and feasible for tl

Secondary/other outcomes

Are there any other outcomes the intervention is expected to affect?

We preliminarily established a potential of the game to foster language comprehension, though not based on a randomized controlled trial design (in that sense we used the word 'preliminarily').

Recommended "Dose" *

What do the instructions for users say on how often the app should be used?

- ○ Approximately Daily
- ○ Approximately Weekly
- ○ Approximately Monthly
- ○ Approximately Yearly
- ○ "as needed"
- ◉ Anders: Approximately daily (e.g., six days per week for a period of twelve we

Approx. Percentage of Users (starters) still using the app as recommended after 3 months *

- ○ unknown / not evaluated
- ○ 0-10%
- ○ 11-20%
- ○ 21-30%
- ○ 31-40%
- ○ 41-50%
- ○ 51-60%
- ○ 61-70%
- ○ 71%-80%
- ○ 81-90%
- ○ 91-100%
- ◉ Anders: At the moment, it is not possible to use the application since it was c

Overall, was the app/intervention effective? *

- ◯ yes: all primary outcomes were significantly better in intervention group vs control
- ◯ partly: SOME primary outcomes were significantly better in intervention group vs control
- ◯ no statistically significant difference between control and intervention
- ◯ potentially harmful: control was significantly better than intervention in one or more outcomes
- ◯ inconclusive: more research is needed
- ◉ Anders: Inconclusive: more research (based on randomized controlled trials)

Article Preparation Status/Stage *

At which stage in your article preparation are you currently (at the time you fill in this form)

- ◯ not submitted yet - in early draft status
- ◯ not submitted yet - in late draft status, just before submission
- ◯ submitted to a journal but not reviewed yet
- ◯ submitted to a journal and after receiving initial reviewer comments
- ◉ submitted to a journal and accepted, but not published yet
- ◯ published
- ◯ Anders:

Journal *

If you already know where you will submit this paper (or if it is already submitted), please provide the journal name (if it is not JMIR, provide the journal name under "other")

- ( ) not submitted yet / unclear where I will submit this
- ( ) Journal of Medical Internet Research (JMIR)
- ( ) JMIR mHealth and UHealth
- (x) JMIR Serious Games
- ( ) JMIR Mental Health
- ( ) JMIR Public Health
- ( ) JMIR Formative Research
- ( ) Other JMIR sister journal
- ( ) Anders:

Is this a full powered effectiveness trial or a pilot/feasibility trial? *

- ( ) Pilot/feasibility
- (x) Fully powered

Manuscript tracking number *

If this is a JMIR submission, please provide the manuscript tracking number under "other" (The ms tracking number can be found in the submission acknowledgement email, or when you login as author in JMIR. If the paper is already published in JMIR, then the ms tracking number is the four-digit number at the end of the DOI, to be found at the bottom of each published article in JMIR)

- ( ) no ms number (yet) / not (yet) submitted to / published in JMIR
- (x) Anders: 34698

TITLE AND ABSTRACT

1a) TITLE: Identification as a randomized trial in the title

1a) Does your paper address CONSORT item 1a? *

I.e does the title contain the phrase "Randomized Controlled Trial"? (if not, explain the reason under "other")

- ○ yes
- ◉ Anders: This study was part of a broader reading intervention study (e.g., a q

1a-i) Identify the mode of delivery in the title

Identify the mode of delivery. Preferably use “web-based” and/or “mobile” and/or “electronic game” in the title. Avoid ambiguous terms like “online”, “virtual”, “interactive”. Use “Internet-based” only if Intervention includes non-web-based Internet components (e.g. email), use “computer-based” or “electronic” only if offline products are used. Use “virtual” only in the context of “virtual reality” (3-D worlds). Use “online” only in the context of “online support groups”. Complement or substitute product names with broader terms for the class of products (such as “mobile” or “smart phone” instead of “iphone”), especially if the application runs on different platforms.

	1	2	3	4	5	
subitem not at all important	○	○	○	◉	○	essential

Selectie wissen

Does your paper address subitem 1a-i? *

Copy and paste relevant sections from manuscript title (include quotes in quotation marks "like this" to indicate direct quotes from your manuscript), or elaborate on this item by providing additional information not in the ms, or briefly explain why the item is not applicable/relevant for your study

The mode of delivery was specified in the manuscript title as follows: “tablet and GameFlow-based story listening game”

1a-ii) Non-web-based components or important co-interventions in title

Mention non-web-based components or important co-interventions in title, if any (e.g., “with telephone support”).

	1	2	3	4	5	
subitem not at all important	○	○	◉	○	○	essential

Selectie wissen

Does your paper address subitem 1a-ii?

Copy and paste relevant sections from manuscript title (include quotes in quotation marks "like this" to indicate direct quotes from your manuscript), or elaborate on this item by providing additional information not in the ms, or briefly explain why the item is not applicable/relevant for your study

We specified the non-web-based components by describing the story game as a “tablet- and GameFlow-based story listening game”. Although all participants who played the story game combined it with either a tablet-based reading game (GraphoGame Flemish) or so-called active control games (Lego- and Duplo applications), we did not specify it in the manuscript title, since this was not relevant for the research questions addressed in the current study.

1a-iii) Primary condition or target group in the title

Mention primary condition or target group in the title, if any (e.g., “for children with Type I Diabetes”) Example: A Web-based and Mobile Intervention with Telephone Support for Children with Type I Diabetes: Randomized Controlled Trial

	1	2	3	4	5	
subitem not at all important	○	○	○	◉	○	essential

Selectie wissen

Does your paper address subitem 1a-iii? *

Copy and paste relevant sections from manuscript title (include quotes in quotation marks "like this" to indicate direct quotes from your manuscript), or elaborate on this item by providing additional information not in the ms, or briefly explain why the item is not applicable/relevant for your study

The target group was specified in the manuscript title as follows: “for kindergarteners”. In the current study, kindergarteners who played the game of interest were all at cognitive risk for dyslexia. However, we hope to conduct future studies in which we can test the feasibility of the game in kindergarteners without a risk for dyslexia (see subitem 21a-i).

1b) ABSTRACT: Structured summary of trial design, methods, results, and conclusions

NPT extension: Description of experimental treatment, comparator, care providers, centers, and blinding status.

1b-i) Key features/functionalities/components of the intervention and comparator in the METHODS section of the ABSTRACT

Mention key features/functionalities/components of the intervention and comparator in the abstract. If possible, also mention theories and principles used for designing the site. Keep in mind the needs of systematic reviewers and indexers by including important synonyms. (Note: Only report in the abstract what the main paper is reporting. If this information is missing from the main body of text, consider adding it)

	1	2	3	4	5	
subitem not at all important	○	○	○	◉	○	essential

Selectie wissen

Does your paper address subitem 1b-i? *

Copy and paste relevant sections from the manuscript abstract (include quotes in quotation marks "like this" to indicate direct quotes from your manuscript), or elaborate on this item by providing additional information not in the ms, or briefly explain why the item is not applicable/relevant for your study

The intervention of interest was specified in the abstract as follows: "a tablet-based serious story listening game for kindergarteners, developed based on the principles of the GameFlow model. The story game mainly involved story listening and rating, and responding to content-related questions."

1b-ii) Level of human involvement in the METHODS section of the ABSTRACT

Clarify the level of human involvement in the abstract, e.g., use phrases like “fully automated” vs. “therapist/nurse/care provider/physician-assisted” (mention number and expertise of providers involved, if any). (Note: Only report in the abstract what the main paper is reporting. If this information is missing from the main body of text, consider adding it)

	1	2	3	4	5	
subitem not at all important	○	○	○	●	○	essential

Selectie wissen

Does your paper address subitem 1b-ii?

Copy and paste relevant sections from the manuscript abstract (include quotes in quotation marks "like this" to indicate direct quotes from your manuscript), or elaborate on this item by providing additional information not in the ms, or briefly explain why the item is not applicable/relevant for your study

The level of human involvement was not specified in the manuscript, since children were instructed to play the story game with headphones (thus independently).

1b-iii) Open vs. closed, web-based (self-assessment) vs. face-to-face assessments in the METHODS section of the ABSTRACT

Mention how participants were recruited (online vs. offline), e.g., from an open access website or from a clinic or a closed online user group (closed usergroup trial), and clarify if this was a purely web-based trial, or there were face-to-face components (as part of the intervention or for assessment). Clearly say if outcomes were self-assessed through questionnaires (as common in web-based trials). Note: In traditional offline trials, an open trial (open-label trial) is a type of clinical trial in which both the researchers and participants know which treatment is being administered. To avoid confusion, use “blinded” or “unblinded” to indicated the level of blinding instead of “open”, as “open” in web-based trials usually refers to “open access” (i.e. participants can self-enrol). (Note: Only report in the abstract what the main paper is reporting. If this information is missing from the main body of text, consider adding it)

	1	2	3	4	5	
subitem not at all important	○	○	○	●	○	essential

Selectie wissen

Does your paper address subitem 1b-iii?

Copy and paste relevant sections from the manuscript abstract (include quotes in quotation marks "like this" to indicate direct quotes from your manuscript), or elaborate on this item by providing additional information not in the ms, or briefly explain why the item is not applicable/relevant for your study

The mode of recruitment was not described in the abstract in detail. Children for this particular study were recruited based on a large-scale screening which involved face-to-face and tablet-based assessments of non-verbal reasoning, phonological awareness, rapid automatized naming, and letter knowledge at school in a quiet test room. In order to exclude children that were born in other years than 2013, children with neurological, language, stuttering, articulation, or hearing problems, multilingual children, and children who had a schooling period of less than 24 months, parents filled out an online questionnaire per e-mail after they had signed an informed consent on which they provided their email addresses. In our manuscript, we refer to useful papers in which the screening procedure is fully described in detail.
Baseline language assessments (which we discuss in the current manuscript) took place at school in a quiet test room. Other relevant outcome measures of feasibility and enjoyment (e.g., story game appreciation and question response accuracy) were collected when the child played the story game at home. In-game data were sent daily to the research group via a University server. Next to the aforementioned baseline language assessments, the overall project, to which this study belonged to, also included baseline assessments of reading, phonology and general cognitive skills, as well as a post-intervention test (immediately after the intervention) and consolidation assessments (one and two years after the intervention period) of these skills. Pre-, post- and consolidation MRI and EEG measurements were also part of the project. However, given that this study aimed at investigating enjoyment, feasibility and the preliminary impact of the story game on language comprehension, the cognitive data at post- and consolidation phases as well as all MRI and EEG measurements were not discussed in the current study.

1b-iv) RESULTS section in abstract must contain use data

Report number of participants enrolled/assessed in each group, the use/uptake of the intervention (e.g., attrition/adherence metrics, use over time, number of logins etc.), in addition to primary/secondary outcomes. (Note: Only report in the abstract what the main paper is reporting. If this information is missing from the main body of text, consider adding it)

	1	2	3	4	5	
subitem not at all important	○	○	○	◉	○	essential

Selectie wissen

Does your paper address subitem 1b-iv?

Copy and paste relevant sections from the manuscript abstract (include quotes in quotation marks "like this" to indicate direct quotes from your manuscript), or elaborate on this item by providing additional information not in the ms, or briefly explain why the item is not applicable/relevant for your study

We stated the number of enrolled participants (in this case: the number of participants who were asked to play the game of interest of the study) and the number of participants for whom data was analyzed in the abstact as follows: “91 five-year old kindergarteners at cognitive risk for dyslexia were asked to play the story game for 12 weeks, six days per week, either combined with a tablet-based phonics intervention or active control games. Eventually, data from 82 participants were analyzed.” Moreover, attrition rate was also specified as follows given that this belonged to one of our indicators of feasibility: “Moreover, despite an overall attrition rate of 32 participants (39%), 74 participants (90%) still completed 80% of the game, albeit with a large

1b-v) CONCLUSIONS/DISCUSSION in abstract for negative trials

Conclusions/Discussions in abstract for negative trials: Discuss the primary outcome - if the trial is negative (primary outcome not changed), and the intervention was not used, discuss whether negative results are attributable to lack of uptake and discuss reasons. (Note: Only report in the abstract what the main paper is reporting. If this information is missing from the main body of text, consider adding it)

	1	2	3	4	5	
subitem not at all important	○	○	○	◉	○	essential

Selectie wissen

Does your paper address subitem 1b-v?

Copy and paste relevant sections from the manuscript abstract (include quotes in quotation marks "like this" to indicate direct quotes from your manuscript), or elaborate on this item by providing additional information not in the ms, or briefly explain why the item is not applicable/relevant for your study

This is not applicable for the current study.

INTRODUCTION

2a) In INTRODUCTION: Scientific background and explanation of rationale

2a-i) Problem and the type of system/solution

Describe the problem and the type of system/solution that is object of the study: intended as stand-alone intervention vs. incorporated in broader health care program? Intended for a particular patient population? Goals of the intervention, e.g., being more cost-effective to other interventions, replace or complement other solutions? (Note: Details about the intervention are provided in “Methods” under 5)

	1	2	3	4	5	
subitem not at all important	○	○	○	○	○	essential

Does your paper address subitem 2a-i? *

Copy and paste relevant sections from the manuscript (include quotes in quotation marks "like this" to indicate direct quotes from your manuscript), or elaborate on this item by providing additional information not in the ms, or briefly explain why the item is not applicable/relevant for your study

The problem statement is specified in the manuscript as follows: “Given the evidence of storybook listening to foster several language comprehension components in young children, e.g., receptive vocabulary, morphosyntax, and narrative comprehension, both in physical or digital settings, to the best of our knowledge, no study has ever embedded the story listening method in a serious gaming context. However, when designing a commercial and/or serious digital game, a detailed knowledge of game enjoyment is of crucial relevance. A digital game that is not experienced as fun, will not be played and consequently, in the case of serious gaming, learning will not occur. Hence, when evaluating serious games, both aspects of enjoyment as well as educational impact must be considered.”
The original goal and target group for which the intervention was created was specified in the introduction as follows: “The story game was originally developed to enhance basic auditory speech processing by modifying the recorded speech signals of the stories with a so-called envelope enhancement (EE) algorithm and as such, boost phonology and reading in kindergarteners at cognitive risk for dyslexia.” However, we want to emphasize in this questionnaire that the current study did not intend to investigate this specific effect of auditory speech processing on phonology and reading. As such, the current study does technically not describe a randomized controlled design, as in the current study, all groups who played the story game (the game of interest) were pooled together.

2a-ii) Scientific background, rationale: What is known about the (type of) system

Scientific background, rationale: What is known about the (type of) system that is the object of the study (be sure to discuss the use of similar systems for other conditions/diagnoses, if appropiate), motivation for the study, i.e. what are the reasons for and what is the context for this specific study, from which stakeholder viewpoint is the study performed, potential impact of findings [2]. Briefly justify the choice of the comparator.

	1	2	3	4	5	
subitem not at all important	○	○	○	○	○	essential

Does your paper address subitem 2a-ii? *

Copy and paste relevant sections from the manuscript (include quotes in quotation marks "like this" to indicate direct quotes from your manuscript), or elaborate on this item by providing additional information not in the ms, or briefly explain why the item is not applicable/relevant for your study

Relevant information about the type of system and the rationale to implement a story game in a gaming context is specified in the manuscript as follows: “In young children under the age of six, implicit therapy techniques, in which the child is exposed to optimal language without explicitly explaining certain rules, are preferred over explicit instructional approaches. An example of a rather implicit approach concerns the method of storybook listening. Given the evidence of storybook listening to foster several language comprehension components in young children, e.g., receptive vocabulary, morphosyntax, and narrative comprehension, both in physical or digital settings, to the best of our knowledge, no study has ever embedded the story listening method in a serious gaming context. Often, digital games offer stimulating audio-visual game worlds and appealing rewards. Given these motivational aspects, serious gaming is already widely applied in educational, psychological, and medical contexts, including the field of language learning (e.g., game-based interventions specifically targeting

2b) In INTRODUCTION: Specific objectives or hypotheses

Does your paper address CONSORT subitem 2b? *

Copy and paste relevant sections from the manuscript (include quotes in quotation marks "like this" to indicate direct quotes from your manuscript), or elaborate on this item by providing additional information not in the ms, or briefly explain why the item is not applicable/relevant for your study

The specific objectives are described in the manuscript as follows: “The current research article will address enjoyment and feasibility of a newly developed serious tablet- and game-based story listening intervention (henceforth story game), for which the GameFlow model had served as a guideline in the design process. Moreover, given the focus of the game on story listening, which is a frequently used method to increase young children’s language comprehension, its potential to foster language comprehension will also be preliminarily investigated.”

METHODS

3a) Description of trial design (such as parallel, factorial) including allocation ratio

Does your paper address CONSORT subitem 3a? *

Copy and paste relevant sections from the manuscript (include quotes in quotation marks "like this" to indicate direct quotes from your manuscript), or elaborate on this item by providing additional information not in the ms, or briefly explain why the item is not applicable/relevant for your study

Participants of the current study were part of a large-scale preventive reading intervention study. However, different experimental groups of the reading intervention study were pooled together to tackle the research questions stated in the current manuscript. We therefore described the study design in the current manuscript as follows: “Of the entire intervention study sample (n=149), 91 at-risk children were asked to play the story game. Within the framework of the reading intervention study, these 91 children were randomly assigned to one of three experimental groups. The first group (GGFL_EE group, n=31) played the story game with envelope enhanced stories and combined it with the phonics-based GG-FL intervention. The second group received the same intervention as the first with the only difference that there was no envelope enhancement applied to the stories in the story game (GGFL_NE group, n=31). The third group also played the non-enhanced story game and combined it with tablet-based commercial Lego- and Duplo-applications (henceforth active control (AC) games), which did not train any reading-related skills (AC_NE group, n=29). The remaining 28 at-risk and 30 non-at-risk children of the total sample served as the at-risk passive control and typically developing control group of the reading intervention study respectively and did not receive any type of digital gaming intervention. Since the current study mainly focuses on the story game feasibility and enjoyment, these last two control groups, who did not play the story game, will not be discussed any further in the current research article.”

3b) Important changes to methods after trial commencement (such as eligibility criteria), with reasons

Does your paper address CONSORT subitem 3b? *

Copy and paste relevant sections from the manuscript (include quotes in quotation marks "like this" to indicate direct quotes from your manuscript), or elaborate on this item by providing additional information not in the ms, or briefly explain why the item is not applicable/relevant for your study

We did not make important changes to the methods after trial commencement.

3b-i) Bug fixes, Downtimes, Content Changes

Bug fixes, Downtimes, Content Changes: ehealth systems are often dynamic systems. A description of changes to methods therefore also includes important changes made on the intervention or comparator during the trial (e.g., major bug fixes or changes in the functionality or content) (5-iii) and other “unexpected events” that may have influenced study design such as staff changes, system failures/downtimes, etc. [2].

	1	2	3	4	5	
subitem not at all important	○	○	○	◉	○	essential

Selectie wissen

Does your paper address subitem 3b-i?

Copy and paste relevant sections from the manuscript (include quotes in quotation marks "like this" to indicate direct quotes from your manuscript), or elaborate on this item by providing additional information not in the ms, or briefly explain why the item is not applicable/relevant for your study

We did not change the content of the story game described in the study during the intervention period. However, bugs, resulting in a total crash of the game and loss of data, were apparent in a subsample of our participants (n=7). This resulted in home visits where the tablet-game was reinstalled by a member of the research group so that participants could continue with the intervention. At the time of the intervention, the bug was thus not fixed in the source code of the game, but we acknowledged in Multimedia Appendix 1 that this is of major importance when the game would be used in future studies: “When applying the game in future studies, fixing these bugs must take the highest priority.”

4a) Eligibility criteria for participants

Does your paper address CONSORT subitem 4a? *

Copy and paste relevant sections from the manuscript (include quotes in quotation marks "like this" to indicate direct quotes from your manuscript), or elaborate on this item by providing additional information not in the ms, or briefly explain why the item is not applicable/relevant for your study

Eligibility criteria were described in the manuscript as follows: “Following a school-based screening in the third year of kindergarten (n=1225), 149 five-year old children (119 children with and 30 children without an elevated cognitive risk for dyslexia) enrolled in a game-based preventive reading intervention study (Trial registration number: S60962 – assigned by the Clinical Trial Center of UZ Leuven, Belgium). A cognitive risk for dyslexia was assigned when a child scored above the 10th percentile on a non-verbal reasoning test and below percentile 30 on minimally two out of three assessments of robust reading predictors, i.e., letter knowledge, phonological awareness, and rapid automatized naming. Typically developing children scored above percentile 40 on all reading precursors and were matched to the risk-sample based on non-verbal reasoning ability, school environment, and gender. All selected participants were in their third year of kindergarten, Flemish monolingual Dutch-speaking, born in 2013, and had a schooling period of minimally 20 months. None of the selected children reported an additional behavioral or familial risk for ADHD, language and/or articulatory problems, a severe hearing impairment, or neurological deficits.”

4a-i) Computer / Internet literacy

Computer / Internet literacy is often an implicit “de facto” eligibility criterion - this should be explicitly clarified.

	1	2	3	4	5	
subitem not at all important	○	○	○	◉	○	essential

Selectie wissen

Does your paper address subitem 4a-i?

Copy and paste relevant sections from the manuscript (include quotes in quotation marks "like this" to indicate direct quotes from your manuscript), or elaborate on this item by providing additional information not in the ms, or briefly explain why the item is not applicable/relevant for your study

Although relevant, we did not obtain information about computer literacy in our participants. However, given the rising exposure to technological devices in children’s current daily lives and the clarity on how to use the game mechanics of the story game described in the study, we believe that computer literacy has not such a major impact on the results.

4a-ii) Open vs. closed, web-based vs. face-to-face assessments:

Open vs. closed, web-based vs. face-to-face assessments: Mention how participants were recruited (online vs. offline), e.g., from an open access website or from a clinic, and clarify if this was a purely web-based trial, or there were face-to-face components (as part of the intervention or for assessment), i.e., to what degree got the study team to know the participant. In online-only trials, clarify if participants were quasi-anonymous and whether having multiple identities was possible or whether technical or logistical measures (e.g., cookies, email confirmation, phone calls) were used to detect/prevent these.

	1	2	3	4	5	
subitem not at all important	○	○	○	●	○	essential

Selectie wissen

Does your paper address subitem 4a-ii? *

Copy and paste relevant sections from the manuscript (include quotes in quotation marks "like this" to indicate direct quotes from your manuscript), or elaborate on this item by providing additional information not in the ms, or briefly explain why the item is not applicable/relevant for your study

Children in the current study were recruited following a large-scale screening research involving 1225 children with face to face and tablet-based assessments of non-verbal reasoning, letter knowledge, rapid naming and phonological awareness at school. All children that participated in the screening research were recruited by distributing information letters and informed consent letters to more than 700 Flemish schools. Parents who gave their e-mail addresses and signed the informed consent received a questionnaire in order to prevent children with hearing impairment, neurological problems, language, articulation, and stuttering problems, children with a schooling period of less than 24 months, and children with a birth year other than 2013 from participation to the screening. Children that eventually participated in the preventive reading intervention study (selected based on the screening results) were tested at school on a variety of cognitive tasks related to reading, cognition and language, before and after and one and two years after the intervention. However, given the objectives of the current study, post-and consolidation-test measurements were not discussed in the manuscript. The intervention itself was home- and tablet-based and intervention-related data were sent daily to a university server via internet.

4a-iii) Information giving during recruitment

Information given during recruitment. Specify how participants were briefed for recruitment and in the informed consent procedures (e.g., publish the informed consent documentation as appendix, see also item X26), as this information may have an effect on user self-selection, user expectation and may also bias results.

	1	2	3	4	5	
subitem not at all important	○	○	○	○	○	essential

Does your paper address subitem 4a-iii?

Copy and paste relevant sections from the manuscript (include quotes in quotation marks "like this" to indicate direct quotes from your manuscript), or elaborate on this item by providing additional information not in the ms, or briefly explain why the item is not applicable/relevant for your study

Participants were briefed based on an extensive informed consent letter, which was approved by the ethical medical committee of UZ Leuven, Belgium.

4b) Settings and locations where the data were collected

Does your paper address CONSORT subitem 4b? *

Copy and paste relevant sections from the manuscript (include quotes in quotation marks "like this" to indicate direct quotes from your manuscript), or elaborate on this item by providing additional information not in the ms, or briefly explain why the item is not applicable/relevant for your study

The setting and location of the data collection was described in the manuscript as follows: “A variety of cognitive-linguistic tasks, including baseline language skills (listening comprehension, receptive vocabulary, and morphological awareness) were individually assessed in all participants at school in a quiet test room. After the intervention period, parents independently filled out a short questionnaire including three questions related to motivation, encouragement, and sustained attention during story gameplay. Accompanied by a member of the research group at school, intervention enjoyment was also measured in all children using two components of the Fun Toolkit survey instrument, which was developed to gather children’s opinions on technology.” Parents filled out their questionnaire in the university hospital of KU Leuven (UZ Leuven) when they came to the lab with their child for the pre-test MRI session. More detailed information on the procedure of the listening comprehension, receptive vocabulary, and morphological awareness task is also fully described in the manuscript.

4b-i) Report if outcomes were (self-)assessed through online questionnaires

Clearly report if outcomes were (self-)assessed through online questionnaires (as common in web-based trials) or otherwise.

	1	2	3	4	5	
subitem not at all important	○	○	◉	○	○	essential

Selectie wissen

Does your paper address subitem 4b-i? *

Copy and paste relevant sections from the manuscript (include quotes in quotation marks "like this" to indicate direct quotes from your manuscript), or elaborate on this item by providing additional information not in the ms, or briefly explain why the item is not applicable/relevant for your study

Outcomes relevant for the manuscript were not assessed through online questionnaires since participants were five-year old children at cognitive risk for dyslexia, and thus too young for this procedure. The mode of assessment was stated in the manuscript as follows: “A variety of cognitive-linguistic tasks, including baseline language skills (listening comprehension, receptive vocabulary, and morphological awareness) were individually assessed in all participants at school in a quiet test room. After the intervention period, parents independently filled out a short questionnaire including three questions related to motivation, encouragement, and sustained attention during story gameplay. Accompanied by a member of the research group at school, intervention enjoyment was also measured in all children using two components of the Fun Toolkit survey instrument, which was developed to gather children’s opinions on technology.” Intervention-related data (such as game progress, question response accuracy) was collected while the participants were playing the story game at home. Useful data were sent daily to the research group via a University server. This process was stated in the manuscript as follows: “Useful player data of individual player accounts (e.g., game progress, question response accuracy, play dates and hours, and story rating information) were automatically logged on our university server and sent daily to the research group.”

4b-ii) Report how institutional affiliations are displayed

Report how institutional affiliations are displayed to potential participants [on ehealth media], as affiliations with prestigious hospitals or universities may affect volunteer rates, use, and reactions with regards to an intervention.(Not a required item – describe only if this may bias results)

	1	2	3	4	5	
subitem not at all important	○	○	○	○	○	essential

Does your paper address subitem 4b-ii?

Copy and paste relevant sections from the manuscript (include quotes in quotation marks "like this" to indicate direct quotes from your manuscript), or elaborate on this item by providing additional information not in the ms, or briefly explain why the item is not applicable/relevant for your study

Jouw antwoord

5) The interventions for each group with sufficient details to allow replication, including how and when they were actually administered

5-i) Mention names, credential, affiliations of the developers, sponsors, and owners

Mention names, credential, affiliations of the developers, sponsors, and owners [6] (if authors/evaluators are owners or developer of the software, this needs to be declared in a “Conflict of interest” section or mentioned elsewhere in the manuscript).

	1	2	3	4	5	
subitem not at all important	○	○	●	○	○	essential

Selectie wissen

Does your paper address subitem 5-i?

Copy and paste relevant sections from the manuscript (include quotes in quotation marks "like this" to indicate direct quotes from your manuscript), or elaborate on this item by providing additional information not in the ms, or briefly explain why the item is not applicable/relevant for your study

The application (henceforth story game) was developed entirely within the KU Leuven without external partners and/or sponsors. Multimedia Appendix 1 of the manuscript provides a detailed explanation of how the game was programmed so that other researchers can create an application with the same programming structure.

5-ii) Describe the history/development process

Describe the history/development process of the application and previous formative evaluations (e.g., focus groups, usability testing), as these will have an impact on adoption/use rates and help with interpreting results.

	1	2	3	4	5	
subitem not at all important	○	○	○	○	○	essential

Does your paper address subitem 5-ii?

Copy and paste relevant sections from the manuscript (include quotes in quotation marks "like this" to indicate direct quotes from your manuscript), or elaborate on this item by providing additional information not in the ms, or briefly explain why the item is not applicable/relevant for your study

A detailed overview of the development process of the story game is provided in Multimedia appendix 1. Before the start of the current study, the game had been tested in a pilot study involving around 20 five-year old children. However, the final version of the game was used for the first time with the children that participated in the current intervention study. Given that this was the first time the application was used in its final form, we evaluated feasibility and enjoyment in the current study in order to hopefully implement the game in future studies among other populations than children at cognitive risk for dyslexia and conduct randomized controlled trials in order to solidly investigate the impact of the game on language comprehension.

5-iii) Revisions and updating

Revisions and updating. Clearly mention the date and/or version number of the application/intervention (and comparator, if applicable) evaluated, or describe whether the intervention underwent major changes during the evaluation process, or whether the development and/or content was “frozen” during the trial. Describe dynamic components such as news feeds or changing content which may have an impact on the replicability of the intervention (for unexpected events see item 3b).

	1	2	3	4	5	
subitem not at all important	○	○	●	○	○	essential

Selectie wissen

Does your paper address subitem 5-iii?

Copy and paste relevant sections from the manuscript (include quotes in quotation marks "like this" to indicate direct quotes from your manuscript), or elaborate on this item by providing additional information not in the ms, or briefly explain why the item is not applicable/relevant for your study

The intervention did not undergo major changes during the intervention period. This is the first (final) version of the game, since it was developed entirely within the KU Leuven and only within the framework of the reading intervention study.

5-iv) Quality assurance methods

Provide information on quality assurance methods to ensure accuracy and quality of information provided [1], if applicable.

	1	2	3	4	5	
subitem not at all important	○	○	○	◉	○	essential

Selectie wissen

Does your paper address subitem 5-iv?

Copy and paste relevant sections from the manuscript (include quotes in quotation marks "like this" to indicate direct quotes from your manuscript), or elaborate on this item by providing additional information not in the ms, or briefly explain why the item is not applicable/relevant for your study

In-game intervention data were logged and sent daily to the research group via a university server in order to prevent the loss of useful intervention data. This data logging was also useful to check if participants continued playing according to the schedule. If participants did not follow the advised training scheme, a member of the research group contacted them (by e-mail or phone) to encourage them to continue

5-v) Ensure replicability by publishing the source code, and/or providing screenshots/screen-capture video, and/or providing flowcharts of the algorithms used

Ensure replicability by publishing the source code, and/or providing screenshots/screen-capture video, and/or providing flowcharts of the algorithms used. Replicability (i.e., other researchers should in principle be able to replicate the study) is a hallmark of scientific reporting.

	1	2	3	4	5	
subitem not at all important	○	○	◉	○	○	essential

Selectie wissen

Does your paper address subitem 5-v?

Copy and paste relevant sections from the manuscript (include quotes in quotation marks "like this" to indicate direct quotes from your manuscript), or elaborate on this item by providing additional information not in the ms, or briefly explain why the item is not applicable/relevant for your study

Multimedia Appendix 1 contains a detailed overview of the development process and the rationale behind all developmental decisions. Moreover, Supplementary Table 2 provides an overview and explanation of all used scripts implemented in the game. Specific design and programming details are also provided in Multimedia Appendix 1, so that external researchers can understand the code if necessary. Both in the manuscript as well as in Multimedia Appendix 1, screenshots of the game are provided.

5-vi) Digital preservation

Digital preservation: Provide the URL of the application, but as the intervention is likely to change or disappear over the course of the years; also make sure the intervention is archived (Internet Archive, webcitation.org, and/or publishing the source code or screenshots/videos alongside the article). As pages behind login screens cannot be archived, consider creating demo pages which are accessible without login.

	1	2	3	4	5	
subitem not at all important	○	○	○	○	○	essential

Does your paper address subitem 5-vi?

Copy and paste relevant sections from the manuscript (include quotes in quotation marks "like this" to indicate direct quotes from your manuscript), or elaborate on this item by providing additional information not in the ms, or briefly explain why the item is not applicable/relevant for your study

For the moment, copy rights of the stories and images used in the story game were only obtained for the purpose of the reading intervention study. Thus, the application is not (yet) online available nor commercially available via app stores. Therefore, providing URLs of the application is currently impossible.

5-vii) Access

Access: Describe how participants accessed the application, in what setting/context, if they had to pay (or were paid) or not, whether they had to be a member of specific group. If known, describe how participants obtained “access to the platform and Internet” [1]. To ensure access for editors/reviewers/readers, consider to provide a “backdoor” login account or demo mode for reviewers/readers to explore the application (also important for archiving purposes, see vi).

	1	2	3	4	5	
subitem not at all important	○	○	○	◉	○	essential

Selectie wissen

Does your paper address subitem 5-vii? *

Copy and paste relevant sections from the manuscript (include quotes in quotation marks "like this" to indicate direct quotes from your manuscript), or elaborate on this item by providing additional information not in the ms, or briefly explain why the item is not applicable/relevant for your study

In total, 91 children were assigned to experimental intervention groups which involved playing the story game (see description of the study design – subitem 3a). After the random allocation process, but before handing out tablets to these 91 participants, members of the research group created player accounts for each participant. When opening the game for the first time, participants saw a game avatar (either a boy or a girl depending on the gender of the participant) and their name. Further access to the game was also self-explanatory. Given the copyrights, participants and their parents were not able to create extra player accounts and the game settings, which allowed creating extra accounts, were locked with a password.

5-viii) Mode of delivery, features/functionalities/components of the intervention and comparator, and the theoretical framework

Describe mode of delivery, features/functionalities/components of the intervention and comparator, and the theoretical framework [6] used to design them (instructional strategy [1], behaviour change techniques, persuasive features, etc., see e.g., [7, 8] for terminology). This includes an in-depth description of the content (including where it is coming from and who developed it) [1]," whether [and how] it is tailored to individual circumstances and allows users to track their progress and receive feedback" [6]. This also includes a description of communication delivery channels and – if computer-mediated communication is a component – whether communication was synchronous or asynchronous [6]. It also includes information on presentation strategies [1], including page design principles, average amount of text on pages, presence of hyperlinks to other resources, etc. [1].

	1	2	3	4	5	
subitem not at all important	○	○	○	◉	○	essential

Selectie wissen

Does your paper address subitem 5-viii? *

Copy and paste relevant sections from the manuscript (include quotes in quotation marks "like this" to indicate direct quotes from your manuscript), or elaborate on this item by providing additional information not in the ms, or briefly explain why the item is not applicable/relevant for your study

The mode of delivery, features/functionalities/compontens of the game were described in the manusript as follows: “After this baseline test-phase, the 91 at-risk children who were assigned to one of the three intervention groups received a tablet, its corresponding calibrated headphone, and a manual for parents with instructions to start the tablet intervention at home. With support of a reward calendar with stickers, they were instructed to combine story game sessions with 15-minute GG-FL/AC sessions for six days per week over a period of 12 weeks.”

Components of the intervention were described as follows: “In total, 87 stories from 14 different book series were implemented in the game. Generally, the game contained three main game modes, i.e., (1) the main intervention task environment, which involved the actual story listening, a story rating, and responding to content-related questions (2) the virtual hub world, in which players consulted their game progress on a map, and (3) an avatar customization system, where players could buy accessories for their game avatars or buy new avatars. Most of the artwork in the game, such as 3D models, textures and animations which were used to create the virtual hub world, the avatars, and their accessories, was either custom-made, acquired from the Unity Asset Store, or acquired from child-friendly projects of the Dyslexia Research Collaboration (DYSCO) team at KU Leuven, such as Diesel-X. The resulting art style was characterized by flat primary colors, avatars with exaggerated cartoon-like proportions, and simplified basic models and animations, suitable for the chosen target age group.”

Since participants of the current study were pooled together and all played the story game, a comparator game is not described and not available. The theoretical framework is provided in the manuscript as follows: “When designing a commercial and/or serious digital game, a detailed knowledge of game enjoyment is of crucial relevance. A digital game that is not experienced as fun, will not be played and consequently, in the case of serious gaming, learning will not occur. Hence, when evaluating serious games, both aspects of enjoyment as well as educational impact must be considered. Yet, despite its high relevance, the factor of enjoyment is hardly ever evaluated in existing serious games due to the lack of proper frameworks. To both clarify the concept of game enjoyment as well as to facilitate its evaluation, Sweetser and Wyeth proposed a summarizing framework, i.e., the GameFlow model, which is based on the extensive gaming literature and the general theory of Flow. The model intends to guide serious game developers in the process of creating and improving games that are both educational and enjoyable. More specifically, the GameFlow model proposes eight interrelated gaming elements, which are important to attain overall game enjoyment: (1) concentration, (2) challenge, (3) player skills, (4) control, (5) clear end or intermediate goals, (6) feedback, (7) immersion, and (8) social interaction. The current research article will address enjoyment and feasibility of a newly developed serious tablet- and game-based story listening intervention (henceforth story game), for which the GameFlow model had served as a guideline in the design process.”

5-ix) Describe use parameters

Describe use parameters (e.g., intended “doses” and optimal timing for use). Clarify what instructions or recommendations were given to the user, e.g., regarding timing, frequency, heaviness of use, if any, or was the intervention used ad libitum.

	1	2	3	4	5	
subitem not at all important	○	○	○	◉	○	essential

Selectie wissen

Does your paper address subitem 5-ix?

Copy and paste relevant sections from the manuscript (include quotes in quotation marks "like this" to indicate direct quotes from your manuscript), or elaborate on this item by providing additional information not in the ms, or briefly explain why the item is not applicable/relevant for your study

Instructions to the participants were described in the manuscript as follows: “After this baseline test-phase, the 91 at-risk children who were assigned to one of the three intervention groups received a tablet, its corresponding calibrated headphone, and a manual for parents with instructions to start the tablet intervention at home. With support of a reward calendar with stickers, they were instructed to combine story game sessions with 15-minute GG-FL/AC sessions for six days per week over a period of 12 weeks. This equaled a total of 72 gaming sessions, both for GG-FL/AC as well as for the story game.”

5-x) Clarify the level of human involvement

Clarify the level of human involvement (care providers or health professionals, also technical assistance) in the e-intervention or as co-intervention (detail number and expertise of professionals involved, if any, as well as “type of assistance offered, the timing and frequency of the support, how it is initiated, and the medium by which the assistance is delivered”. It may be necessary to distinguish between the level of human involvement required for the trial, and the level of human involvement required for a routine application outside of a RCT setting (discuss under item 21 – generalizability).

	1	2	3	4	5	
subitem not at all important	○	○	○	◉	○	essential

Selectie wissen

Does your paper address subitem 5-x?

Copy and paste relevant sections from the manuscript (include quotes in quotation marks "like this" to indicate direct quotes from your manuscript), or elaborate on this item by providing additional information not in the ms, or briefly explain why the item is not applicable/relevant for your study

In the intervention manual for parents, it was clearly emphasized that children should play the story game exclusively with a headphone (since all sound levels of the stories were calibrated with ATH m20x headphones). That way, it was obvious that the level of human involvement was set to a minimum/zero since parents/caregivers could not hear the stories the participants had to listen to. In the post-intervention questionnaires, parents did sometimes indicate that they helped their child with practical aspects during the first intervention session (e.g., starting the application, setting up the game session for the first time), but this was only the case for a miniority of participants.

5-xi) Report any prompts/reminders used

Report any prompts/reminders used: Clarify if there were prompts (letters, emails, phone calls, SMS) to use the application, what triggered them, frequency etc. It may be necessary to distinguish between the level of prompts/reminders required for the trial, and the level of prompts/reminders for a routine application outside of a RCT setting (discuss under item 21 – generalizability).

	1	2	3	4	5	
subitem not at all important	○	○	○	●	○	essential

Selectie wissen

Does your paper address subitem 5-xi? *

Copy and paste relevant sections from the manuscript (include quotes in quotation marks "like this" to indicate direct quotes from your manuscript), or elaborate on this item by providing additional information not in the ms, or briefly explain why the item is not applicable/relevant for your study

As stated in item 5-ix, data were logged and sent daily to a university server. When participants did not play according to the advised training schedule, they received a phone call/e-mail from a member of the research group in order to encourage them to continue playing.

5-xii) Describe any co-interventions (incl. training/support)

Describe any co-interventions (incl. training/support): Clearly state any interventions that are provided in addition to the targeted eHealth intervention, as ehealth intervention may not be designed as stand-alone intervention. This includes training sessions and support [1]. It may be necessary to distinguish between the level of training required for the trial, and the level of training for a routine application outside of a RCT setting (discuss under item 21 – generalizability.

	1	2	3	4	5	
subitem not at all important	○	○	○	◉	○	essential

Selectie wissen

Does your paper address subitem 5-xii? *

Copy and paste relevant sections from the manuscript (include quotes in quotation marks "like this" to indicate direct quotes from your manuscript), or elaborate on this item by providing additional information not in the ms, or briefly explain why the item is not applicable/relevant for your study

In the current study, all participants who played the story game combined it with either a Flemish tablet-based reading intervention (GraphoGame Flemish) or active control games (e.g., Lego and Duplo games). The original purpose of the story game (not investigated in the current study) was to induce a boosting effect of the envelope enhancement algorithm of the stories on phonology and reading on top of GraphoGame Flemish. This boosting effect was not investigated in the current study, but will be part of a future study. Thus, in this particular project, the story game intervention was not designed as a stand-alone intervention. However, we hope to use it in the future as a stand-alone intervention in studies to investigate the actual impact of the story game on language comprehension.

6a) Completely defined pre-specified primary and secondary outcome measures, including how and when they were assessed

Does your paper address CONSORT subitem 6a? *

Copy and paste relevant sections from the manuscript (include quotes in quotation marks "like this" to indicate direct quotes from your manuscript), or elaborate on this item by providing additional information not in the ms, or briefly explain why the item is not applicable/relevant for your study

Assessment of primary and secondary outcomes were described in the manuscript as follows: “Story game enjoyment was addressed in three ways. First, post-intervention child and parental questionnaire outcomes were analyzed by visualizing the relative frequencies per response category of each question. Second, two members of the research group independently evaluated the GameFlow criteria by assigning a state of either fulfilled, partly fulfilled, or not fulfilled. Then, after reaching a consensus, all criteria received a final single state. Third, in-game enjoyment-related data were evaluated, such as general story appreciation, measured as the proportion of given likes and dislikes per story. In order to gain insights into the feasibility of the intervention, we first defined the general question response accuracy, which indicated the difficulty level and thus the feasibility of the story listening part of the game. It was computed as the proportion of correct and incorrect responses per question. Second, we visualized the attrition rate, which determined how many children completed the intervention and at what point eventual drop-outs occurred. Third, we calculated the individuals’ final game exposure and training duration. Final game exposure was computed as the ratio (%) of actually played sessions and the total available sessions (i.e., 72). Individuals’ corresponding final training duration represented the amount of days the child played, starting from the day of the first story session until the day on which the participant played for the last time, irrespective of game completion. The measure of final training duration was particularly informative on the feasibility of the training intensity. By analyzing players’ final game exposure and training duration, we categorized players into different gaming profiles based on intervention completion (complete/incomplete) and schedule compliance (compliant/non-compliant). Four additional non-parametric median-based Theil-Sen regressions were performed using the ‘mblm’ package in R to predict either individuals’ final game exposure or final training duration, either based on initial individual story appreciation or question response accuracy. These analyses provide information on how to keep players engaged from the start and prevent them from dropping out or slowing down. The Theil-Sen technique allows for a robust line fitting as its estimation is obtained by calculating the median of the slopes of all possible pairs of datapoints. This non-parametric regression technique was opted for given the relatively small sample size and violated assumptions to perform ordinary least square regressions. Initial individual story appreciation and individual response accuracy at the start of the intervention were defined as a player’s mean story appreciation and mean question response accuracy of the first two game phases respectively (i.e., mean ratings of the first 12 stories and mean response accuracy for the first 24 questions). The third and last part of the result section tackles a possible growth in language comprehension. Using the ‘lmerTest’ package in R, we performed a multilevel linear growth model in the sample that finished at least 80% of the game (n = 74) with game phase as a within-subjects variable and baseline listening comprehension, morphological awareness, and receptive vocabulary as between-

6a-i) Online questionnaires: describe if they were validated for online use and apply CHERRIES items to describe how the questionnaires were designed/deployed

If outcomes were obtained through online questionnaires, describe if they were validated for online use and apply CHERRIES items to describe how the questionnaires were designed/deployed [9].

	1	2	3	4	5	
subitem not at all important	○	◉	○	○	○	essential

Selectie wissen

Does your paper address subitem 6a-i?

Copy and paste relevant sections from manuscript text

Jouw antwoord

6a-ii) Describe whether and how "use" (including intensity of use/dosage) was defined/measured/monitored

Describe whether and how "use" (including intensity of use/dosage) was defined/measured/monitored (logins, logfile analysis, etc.). Use/adoption metrics are important process outcomes that should be reported in any ehealth trial.

	1	2	3	4	5	
subitem not at all important	○	○	○	○	○	essential

Does your paper address subitem 6a-ii?

Copy and paste relevant sections from manuscript text

The use was defined and monitored. It was defined in the manuscript as follows: “After this baseline test-phase, the 91 at-risk children who were assigned to one of the three intervention groups received a tablet, its corresponding calibrated headphone, and a manual for parents with instructions to start the tablet intervention at home. With support of a reward calendar with stickers, they were instructed to combine story game sessions with 15-minute GG-FL/AC sessions for six days per week over a period of 12 weeks. This equaled a total of 72 gaming sessions, both for GG-FL/AC as well as for the story game.” Monitoring was done by checking the log-files, which is described in the manuscript as follows: “Useful player data of individual player accounts (e.g., game progress, question response accuracy, play dates and hours, and story rating information) were automatically logged on our university server and sent daily to the research group.”

6a-iii) Describe whether, how, and when qualitative feedback from participants was obtained

Describe whether, how, and when qualitative feedback from participants was obtained (e.g., through emails, feedback forms, interviews, focus groups).

	1	2	3	4	5	
subitem not at all important	○	○	○	◉	○	essential

Selectie wissen

Does your paper address subitem 6a-iii?

Copy and paste relevant sections from manuscript text

Qualitative feedback was obtained and described in the manuscript as follows: After the intervention, parents and children received a questionnaire assessing motivation, enjoyment, and engagement during the intervention period. After the intervention period, parents independently filled out a short questionnaire including three questions related to motivation, encouragement, and sustained attention during story gameplay. Accompanied by a member of the research group at school, intervention enjoyment was also measured in all children using two components of the Fun Toolkit survey instrument, which was developed to gather children's opinions on technology. On the one hand, children were asked to assign story game enjoyment on a five-point-Likert-scale-based Smiley-o-meter, in which the five scales were represented by smileys (ranging from a very unhappy smiley meaning 'I did not like the game at all' to a very happy smiley meaning 'I liked the game very much'). On the other hand, the participants were asked whether they were willing to redo the intervention (see Multimedia Appendix 3 for an overview of the five child and parental categorical questions and their response

6b) Any changes to trial outcomes after the trial commenced, with reasons

Does your paper address CONSORT subitem 6b? *

Copy and paste relevant sections from the manuscript (include quotes in quotation marks "like this" to indicate direct quotes from your manuscript), or elaborate on this item by providing additional information not in the ms, or briefly explain why the item is not applicable/relevant for your study

We did not make any changes to the trial outcomes after the trial commenced.

7a) How sample size was determined

NPT: When applicable, details of whether and how the clustering by care provides or centers was addressed

7a-i) Describe whether and how expected attrition was taken into account when calculating the sample size

Describe whether and how expected attrition was taken into account when calculating the sample size.

	1	2	3	4	5	
subitem not at all important	○	○	○	◉	○	essential

Selectie wissen

Does your paper address subitem 7a-i?

Copy and paste relevant sections from manuscript title (include quotes in quotation marks "like this" to indicate direct quotes from your manuscript), or elaborate on this item by providing additional information not in the ms, or briefly explain why the item is not applicable/relevant for your study

Attrition rate was not taken into account when calculating the samle size. Sample size was determined based on a power analysis to detect small or medium intervention effects (not described in the current manuscript). With 120 participants at cognitive risk for dyslexia (4 x approximately 30 – GGFL_NE, GGFL_EE, AC, and PC groups described in the manuscript), we have a power of 82% to detect small effect sizes (partial eta^2=0.02) in a repeated measures ANOVA with 3 measurements (pre-test, post-test and consolidation 1 year after the intervention), 4 groups and within-between interactions.

7b) When applicable, explanation of any interim analyses and stopping guidelines

Does your paper address CONSORT subitem 7b? *

Copy and paste relevant sections from the manuscript (include quotes in quotation marks "like this" to indicate direct quotes from your manuscript), or elaborate on this item by providing additional information not in the ms, or briefly explain why the item is not applicable/relevant for your study

We did not perform any interim analyses or we did not have stopping guidelines for the participants during the intervention period. We simply instructed parents to play according to the advised schedule as much as possible.

8a) Method used to generate the random allocation sequence

NPT: When applicable, how care providers were allocated to each trial group

Does your paper address CONSORT subitem 8a? *

Copy and paste relevant sections from the manuscript (include quotes in quotation marks "like this" to indicate direct quotes from your manuscript), or elaborate on this item by providing additional information not in the ms, or briefly explain why the item is not applicable/relevant for your study

Random allocation sequence of the three experimental groups (GGFL_EE, GGFL_NE, and AC) was done in R by using the Randomizr package:
[Coppock, A. (2019). randomizr: Easy-to-Use Tools for Common Forms of Random Assignment and Sampling. R package version 0.20.0. Available online at: https://cran.r-project.org/web/packages/randomizr/index.html (accessed August 23, 2021)]

8b) Type of randomisation; details of any restriction (such as blocking and block size)

Does your paper address CONSORT subitem 8b? *

Copy and paste relevant sections from the manuscript (include quotes in quotation marks "like this" to indicate direct quotes from your manuscript), or elaborate on this item by providing additional information not in the ms, or briefly explain why the item is not applicable/relevant for your study

The randomization procedure for the GGFL_EE, GGFL_NE and AC group was a block randomization procedure with stratified analysis. The main consideration for choosing a stratified analysis is that it adjusts for any bias that may result due to covariate imbalance, and ensures integrity of the study. In this case, the covariates included birth trimester, non-verbal IQ and educational environment. Birth trimester was characterized by three categories (1 = born in the first trimester of 2013, 2 = born in the second trimester of 2013, 3 = born in the third trimester of 2013). For the non-verbal IQ, we used standard scores. Educational environment was characterized by the classroom of the participant. Randomizing for these variables across the four different conditions was necessary to avoid confounding results. In the current study, given that we mainly wanted to investigate feasibility and enjoyment of the story game, all three randomized groups who played the story game were pooled together.

9) Mechanism used to implement the random allocation sequence (such as sequentially numbered containers), describing any steps taken to conceal the sequence until interventions were assigned

Does your paper address CONSORT subitem 9? *

Copy and paste relevant sections from the manuscript (include quotes in quotation marks "like this" to indicate direct quotes from your manuscript), or elaborate on this item by providing additional information not in the ms, or briefly explain why the item is not applicable/relevant for your study

Random allocation sequence of the three experimental groups (GGFL_EE, GGFL_NE, and AC) was done in R by using the Randomizr package in R. The randomization procedure for the GGFL_EE, GGFL_NE and AC group was a block randomization procedure with stratified analysis.

10) Who generated the random allocation sequence, who enrolled participants, and who assigned participants to interventions

Does your paper address CONSORT subitem 10? *

Copy and paste relevant sections from the manuscript (include quotes in quotation marks "like this" to indicate direct quotes from your manuscript), or elaborate on this item by providing additional information not in the ms, or briefly explain why the item is not applicable/relevant for your study

The randomization code in R was written and ran by a member of the research group.

11a) If done, who was blinded after assignment to interventions (for example, participants, care providers, those assessing outcomes) and how

NPT: Whether or not administering co-interventions were blinded to group assignment

11a-i) Specify who was blinded, and who wasn't

Specify who was blinded, and who wasn't. Usually, in web-based trials it is not possible to blind the participants [1, 3] (this should be clearly acknowledged), but it may be possible to blind outcome assessors, those doing data analysis or those administering co-interventions (if any).

	1	2	3	4	5	
subitem not at all important	○	○	○	●	○	essential

Selectie wissen

Does your paper address subitem 11a-i? *

Copy and paste relevant sections from the manuscript (include quotes in quotation marks "like this" to indicate direct quotes from your manuscript), or elaborate on this item by providing additional information not in the ms, or briefly explain why the item is not applicable/relevant for your study

Members of the research group were not blinded and knew which participant was allocated to which group. However, several job students and master thesis students assisted in the data collection and they never received information about group allocation of the participants. Parents of the participants were informed whether their child was at cognitive risk for dyslexia or not at the start of the intervention study. However, the at-risk participants who were assigned to either the GGFL_EE, GGFL_NE or AC group did not know which group their child belonged to until the end of the first consolidation data collection point (approximately one year after the digital intervention). Parents of at-risk participants in the passive control group (not included in the data analysis of the current study) were ofcourse aware that their child belonged to this group, since their child received no type of intervention.

11a-ii) Discuss e.g., whether participants knew which intervention was the “intervention of interest” and which one was the “comparator”

Informed consent procedures (4a-ii) can create biases and certain expectations - discuss e.g., whether participants knew which intervention was the “intervention of interest” and which one was the “comparator”.

	1	2	3	4	5	
subitem not at all important	○	○	○	●	○	essential

Selectie wissen

Does your paper address subitem 11a-ii?

Copy and paste relevant sections from the manuscript (include quotes in quotation marks "like this" to indicate direct quotes from your manuscript), or elaborate on this item by providing additional information not in the ms, or briefly explain why the item is not applicable/relevant for your study

Two versions of the story game existed: one with and one without envelope enhancement processing. The original goal of the story game was to induce a boosting effect on reading and phonology on top of GG-FL (not discussed in the current study). Envelope enhancement results in an enhancement of the amplitude rise times of the speech envelope, leaving the rest of the speech signal unchanged. Recent evidence suggests that participants cannot hear the difference between enhanced and non-enhanced sentences. Thus participants were unaware whether they listened to enhanced (intervention of interest) versus not-enhanced stories (the comparator, but not intended as a comparator in the current study). Parents also just received information about the possibility that the stories could be signal processed, but did not know whether this was the case or not.

11b) If relevant, description of the similarity of interventions

(this item is usually not relevant for ehealth trials as it refers to similarity of a placebo or sham intervention to a active medication/intervention)

Does your paper address CONSORT subitem 11b? *

Copy and paste relevant sections from the manuscript (include quotes in quotation marks "like this" to indicate direct quotes from your manuscript), or elaborate on this item by providing additional information not in the ms, or briefly explain why the item is not applicable/relevant for your study

As discussed in item 11a-ii, the story game, which was played by the GGFL_EE, GGFL_NE and the AC group, was exactly the same with the only difference that in the GG_EE group, the recorded speech signal of the stories contained envelope enhancement. In the remaining two groups, no signal processing was applied to the speech signal. In the current study, these so-called NE or EE story game interventions were not compared against each other, since this was not the purpose of the current study.

12a) Statistical methods used to compare groups for primary and secondary outcomes

NPT: When applicable, details of whether and how the clustering by care providers or centers was addressed

Does your paper address CONSORT subitem 12a? *

Copy and paste relevant sections from the manuscript (include quotes in quotation marks "like this" to indicate direct quotes from your manuscript), or elaborate on this item by providing additional information not in the ms, or briefly explain why the item is not applicable/relevant for your study

The current study was in fact not a randomized controlled trial and did therefore not compare intervention and control groups. For the purposes of the current study, all children who played the story game were pooled together. However, in a future study, in which the effect of envelope enhancement will be investigated, linear mixed models will be used in order to correctly investigate the boosting effect.

12a-i) Imputation techniques to deal with attrition / missing values

Imputation techniques to deal with attrition / missing values: Not all participants will use the intervention/comparator as intended and attrition is typically high in ehealth trials. Specify how participants who did not use the application or dropped out from the trial were treated in the statistical analysis (a complete case analysis is strongly discouraged, and simple imputation techniques such as LOCF may also be problematic [4]).

	1	2	3	4	5	
subitem not at all important	○	○	◉	○	○	essential

Selectie wissen

Does your paper address subitem 12a-i? *

Copy and paste relevant sections from the manuscript (include quotes in quotation marks "like this" to indicate direct quotes from your manuscript), or elaborate on this item by providing additional information not in the ms, or briefly explain why the item is not applicable/relevant for your study

Dealing with attrition rate and unvalid data was described in the manuscript as follows: “Of the 91 children that were asked to play the story game, seven children were excluded from the dataset because of technical game problems during the intervention period (bugs, explained in Multimedia Appendix 1). Two additional children were excluded because they never started the general digital gaming intervention properly (i.e., they played less than 10% of GG-FL/AC and the story game). Hence, the final number of children for whom useful story game data were available was 82. As for predicting game exposure based on initial story appreciation or initial individual question response accuracy, the analyses were restricted to the sample of players that did not completely finish the story game (n = 32) as including participants who completed the full intervention would render a ceiling effect in the results. As for predicting training duration based on initial story appreciation or individual response accuracy, we excluded eight participants who played less than 80% of the total game content, since their training duration in days did not represent a reliable intervention trajectory. As such, training duration was predicted based on data of 74 participants.”

12b) Methods for additional analyses, such as subgroup analyses and adjusted analyses

Does your paper address CONSORT subitem 12b? *

Copy and paste relevant sections from the manuscript (include quotes in quotation marks "like this" to indicate direct quotes from your manuscript), or elaborate on this item by providing additional information not in the ms, or briefly explain why the item is not applicable/relevant for your study

Subgroup analyses were discussed in the paper as follows: As for predicting game exposure based on initial story appreciation or initial individual question response accuracy, the analyses were restricted to the sample of players that did not completely finish the story game (n = 32) as including participants who completed the full intervention would render a ceiling effect in the results. As for predicting training duration based on initial story appreciation or individual response accuracy, we excluded eight participants who played less than 80% of the total game content, since their training duration in days did not represent a reliable intervention trajectory. As such, training duration was predicted based on data of 74 participants.” We want to emphasize that these analyses were not considered as additional analyses, but they answered the main research questions stated in the manuscript.

X26) REB/IRB Approval and Ethical Considerations [recommended as subheading under "Methods"] (not a CONSORT item)

X26-i) Comment on ethics committee approval

	1	2	3	4	5	
subitem not at all important	○	○	○	○	◉	essential

Selectie wissen

Does your paper address subitem X26-i?

Copy and paste relevant sections from the manuscript (include quotes in quotation marks "like this" to indicate direct quotes from your manuscript), or elaborate on this item by providing additional information not in the ms, or briefly explain why the item is not applicable/relevant for your study

Ethics committee approval was stated in the manuscript as follows: “Signed informed consents were obtained for all the participants and the study was approved by the Medical Ethical Committee of UZ Leuven, KU Leuven.”

x26-ii) Outline informed consent procedures

Outline informed consent procedures e.g., if consent was obtained offline or online (how? Checkbox, etc.?), and what information was provided (see 4a-ii). See [6] for some items to be included in informed consent documents.

	1	2	3	4	5	
subitem not at all important	○	○	○	◉	○	essential

Selectie wissen

Does your paper address subitem X26-ii?

Copy and paste relevant sections from the manuscript (include quotes in quotation marks "like this" to indicate direct quotes from your manuscript), or elaborate on this item by providing additional information not in the ms, or briefly explain why the item is not applicable/relevant for your study

Informed consent letters were provided by e-mail to the parents of the participants after they were contacted by phone in order to inform them about the potential cognitive risk for dyslexia of their child. After they approved on the phone to participate in the intervention study, an informed consent letter was provided to them when they came to the lab with their child for a first MRI session (the intervention study included MRI, EEG, and behavioral data collection points at school). By signing the informed consent, parents approved to participate in the MRI, EEG, and school data collection points before, immediately after and one year after the intervention. The current study only included in-game intervention data, baseline language skills (measured during the pre-test session at school) and post-intervention questionnaire data.

X26-iii) Safety and security procedures

Safety and security procedures, incl. privacy considerations, and any steps taken to reduce the likelihood or detection of harm (e.g., education and training, availability of a hotline)

	1	2	3	4	5	
subitem not at all important	○	○	○	◉	○	essential

Selectie wissen

Does your paper address subitem X26-iii?

Copy and paste relevant sections from the manuscript (include quotes in quotation marks "like this" to indicate direct quotes from your manuscript), or elaborate on this item by providing additional information not in the ms, or briefly explain why the item is not applicable/relevant for your study

Safety and security procedures related to the intervention and behavioal measurements were discussed in the informed consent letter to the parents as follows: The behavioral data collection points and the intervention do not include any risks. Data will be coded. There continues to be a link between the data and the individual who provided it. The research team will protect the data from disclosure outside the research according to the terms of the research protocol and the informed consent document. The subject’s name or other identifiers will be stored separately (site file) from their research data and replaced with a unique code to create a new identity for the subject.

RESULTS

13a) For each group, the numbers of participants who were randomly assigned, received intended treatment, and were analysed for the primary outcome

NPT: The number of care providers or centers performing the intervention in each group and the number of patients treated by each care provider in each center

Does your paper address CONSORT subitem 13a? *

Copy and paste relevant sections from the manuscript (include quotes in quotation marks "like this" to indicate direct quotes from your manuscript), or elaborate on this item by providing additional information not in the ms, or briefly explain why the item is not applicable/relevant for your study

The number of participants in each group were described in the manuscript as follows: "Of the entire intervention study sample (n=149), 91 at-risk children were asked to play the story game. Within the framework of the reading intervention study, these 91 children were randomly assigned to one of three experimental groups. The first group (GGFL_EE group, n=31) played the story game with envelope enhanced stories and combined it with the phonics-based GG-FL intervention [54]. For technical details of the EE algorithm, consider the study of Van Herck and colleagues [49]. The second group received the same intervention as the first with the only difference that there was no envelope enhancement applied to the stories in the story game (GGFL_NE group, n=31). The third group also played the non-enhanced story game and combined it with tablet-based commercial Lego- and Duplo-applications (henceforth active control (AC) games), which did not train any reading-related skills (AC_NE group, n=29). The remaining 28 at-risk and 30 non-at-risk children of the total sample served as the at-risk passive control and typically developing control group of the reading intervention study respectively and did not receive any type of digital gaming intervention. Since the current study mainly focuses on the story game feasibility and enjoyment, these last two control groups, who did not play the story game, will not be discussed any further in the current research article."

The number of participants that were included in the dataset for the purpose of the current study was described in the manuscript as follows: "Of the 91 children that were asked to play the story game, seven children were excluded from the dataset because of technical game problems during the intervention period (bugs, explained in Multimedia Appendix 1). Two additional children were excluded because they never started the general digital gaming intervention properly (i.e., they played less than 10% of GG-FL/AC and the story game). Hence, the final number of children for whom useful story game data were available was 82."

13b) For each group, losses and exclusions after randomisation, together with reasons

Does your paper address CONSORT subitem 13b? (NOTE: Preferably, this is shown in a CONSORT flow diagram) *

Copy and paste relevant sections from the manuscript (include quotes in quotation marks "like this" to indicate direct quotes from your manuscript), or elaborate on this item by providing additional information not in the ms, or briefly explain why the item is not applicable/relevant for your study

Losses and exclusions after randomization were described in the manuscript as follows: “Of the 91 children that were asked to play the story game, seven children were excluded from the dataset because of technical game problems during the intervention period (bugs, explained in Multimedia Appendix 1). Two additional children were excluded because they never started the general digital gaming intervention properly (i.e., they played less than 10% of GG-FL/AC and the story game). Hence, the final number of children for whom useful story game data were available was 82.”

Given that for the purpose of the current study, all groups who played the story game were pooled together, we did not discuss losses and exclusions for each intervention group separately.

13b-i) Attrition diagram

Strongly recommended: An attrition diagram (e.g., proportion of participants still logging in or using the intervention/comparator in each group plotted over time, similar to a survival curve) or other figures or tables demonstrating usage/dose/engagement.

	1	2	3	4	5	
subitem not at all important	○	○	○	●	○	essential

Selectie wissen

Does your paper address subitem 13b-i?

Copy and paste relevant sections from the manuscript or cite the figure number if applicable (include quotes in quotation marks "like this" to indicate direct quotes from your manuscript), or elaborate on this item by providing additional information not in the ms, or briefly explain why the item is not applicable/relevant for your study

We clearly address subitem 13b-i by providing a figure which represents the attrition rate by showing the proportion of active participants and drop-outs throughout the entire intervention progress (Figure 8 in the manuscript).

14a) Dates defining the periods of recruitment and follow-up

Does your paper address CONSORT subitem 14a? *

Copy and paste relevant sections from the manuscript (include quotes in quotation marks "like this" to indicate direct quotes from your manuscript), or elaborate on this item by providing additional information not in the ms, or briefly explain why the item is not applicable/relevant for your study

We did not provide the exact dates when the recruitment and intervention phase took place. However, we state in our manuscript that the screening and the intervention took place when children were in the second semester of the last and third year of kindergarten. This was in the period between January and June 2019, depending on when a participant started.

14a-i) Indicate if critical “secular events” fell into the study period

Indicate if critical “secular events” fell into the study period, e.g., significant changes in Internet resources available or “changes in computer hardware or Internet delivery resources”

	1	2	3	4	5	
subitem not at all important	○	○	◉	○	○	essential

Selectie wissen

Does your paper address subitem 14a-i?

Copy and paste relevant sections from the manuscript (include quotes in quotation marks "like this" to indicate direct quotes from your manuscript), or elaborate on this item by providing additional information not in the ms, or briefly explain why the item is not applicable/relevant for your study

No secular events fell into the study period.

14b) Why the trial ended or was stopped (early)

Does your paper address CONSORT subitem 14b? *

Copy and paste relevant sections from the manuscript (include quotes in quotation marks "like this" to indicate direct quotes from your manuscript), or elaborate on this item by providing additional information not in the ms, or briefly explain why the item is not applicable/relevant for your study

The trial did not stop earlier than intended. Every particpant was instructed to play the games (story game combined with either GGFL or AC) according to the advised schedule.

15) A table showing baseline demographic and clinical characteristics for each group

NPT: When applicable, a description of care providers (case volume, qualification, expertise, etc.) and centers (volume) in each group

Does your paper address CONSORT subitem 15? *

Copy and paste relevant sections from the manuscript (include quotes in quotation marks "like this" to indicate direct quotes from your manuscript), or elaborate on this item by providing additional information not in the ms, or briefly explain why the item is not applicable/relevant for your study

Given that all three groups who played the story game were pooled together for the purpose of the current study, we did not provide baseline demographic and clinical characteristics per group.

15-i) Report demographics associated with digital divide issues

In ehealth trials it is particularly important to report demographics associated with digital divide issues, such as age, education, gender, social-economic status, computer/Internet/ehealth literacy of the participants, if known.

	1	2	3	4	5	
subitem not at all important	○	○	◉	○	○	essential

Selectie wissen

Does your paper address subitem 15-i? *

Copy and paste relevant sections from the manuscript (include quotes in quotation marks "like this" to indicate direct quotes from your manuscript), or elaborate on this item by providing additional information not in the ms, or briefly explain why the item is not applicable/relevant for your study

We did not report demographics associated with digital divide issues, because we believed that nowadays, 5-year old children in Flanders have enough experience with tablets. Moreover, the story game was developed in such a way that game mechanics were self-explanatory and easy to use. This was also discussed in the GameFlow-based evaluation (Table 3 in the manuscript) as follows: “All actions required the use of touch screen, since the game was tablet-based. Nowadays, in Western society, the majority of five-year olds are familiar with these devices. Furthermore, players only had a limited set of actions, as advanced game options were locked with a password. These limited actions (e.g., stopping, starting, or continuing the story game, selecting a response for the questions, buying accessories in the avatar customization system) were all self-explanatory (e.g., selecting the correct response or a desired accessory by touch screen) or assigned with clear symbols on the screen (red arrow to stop the game, large green play-symbol to start or continue the story recording).”

16) For each group, number of participants (denominator) included in each analysis and whether the analysis was by original assigned groups

16-i) Report multiple “denominators” and provide definitions

Report multiple “denominators” and provide definitions: Report N’s (and effect sizes) “across a range of study participation [and use] thresholds” [1], e.g., N exposed, N consented, N used more than x times, N used more than y weeks, N participants “used” the intervention/comparator at specific pre-defined time points of interest (in absolute and relative numbers per group). Always clearly define “use” of the intervention.

	1	2	3	4	5	
subitem not at all important	○	○	○	○	○	essential

Does your paper address subitem 16-i? *

Copy and paste relevant sections from the manuscript (include quotes in quotation marks "like this" to indicate direct quotes from your manuscript), or elaborate on this item by providing additional information not in the ms, or briefly explain why the item is not applicable/relevant for your study

For each analysis, denominators were provided:

Enjoyment

Post-intervention questionnaires:
“54 participants (66%) confirmed that they would be willing to redo the training (see right panel of Figure 4). Post-intervention parental questionnaires revealed (1) that the majority of parents observed a high (n = 39, 47%) or relatively high play motivation (n = 32, 39%) in their child (see left panel of Figure 5), (2) that most of the players never (n = 43, 52%) or only sometimes (n = 25, 30%) needed encouragement to play (see middle panel of Figure 5), and (3) that more than three quarters of the parents (n = 65, 79%) observed a state of sustained attention during gameplay (see right panel of Figure 5).”

In-game data related to fame enjoyment:
“Each story was liked by more than 75% of the listeners for whom data were available (i.e., more than 62 of the 82 listeners), indicating that the stories were enjoyable.”

Feasibility

General question response accuracy:
“All but 11 questions were answered correctly by 75% of the listeners for whom data were available (e.g., 62 of the 82 listeners), suggesting a rather stable difficulty level of the questions.”

Attrition rate:
“Of the 82 players, 90% (n = 74) listened to 80% of the stories (~ 57th session of the total 72 story sessions) and 61% (n = 50) managed to finish the game completely. Hence, 39% of the players (n = 32) dropped out at some point during the 12-week intervention.”

Final game exposure, training duration and gaming profiles:
“Based on the categorization criteria (complete/incomplete and compliant/non-compliant), we established three different gaming profiles: (1) complete and compliant players (n = 18) (upper panel of Figure 10), (2) complete, but non-compliant players (n = 32) (middle panel of Figure 10), and (3) incomplete and non-compliant players (n = 32) (lower panel of Figure 10). The Theil-Sen regression analysis revealed that the mean story appreciation of the first two game phases was not significantly predictive of final game exposure among the 32 children who dropped out at some point (β = 0.15, P = .08). In contrast, the Theil-Sen regression analysis revealed that the mean question response accuracy of the first two game phases significantly predicted final game exposure, such that a lower accuracy at the start accelerated the drop-out point (β = 0.35, P < .001) (see right panel of Figure 11). Story appreciation of the first two game phases significantly predicted final training duration in a negative way among those 74 children who finished 80% of the game (β = -0.16, P = .003) (left panel of Figure 12). Mean question response accuracy at the start did not significantly predict final training duration (β = 0.00, P = .68) among the children who finished or almost completely

finished the story game (right panel of Figure 12).”

Growth in language comprehension

"Using the ‘lmerTest’ package in R, we performed a multilevel linear growth model in the sample that finished at least 80% of the game (n = 74) with game phase as a within-subjects variable and baseline listening comprehension. Model outcomes for the linear mixed effect model are presented in Table 9.”

16-ii) Primary analysis should be intent-to-treat

Primary analysis should be intent-to-treat, secondary analyses could include comparing only “users”, with the appropriate caveats that this is no longer a randomized sample (see 18-i).

	1	2	3	4	5	
subitem not at all important	○	○	○	◉	○	essential

Selectie wissen

Does your paper address subitem 16-ii?

Copy and paste relevant sections from the manuscript (include quotes in quotation marks "like this" to indicate direct quotes from your manuscript), or elaborate on this item by providing additional information not in the ms, or briefly explain why the item is not applicable/relevant for your study

The current study did not investigate the educational effects of the intervention based on a randomized controlled trial design. Therefore, subitem 16-ii is not applicable for our study.

17a) For each primary and secondary outcome, results for each group, and the estimated effect size and its precision (such as 95% confidence interval)

Does your paper address CONSORT subitem 17a? *

Copy and paste relevant sections from the manuscript (include quotes in quotation marks "like this" to indicate direct quotes from your manuscript), or elaborate on this item by providing additional information not in the ms, or briefly explain why the item is not applicable/relevant for your study

The current study did not investigate the educational effects of the intervention based on a randomized controlled trial design. Therefore, subitem 17a is not applicable for our study.

17a-i) Presentation of process outcomes such as metrics of use and intensity of use

In addition to primary/secondary (clinical) outcomes, the presentation of process outcomes such as metrics of use and intensity of use (dose, exposure) and their operational definitions is critical. This does not only refer to metrics of attrition (13-b) (often a binary variable), but also to more continuous exposure metrics such as “average session length”. These must be accompanied by a technical description how a metric like a “session” is defined (e.g., timeout after idle time) [1] (report under item 6a).

	1	2	3	4	5	
subitem not at all important	○	○	○	●	○	essential

Selectie wissen

Does your paper address subitem 17a-i?

Copy and paste relevant sections from the manuscript (include quotes in quotation marks "like this" to indicate direct quotes from your manuscript), or elaborate on this item by providing additional information not in the ms, or briefly explain why the item is not applicable/relevant for your study

Figure 9 in the result section provides an overview of the individuals’ game exposure and training duration and their overrall distributions. This plot clearly shows the metrics and intensity of use for each participant. Moreover, participants were categorized based on completeness and compliantness in three groups (complete and compliant, incomplete and incompliant, complete and incompliant). This categorization also gives information about the metrics and intensity of use.

17b) For binary outcomes, presentation of both absolute and relative effect sizes is recommended

Does your paper address CONSORT subitem 17b? *

Copy and paste relevant sections from the manuscript (include quotes in quotation marks "like this" to indicate direct quotes from your manuscript), or elaborate on this item by providing additional information not in the ms, or briefly explain why the item is not applicable/relevant for your study

We do not include binary variables in the current study.

18) Results of any other analyses performed, including subgroup analyses and adjusted analyses, distinguishing pre-specified from exploratory

Does your paper address CONSORT subitem 18? *

Copy and paste relevant sections from the manuscript (include quotes in quotation marks "like this" to indicate direct quotes from your manuscript), or elaborate on this item by providing additional information not in the ms, or briefly explain why the item is not applicable/relevant for your study

We did not preregister our research questions and analyses, but we analyzed all data within the framework of our presupposed research questions, without performing additional analyses afterwards.

18-i) Subgroup analysis of comparing only users

A subgroup analysis of comparing only users is not uncommon in ehealth trials, but if done, it must be stressed that this is a self-selected sample and no longer an unbiased sample from a randomized trial (see 16-iii).

	1	2	3	4	5	
subitem not at all important	○	○	○	●	○	essential

Selectie wissen

Does your paper address subitem 18-i?

Copy and paste relevant sections from the manuscript (include quotes in quotation marks "like this" to indicate direct quotes from your manuscript), or elaborate on this item by providing additional information not in the ms, or briefly explain why the item is not applicable/relevant for your study

Given that all groups who were playing the game were pooled together as one group, we did not compare different intervention groups in the current study. Sub-item 18-i is thus not applicable for the current study.

19) All important harms or unintended effects in each group

(for specific guidance see CONSORT for harms)

Does your paper address CONSORT subitem 19? *

Copy and paste relevant sections from the manuscript (include quotes in quotation marks "like this" to indicate direct quotes from your manuscript), or elaborate on this item by providing additional information not in the ms, or briefly explain why the item is not applicable/relevant for your study

Given that all groups who were playing the game were pooled together as one group, we did not compare different intervention groups and as a result, potential harms, in the current study. Item 19 is thus not applicable for the current study. However, as we stated in the informed consent letters to the parents, we did not expect harmful effects of the intervention (since it was simply playing child-friendly tablet games, for approximately 25 minutes per day in total, over a period of 12 weeks).

19-i) Include privacy breaches, technical problems

Include privacy breaches, technical problems. This does not only include physical “harm” to participants, but also incidents such as perceived or real privacy breaches [1], technical problems, and other unexpected/unintended incidents. “Unintended effects” also includes unintended positive effects [2].

	1	2	3	4	5	
subitem not at all important	○	○	○	◉	○	essential

Selectie wissen

Does your paper address subitem 19-i?

Copy and paste relevant sections from the manuscript (include quotes in quotation marks "like this" to indicate direct quotes from your manuscript), or elaborate on this item by providing additional information not in the ms, or briefly explain why the item is not applicable/relevant for your study

Technical problems occurred in a subsample of the children, playing the story game: “The game was piloted many times on members of the research group and on children with a similar age as the target group. Although the game appeared bug-free at the end of the pilot studies, some players in the actual intervention study (not included in the final data analysis) experienced bugs causing a total crash of the game and loss of in-game data. In those cases, a member of the research group provided support as soon as possible by reinstalling the game at the homes of the players.
The aforementioned bugs were fully described in detail in Multimedia Appendix 1 as follows: “When a player exited the game via the intended red arrow in the main menu, the game saved the currently active profile one last time. Yet, if that active profile had been previously deleted for some reason in the main configuration system, it would just be rewritten back, making it seemingly impossible to delete that profile. This bug could be circumvented by closing the game externally, for example via the Android task manager, yet it should be fixed in future versions of the story game. Account-related .sav files usually contained a name structure, such as “Name-ID.sav”. However, they were sometimes inexplicably saved without any name or ID specification, e.g., “.sav” (i.e., blank name saving). This bug messed with any code trying to save, load or delete the existing account-related Name-ID.sav files and sometimes even replaced the existing Name-ID.sav file on the tablet, causing loss of data. Moreover, this blank name saving bug sometimes resulted in the occurrence of another bug, i.e., the so-called “Bobby bug”. More specifically, when the code failed to load an account-related profile, the name “Bobby” appeared on the main menu screen, instead of the name of the participant’s account, as it was used as a place holder name to test the user interface of the game in the developmental phase. Moreover, in this case, the story game only played the first sub-phase of the first game phase over and over again. Occurrence of these bugs in the current intervention study resulted in a number of home visits where members of the research group fully reset the entire game and existing profiles. Yet, thanks to the existence of the AutoSyncer application, this bug only caused a limited amount of data loss (i.e., in seven participants). However, when applying the game in future studies, fixing these bugs must take the highest priority.”

19-ii) Include qualitative feedback from participants or observations from staff/researchers

Include qualitative feedback from participants or observations from staff/researchers, if available, on strengths and shortcomings of the application, especially if they point to unintended/unexpected effects or uses. This includes (if available) reasons for why people did or did not use the application as intended by the developers.

	1	2	3	4	5	
subitem not at all important	○	○	○	◉	○	essential

Selectie wissen

Does your paper address subitem 19-ii?

Copy and paste relevant sections from the manuscript (include quotes in quotation marks "like this" to indicate direct quotes from your manuscript), or elaborate on this item by providing additional information not in the ms, or briefly explain why the item is not applicable/relevant for your study

Qualitative feedback related to enjoyment of the game was given by the participants and their parents based on post-intervention questionnaires. Results of these categorical questionnaire outcomes were clearly visualized in Figure 4 and 5 in the manuscript.

DISCUSSION

22) Interpretation consistent with results, balancing benefits and harms, and considering other relevant evidence

NPT: In addition, take into account the choice of the comparator, lack of or partial blinding, and unequal expertise of care providers or centers in each group

22-i) Restate study questions and summarize the answers suggested by the data, starting with primary outcomes and process outcomes (use)

Restate study questions and summarize the answers suggested by the data, starting with primary outcomes and process outcomes (use).

	1	2	3	4	5	
subitem not at all important	○	○	○	○	◉	essential

Selectie wissen

Does your paper address subitem 22-i? *

Copy and paste relevant sections from the manuscript (include quotes in quotation marks "like this" to indicate direct quotes from your manuscript), or elaborate on this item by providing additional information not in the ms, or briefly explain why the item is not applicable/relevant for your study

Study questions and primary outcomes are restated in the discussion (principal outcomes section) as follows:
“The current study addressed enjoyment and feasibility of a story listening game and preliminarily assessed a possible growth in language comprehension. Principal findings on the game enjoyment, feasibility, and the impact of the game on language comprehension will be disclosed below.
As for the enjoyment evaluation, child and parental questionnaires and in-game enjoyment-related data (e.g., story appreciation) point to a highly positive game experience. Nevertheless, although a considerable amount of GameFlow criteria were fully and partly implemented in the story game, probably benefitting game enjoyment experience to a large extent, the GameFlow-model based evaluation also implied room for optimization of the game design. Concerning the feasibility of the intervention in terms of difficulty, results suggest that the game was adjusted to the cognitive capacities of five year old children with an elevated risk for dyslexia, since almost all questions were answered correctly by minimally three quarters of the listeners. As for predicting individuals’ final training duration and game exposure, we established a significantly negative relationship between initial mean story appreciation and final training duration and a positive relationship between initial mean question response accuracy and final game exposure. As for the growth in language comprehension, we established an increase in mean question response accuracy along with game phase, baseline vocabulary, and baseline listening comprehension. Yet, most strikingly, children with lower listening ability scores at the start of the intervention period made significantly more progress in terms of question response accuracy than children with higher pre-intervention listening ability scores.”

22-ii) Highlight unanswered new questions, suggest future research

Highlight unanswered new questions, suggest future research.

	1	2	3	4	5	
subitem not at all important	○	○	○	○	◉	essential

Selectie wissen

Does your paper address subitem 22-ii?

Copy and paste relevant sections from the manuscript (include quotes in quotation marks "like this" to indicate direct quotes from your manuscript), or elaborate on this item by providing additional information not in the ms, or briefly explain why the item is not applicable/relevant for your study

Unanswered questions and suggestions for future research related to game enjoyment are specified in the manuscript as follows: “Optimizing the partly or non-fulfilled criteria (e.g., fixing the remaining bugs, clarifying the end goals) could increase the level of enjoyment even more. To deal with the subjectivity, a future study could let adults who were not involved in the design process (e.g., parents of playing children) rate the game based on the 0-5 rating procedure.”

Unanswered questions and suggestions for future research related to feasibility are specified in the manuscript as follows: “Distinguishing completers from non-completers based on intrinsic and extrinsic features did not belong to the scope of the current study, albeit the research methods applied in the study of Justice and colleagues [80] offer possibilities for future research which could be of added value to optimize feasibility of the current intervention program.
Hence, albeit the need for statistical confirmation in a future study, spacing out the story game intervention over time in the current study might have even benefitted the expected educational learning outcomes. The outcomes of these predictive analyses point to limitations in the current game design, but give rise to suggestions for optimizing intervention feasibility in future studies. For example, given the importance of initial story appreciation to stick at the advised training intensity, a possible suggestion involves offering the story book series in the preferred order of the participant. More specifically, providing a main catalog menu in which players could choose which story series occur first might increase the story appreciation at the beginning and as a result, the engagement to follow the advised schedule. Moreover, given the role of initial question response accuracy in drop-out occurrence, offering player-adapted questions based on an individual’s language knowledge and cognitive capacities, which then increase in difficulty, instead of fixed predefined questions for all participants, might prevent participants to withdraw from the study. A more interactive approach in which players could request and receive explanations of possible difficult words might also increase the chance to already successfully respond to the questions from the start, lowering the chances of early drop-out.”

Unanswered questions and suggestions for future research related to language comprehension growth are specified in the manuscript as follows:
“The actual impact of the game on language comprehension can only be solidly established by conducting a randomized controlled trial (RCT), which was not performed in the current study. Hence, although we found a larger growth in mean question response accuracy in children with lower listening comprehension, the current research design does actually not allow us to draw univocal conclusions on the potential of the game to train language comprehension. A future RCT study, preferably including (1) a group that is purely playing the story game without combining it with other interventions such as GG-FL or active control games, (2) a no-intervention control group, and (3) a control group receiving an alternative placebo treatment which does not specifically train language skills, is thus of crucial relevance to further disentangle the gaming effects on language comprehension. We are unsure whether all questions and stories implemented in the game were of equal difficulty, although we found relatively stable response distributions in the majority of the questions. Hence, changes in performance might not be due to the intervention effects, but simply to the varying difficulty of the questions and stories. Conducting pre- and post-intervention

[illegible] measurements of independent language comprehension tests within an RCT design with appropriate control groups (as aforementioned), would eliminate this concern. Based on these aforementioned limitations, the preliminary and cautiously interpreted results concerning a possible growth in language comprehension give rise to three important ideas for story game improvement if it would actually be used to train language comprehension at some point in populations with specific problems in this domain (e.g., children with low SES, bilingual children, children with DLD, with a hearing impairment, or dyslexia). First, there is a clear need to also include inferential questions, since Tarvainen and colleagues emphasized the importance of inferential language training to foster language comprehension in preschoolers. Second, apart from optimizing the research design by conducting RCTs with adequate intervention and control groups, it is also of crucial relevance to include test batteries, which assess comprehension both at the inferential and literal level. Last, researchers must take into account and tackle the possibility that children with lower language levels might show less motivation in storybook interventions than their typically developing peers."

20) Trial limitations, addressing sources of potential bias, imprecision, and, if relevant, multiplicity of analyses

20-i) Typical limitations in ehealth trials

Typical limitations in ehealth trials: Participants in ehealth trials are rarely blinded. Ehealth trials often look at a multiplicity of outcomes, increasing risk for a Type I error. Discuss biases due to non-use of the intervention/usability issues, biases through informed consent procedures, unexpected events.

	1	2	3	4	5	
subitem not at all important	○	○	○	○	◉	essential

Selectie wissen

Does your paper address subitem 20-i? *

Copy and paste relevant sections from the manuscript (include quotes in quotation marks "like this" to indicate direct quotes from your manuscript), or elaborate on this item by providing additional information not in the ms, or briefly explain why the item is not applicable/relevant for your study

Given that the study described in the manuscript was part of a large-scale intervention study but not a randomized controlled trial, we were unable to describe multiplicity of outcomes or limitations related to blindedness. However, several other limitations related to our findings are described in the manuscript.

Limitations related to the enjoyment-related outcomes are described as follows:
"We acknowledge that the agreement of the developed game with the GameFlow model contains a form of subjectivity, as the evaluation was performed by two members of the research group who were involved in the design process itself."

Limitations related to the feasbility-related outcomes are described as follows:
"An important sidenote relates to the fact that participants in the current study combined the intervention with another tablet game (GG-FL or AC games). This could also have impacted general motivation and perseverance.
The last feasibility-related discussion point is linked to the prediction of final game exposure and training duration based on story appreciation and question response accuracy at the start of the intervention. The outcomes of these predictive analyses point to limitations in the current game design, but give rise to suggestions for optimizing intervention feasibility in future studies."

Limitations related to the investigation of language comprehension growth are described as follows:
"The first and most important limitation relates to the research design.
A second important limitation relates to the concept of content validity. We considered the in-game questions as a measure of language comprehension. However, albeit the selection of age-appropriate stories based on library visits and the construction of the questions based on the vocabulary of the story content, the stories and questions did not belong to a validated comprehension test instrument, casting doubts on the certainty that we truly measured language comprehension in our participants.
The third limitation discusses the distribution of the mean question response accuracy in the first game phase, which already showed a tendency towards ceiling. Hence, children with high accuracy scores from the start, related to a higher baseline vocabulary and listening comprehension, did not have the potential to increase any further during the intervention, influencing the interpretation of the game phase-listening comprehension interaction."

21) Generalisability (external validity, applicability) of the trial findings

NPT: External validity of the trial findings according to the intervention, comparators, patients, and care providers or centers involved in the trial

21-i) Generalizability to other populations

Generalizability to other populations: In particular, discuss generalizability to a general Internet population, outside of a RCT setting, and general patient population, including applicability of the study results for other organizations

	1	2	3	4	5	
subitem not at all important	○	○	○	○	◉	essential

Selectie wissen

Does your paper address subitem 21-i?

Copy and paste relevant sections from the manuscript (include quotes in quotation marks "like this" to indicate direct quotes from your manuscript), or elaborate on this item by providing additional information not in the ms, or briefly explain why the item is not applicable/relevant for your study

The generalizability (and its challenges) to other populations is briefly discussed in the manuscript as follows: “Based on these aforementioned limitations, the preliminary and cautiously interpreted results concerning a possible growth in language comprehension give rise to three important ideas for story game improvement if it would actually be used to train language comprehension at some point in populations with specific problems in this domain (e.g., children with low SES, bilingual children, children with DLD, with a hearing impairment, or dyslexia). First, there is a clear need to also include inferential questions, since Tarvainen and colleagues emphasized the importance of inferential language training to foster language comprehension in preschoolers. Second, apart from optimizing the research design by conducting RCTs with adequate intervention and control groups, it is also of crucial relevance to include test batteries, which assess comprehension both at the inferential and literal level. The currently used CELF-4-NL and the Peabody Picture Vocabulary-III-NL tasks mainly focus on language comprehension at the literal level, emphasizing the need to add inferential language comprehension test batteries to the research protocol. Last, researchers must take into account and tackle the possibility that children with lower language levels might show less motivation in storybook interventions than their typically developing peers.”

21-ii) Discuss if there were elements in the RCT that would be different in a routine application setting

Discuss if there were elements in the RCT that would be different in a routine application setting (e.g., prompts/reminders, more human involvement, training sessions or other co-interventions) and what impact the omission of these elements could have on use, adoption, or outcomes if the intervention is applied outside of a RCT setting.

	1	2	3	4	5	
subitem not at all important	○	○	○	◉	○	essential

Selectie wissen

Does your paper address subitem 21-ii?

Copy and paste relevant sections from the manuscript (include quotes in quotation marks "like this" to indicate direct quotes from your manuscript), or elaborate on this item by providing additional information not in the ms, or briefly explain why the item is not applicable/relevant for your study

Given that research questions in the current study were not based on a randomized controlled trial design, we did not discuss subitem 21-ii in the current manuscript. However, when investigating the boosting effect of the EE algorithm in a future study, this aspect will be taken into account.

OTHER INFORMATION

23) Registration number and name of trial registry

Does your paper address CONSORT subitem 23? *

Copy and paste relevant sections from the manuscript (include quotes in quotation marks "like this" to indicate direct quotes from your manuscript), or elaborate on this item by providing additional information not in the ms, or briefly explain why the item is not applicable/relevant for your study

The trial registration number is provided in the manuscript: "Following a school-based screening in the third year of kindergarten (n=1225), 149 five-year old children (119 children with and 30 children without an elevated cognitive risk for dyslexia) enrolled in a game-based preventive reading intervention study (Trial registration number: S60962 – assigned by the Clinical Trial Center of UZ Leuven, Belgium)."

24) Where the full trial protocol can be accessed, if available

Does your paper address CONSORT subitem 24? *

Cite a Multimedia Appendix, other reference, or copy and paste relevant sections from the manuscript (include quotes in quotation marks "like this" to indicate direct quotes from your manuscript), or elaborate on this item by providing additional information not in the ms, or briefly explain why the item is not applicable/relevant for your study

The full protocol is not online available, though available upon reasonable request.

25) Sources of funding and other support (such as supply of drugs), role of funders

Does your paper address CONSORT subitem 25? *

Copy and paste relevant sections from the manuscript (include quotes in quotation marks "like this" to indicate direct quotes from your manuscript), or elaborate on this item by providing additional information not in the ms, or briefly explain why the item is not applicable/relevant for your study

Funding has been described in the acknowledgement section of the manuscript as follows: “This study obtained funding from the Research Council of KU Leuven, Belgium (C14/17/046). Co-author Jolijn Vanderauwera was a postdoctoral researcher and received a personal grant of the Research Foundation Flanders (FWO), Belgium (12T4818N).”

X27) Conflicts of Interest (not a CONSORT item)

X27-i) State the relation of the study team towards the system being evaluated

In addition to the usual declaration of interests (financial or otherwise), also state the relation of the study team towards the system being evaluated, i.e., state if the authors/evaluators are distinct from or identical with the developers/sponsors of the intervention.

	1	2	3	4	5	
subitem not at all important	○	○	○	◉	○	essential

Selectie wissen

Does your paper address subitem X27-i?

Copy and paste relevant sections from the manuscript (include quotes in quotation marks "like this" to indicate direct quotes from your manuscript), or elaborate on this item by providing additional information not in the ms, or briefly explain why the item is not applicable/relevant for your study

Conflicts of interest were stated in the manuscript as follows: “This study was conducted without any conflicts of interest (“none declared”).”

About the CONSORT EHEALTH checklist

As a result of using this checklist, did you make changes in your manuscript? *

- ○ yes, major changes
- ○ yes, minor changes
- ◉ no

What were the most important changes you made as a result of using this checklist?

No changes were made since the manuscript was already accepted fur publication in JMIR Serious Games by the time we filled out the Consort Ehealth checklist.

How much time did you spend on going through the checklist INCLUDING making changes in your manuscript *

We have spent 20 hours on going through the checklist.

As a result of using this checklist, do you think your manuscript has improved? *

- ( ) yes
- ( ) no
- (•) Anders: No, since no changes were made (manuscript already accepted for p

Would you like to become involved in the CONSORT EHEALTH group?

This would involve for example becoming involved in participating in a workshop and writing an "Explanation and Elaboration" document

- ( ) yes
- (•) no
- ( ) Anders:

Selectie wissen

Any other comments or questions on CONSORT EHEALTH

Jouw antwoord

STOP - Save this form as PDF before you click submit

To generate a record that you filled in this form, we recommend to generate a PDF of this page (on a Mac, simply select "print" and then select "print as PDF") before you submit it.

When you submit your (revised) paper to JMIR, please upload the PDF as supplementary file.

Don't worry if some text in the textboxes is cut off, as we still have the complete information in our database. Thank you!

Final step: Click submit !

Click submit so we have your answers in our database!

Verzenden Formulier wissen

Verzend nooit wachtwoorden via Google Formulieren.

Deze content is niet gemaakt of goedgekeurd door Google. Misbruik rapporteren - Servicevoorwaarden - Privacybeleid

Google Formulieren
