# Supplementary material for: Feasibility, Enjoyment, and Language Comprehension Impact of a Tablet- and GameFlow-Based Story-Listening Game for Kindergarteners: Methodological and Mixed Methods Study
Source: JMIR Serious Games. 2022 Mar 23;10(1):e34698. doi: 10.2196/34698 (PMC8987971; doi:10.2196/34698)
Supplement: Multimedia Appendix 2 [file games_v10i1e34698_app2.pdf]

# Multimedia Appendix 2: Technical story game development guide

## Short game description and rationale

The story game is a tablet application, originally developed to test the efficacy of listening to envelope enhanced speech (EE) on phonology and reading via improved basic auditory speech processing among five-year old children at cognitive risk for dyslexia. The EE algorithm automatically detects and amplifies important rhythmic acoustic cues of the speech envelope in digital audio recordings, i.e., onset rise times [47-51], which are considered to be important for phonological skill development [52,53]. The story game mainly consists of a main intervention task, i.e., listening to Dutch-spoken recorded age-appropriate children stories, followed by a story rating part and a short quiz with basic content-related questions. In total, the game provides 72 story sessions, categorized in 18 game phases of four grouped sessions containing four (longer) to eight (shorter) stories from the same book series and author. The game facilitates rewards in the form of stars merely for the full completion of a game session and in the form of coins for responding correctly to the content-related questions. Players can spend their earned coins in a virtual shop, i.e., the avatar customization system. The shop items customizes the players' avatars, who move around a map, i.e., the virtual hub world, and inform players on their game progress. The game is suitable for Android Devices (in the case of the current study: Samsung Galaxy Tabs E9.6). Since the game is intended for five-year-old children, it has been developed as functionally simple as possible. For instance, it does not offer any form of written text and the players are only required to listen to spoken text and press buttons which appear vividly on the screen.

## Game development

## Pre-programming phase

Prior to the programming phase, a story game concept, which consisted of game-based story listening phases, was created by different members of the research group, taking into account the elements of the GameFlow model (except for the 'social interaction' element) [5]. The reason for not including the social interaction element was driven by the fact that the participants of the current study were too young for between-player interactions (e.g., via chat boxes) and that all participants were anonymously and independently recruited, hindering communication between different players. After finalizing the concept, several children storybooks were screened via library and publisher visits. Storybooks were eligible for the story game (1) if they were part of a book series that contained minimally three other published books, (2) when their target age was four to six years old, and (3) if they lasted approximately five to fifteen minutes. The limitation in length was set based on similar story lengths in previous kindergarten story listening studies [64, 65] and because of the feasibility for young children to combine the story game with the other intervention games for 12 weeks on a nearly daily basis. Based on these criteria, we selected 87 storybooks from 14 different book-series, for which all publishers sent written consents via email to use the book texts and images within the framework of the intervention study (see Supplementary Table 1 for an overview of the game phases and their corresponding book series, authors, illustrators, and publishers). For each story session, the research group created three content-related multiple-choice questions with three response alternatives each. All selected stories were then recorded by nine different highly skilled female speakers with knowledge of Standard Dutch in Flanders. Female voices were opted for since a previous study showed better learning outcomes in female-instructed digital problem-solving tasks and therefore advised the use of female voices when designing multimedia learning environments [66]. Yet, to bring some variation into the speakers' voices during the game sessions, the game instructions and

content-related questions and their corresponding multiple-choice alternatives were recorded by a male Flemish radio presenter and a female eight-year old child respectively. Next, all recordings were edited to exclude errors and slip of the tongues. The envelope enhancement on the story recordings for the GG-FL_EE group was done by a signal processing expert and is fully described in the study of Van Herck and colleagues [49]. For each story, the corresponding story illustrations from the book were linked to a specific timeframe of the audio recording and summarized in so-called audiovisual-synchronization documents. Incorporating images along with the story recordings was based on the idea that illustrations benefit narrative comprehension [64, 67] and foster engagement [63]. The story recordings, illustrations and their accompanying question recordings and audiovisual-synchronization information were ordered in a predetermined file structure (see rationale and clarification of this file structure in the section on the programming details of the main intervention task environment).

Supplementary Table 1. Book series, authors, illustrators, and publishers of the stories implemented in the story game.

| Game phase | Session | Book series | Author(s) | Illustrator | Publisher | Amount of stories |
|---|---|---|---|---|---|---|
| 1 | 1 – 4 | Lotta de Kip | Diane Put, Rik De Wulf | Rik De Wulf | Clavis | 8 |
| 2 | 5 - 8 | Ella het heksje | Guy Daniëls | Lisa Brandenburg | Clavis | 4 |
| 3 | 9 – 12 | Avonturen van Tommie en Lotje | Jacques Vriens | Kees de Boer | Van Holkema& Warendorf | 4 |
| 4 | 13 – 16 | Joep en Pleuntje 1 | Isabelle De Ridder | Monique Dozy | Clavis | 4 |
| 5 | 17 – 20 | Winnie de Poeh | A.A. Milne | Ernest H. Shepard | Deltas | 4 |
| 6 | 21 – 24 | Het Winkelstraatje | Marianne Busser, Ron Schröder | Ingrid ter Koele | Moon | 4 |

| | | | | | | |
|---|---|---|---|---|---|---|
| 7 | 25 – 28 | Verhalen uit het Elzen-Eikenbos | Fiona Rempt, Noelle Smit | Noelle smit | Van Holkema& Warendorf | 4 |
| 8 | 29 – 32 | Beroepenserie 1 | Liesbet Slegers | Liesbet Slegers | Clavis | 6 |
| 9 | 33 – 36 | Rikki | Guido Van Genechten | Guido Van Genechten | Clavis | 8 |
| 10 | 37 – 40 | Grote Anna 1 | Kathleen Amant | Kathleen Amant | Clavis | 4 |
| 11 | 41 – 44 | Meneer Big | Gitte Spee | Gitte Spee | Pimento | 4 |
| 12 | 45 – 48 | Joep en Pleuntje 2 | Isabelle De Ridder | Monique Dozy | Clavis | 4 |
| 13 | 49 – 52 | Beroepenserie 2 | Liesbet Slegers | Liesbet Slegers | Clavis | 7 |
| 14 | 53 – 56 | Grote Anna 2 | Kathleen Amant | Kathleen Amant | Clavis | 4 |
| 15 | 57 – 60 | Hekselien | An Melis | An Melis | Abimo | 4 |
| 16 | 61 – 64 | Sjon en Sjaan | Gitte Spee | Gitte Spee | Pimento | 4 |
| 17 | 65 – 68 | Beroepenserie 3 | Liesbet Slegers | Liesbet Slegers | Clavis | 6 |
| 18 | 69 - 72 | Lars de kleine ijsbeer | Hans De Beer | Hans De Beer | Vier Windstreken | 4 |

## Scripts

The story game was entirely programmed in Unity 3D [62] using the C# scripting language. Most of the inner workings of the game were detailed in appropriately named scripts. The scripts could generally be divided into four categories (see Supplementary Table 2 for an overview of all scripts implemented in the game):

- **Component scripts** handled specific situations, such as the shop in the avatar customization system, the avatar, the quiz, the ending animation, etc. They were generally used in one specific situation in the game.
- **Global scripts** persisted between different game phases (virtual hub world, main menu, shop in avatar customization system, and questions). Each script handled features that needed to be called in different situations spread throughout the game, i.e., saving, playing music, and playing instruction sounds.

- **Utility scripts** provided functions that were not specific to the game, but that were generally useful. For example, it included a script for orbiting the camera with touch.
- **Editor-specific scripts** existed to facilitate the game development phase and were not included in the game builds. They consisted of helper codes that did not run on the tablets of the participants, but solely on the computer of the game developer.

Supplementary Table 2. Scripts implemented in the story game

| Script | | Description |
|---|---|---|
| **Global scripts** | | |
| | Global | This was an overall manager script, providing connections to all existing global scripts, facilitating loading, playing music, etc. |
| | Calibrator | Handled sound calibration user interface and applied those settings to the Unity Audio Mixer system. |
| | CharacterGenerator | Used saved settings to generate an instance of the customizable players' avatars. |
| | Configurator | Handled game configuration, i.e., saving, loading profiles, and changing profile variables such as the maximum number of sessions per 24 hours. |
| | Instructor | Played instruction audio, provided helper functions to select appropriate instruction audio. |
| | LevelData | Determined the colors of stars and stages in the different game phases. |
| | Music | Played and sustained music clips depending on the game context. Allowed sound to transition smoothly. |
| | SaveData | Saved and loaded profile specific data, such as stars, coins, unlocked levels, and equipment. |
| **Components scripts** | | |
| | Camera Movement | Handled camera movement in the virtual hub world. |
| | Character | Controlled single avatars: their equipment, animations, and movements around the virtual hub world. |
| | EndingScene | Used to play the ending animation in the hub world, when a player finished the entire story game. |
| | MainMenu | Controlled user interface of the main menu. |

| | | |
|---|---|---|
| | Map | Directed the game during the virtual hub world mode. Received player input and triggered transitions and animations. |
| | Quizmaster | Directed the game during the quiz mode. Guided question audio, buttons, animations, and transitions. |
| | StoryTeller | Directed the game during story listening mode. Guided story audio, panels, pausing, and triggered the quiz when the story was finished. |
| | Shop | Directed the game during the avatar customization mode. Guided buying of items, sound effects, music playing, and avatar swapping. |
| | ShopDisplayItem | Controlled props in the avatar customization system background, which appeared when the player was able to buy new items from the shop. |
| | ShopItem | Controlled a single user interface in the shop that could be unlocked, bought, and equipped. It included characters and accessories. |
| | StoryHolder | Held a set of stories, including the associated audio, questions, image panels, timings. |
| **Utility scripts** | | |
| | CameraOrbit | Orbited the camera around a fixed point based on the touch input. This only occurred in the avatar customization system. |
| **Editor-specific scripts** | | |
| | objExporter | Exported a Unity 3D model to a standardized 3D model format (.obj), which facilitated editing the models with other software. |
| | AutoLevel | Allowed the developer to directly end up in the avatar customization system, question part, or virtual hub world, without having to pass through the main menu. |
| | Recenter | Used to automatically reset positions of many objects, i.e., the animation models in the virtual hub world, facilitating positioning of different animations. |
| | StoryLoader | Loaded story data from the Stories folder and exported the data to Storyholder objects for easy access. |

## Specific design and programming details

## Main intervention task environment

### *Design details*

In the main intervention task environment, players were required to listen to one or two short stories, followed by a story rating and content-related questions. The story and accompanying imagery played on a mostly neutral black screen to lay focus on the content of the story itself. The story was paused at key moments, while displaying a large green play button to continue. This allowed a short break during the listening phase and ascertained that the player continued to pay attention to the story, since there was no option to rewind. While the recording played, images smoothly faded in and out. Once a single story finished, the story rating part and quiz occurred. Each time the question audio of the quiz was played, buttons representing the response options appeared on the screen. The response audio was played one by one, while highlighting each individual corresponding button. Once the response audio was finished, an extra replay button appeared to play the question and the response audio again, using the same animations. The buttons became intractable once the first round of audio was complete. The players immediately received feedback whether or not their answer was correct. A correct answer resulted in a short animation showing clearly that the player had earned a new coin. A wrong answer resulted in a short animation which revealed the correct response button. The response buttons had a simple design, based on the star theme of the game and showed either one, two, or three dots, representing response option one, two, or three respectively.

### *Programming details*

#### Story implementation

The stories in the game were subject to a fairly complex data structure, due to limitations of memory on Android. The story audio and accompanying images, the

audiovisual-synchronization document, the question and response audio were ordered in a predetermined file structure and implemented in the game based on a specific format. This predefined file structures could automatically be loaded into the Unity editor based on the "*StoryLoader*" script. Thus, it accelerated the design process as it allowed to quickly implement updates regarding audiovisual synchronization documents, pauses, and questions, without extra specialized programming work. The main folder was named "*Stories*" (left panel of Supplementary Figure 1) and contained 18 sub-folders, representing the different story themes of the 18 game phases (for example, the folder of game phase 9 with stories of Rikki the Rabbit was named "*Level_09_Rikki*"). Each game phase folder was further subdivided in four sub-phase folders (e.g., "*L09_S01*", "*L09_S02*", "*L09_S03*", "*L09_S04*", each containing a set of stories (either one long story or two short ones) the player should listen to in one game session (right panel of Supplementary Figure 1). In total, 72 sub-phase folders were created, e.g., four for each game phase, representing the total amount of 72 game sessions. When a sub-phase contained two short stories instead of one long, the folder was further subdivided into separate story folders, such that each story had its own folder (e.g., "*L13_S02_01"* and "*L13_S02_02"*) (Supplementary Figure 2). In total, 87 story folders were created, since a total of 87 stories were implemented in the game.

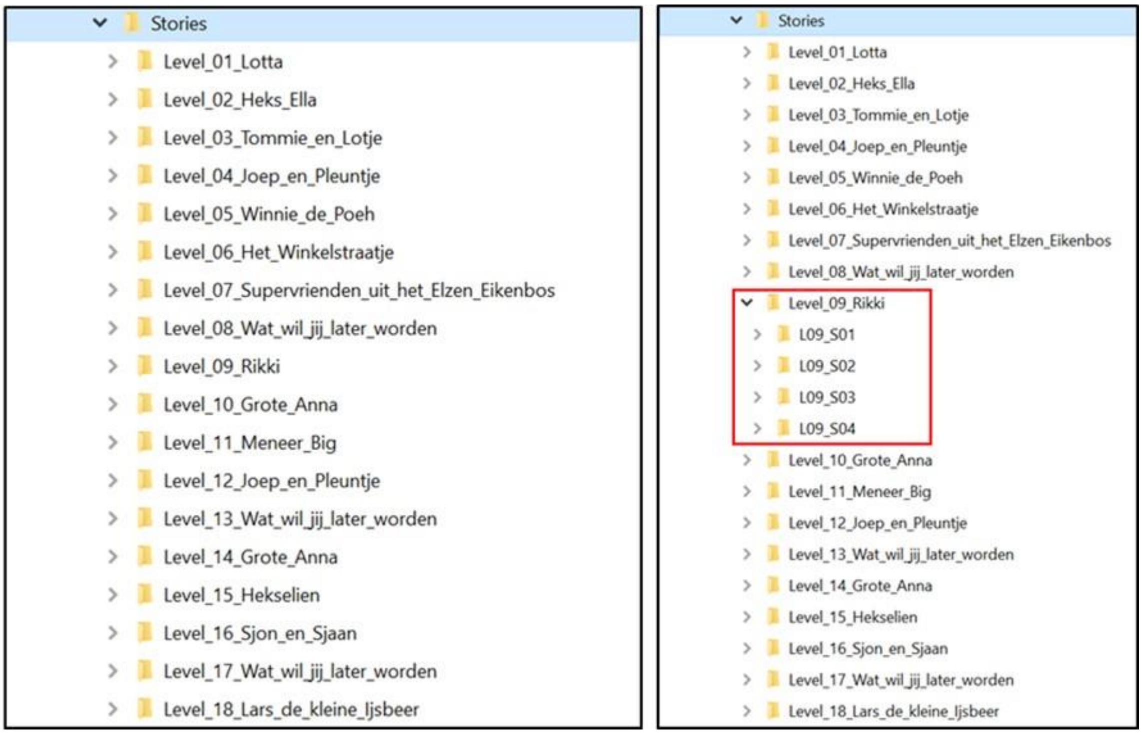


*Supplementary Figure 1*. File structure of the stories of the story game. Left panel) Overall game phase folders. Right panel) Sub-phase folders

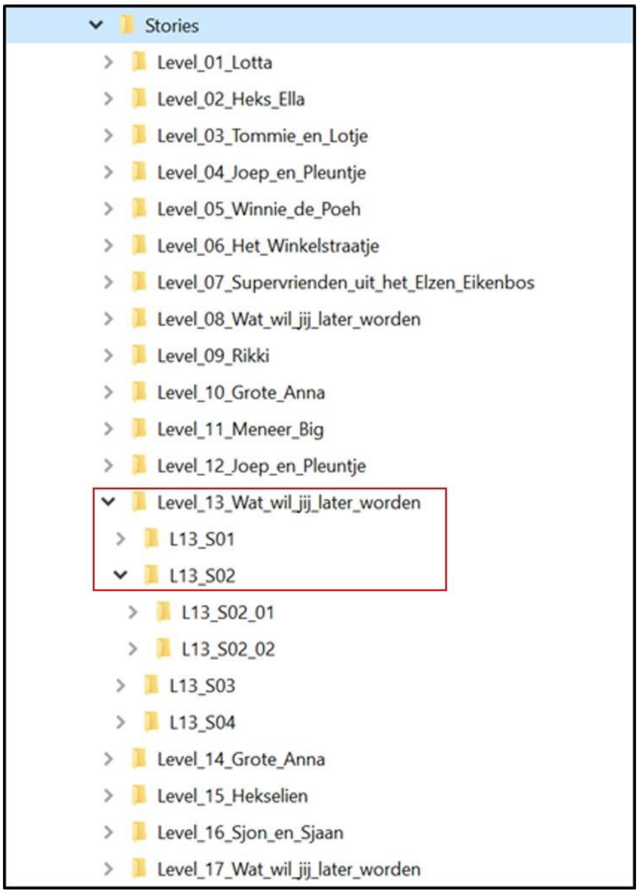


*Supplementary Figure 2*. Example of a sub-phase folder (*L13_S02*) containing two separate story folders (*L13_S02_01 and L13_S02_02*).

Each of the 87 separate story folders contained three types of subfolders (Supplementary Figure 3):

- "*audio*" folder, containing the story recording in .wav format.
- "*afbeeldingen*" folder, consisting of (1) the set of images in .jpg format to be displayed during the story and (2) an audiovisual-synchronization .txt document (see detailed audiovisual synchronization section below). Each image was named numerically as "*afbxx.jpg*". The .jpg format was preferred since it saved memory.
- *"vraagjes"* folder, containing the question and response recordings in .wav format along with a .txt document which specified the order of the questions and corresponding response alternatives (see detailed questions section below).

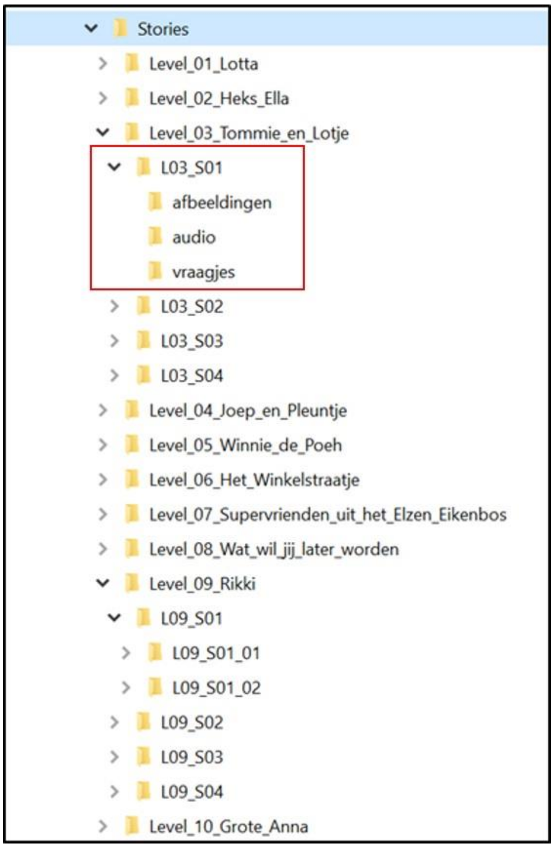

*Supplementary Figure 3*. The data structure of a story folder

The editor-specific "StoryLoader" script provided functions for importing data in the main "*Stories*" folder and sorting them into prefabs that the game used to load the story data.

This setup was necessary, since otherwise the programmer was forced to load all of the story data at once to play a single story. The parsing assumed that the naming conventions outlined previously were being upheld. As such, everything could be imported, but also easily updated if necessary without issues.

### Audiovisual synchronization

Each “*afbeeldingen*” folder of a story contained a so-called audiovisual-synchronization file in .txt format (see Supplementary Figure 4 for an example). It defined when image panels accompanying the story audio had to be shown. The file contained a list of the times at which the panel should show a particular image, specified as “*afbxx_mmss*”. The time was thus listed as a minutes-seconds format. For instance, the line “*afb09_0105*” in Supplementary Figure 4 meant that the image ’afb09.jpg’ was shown at minute 1 and 5 seconds, or 65 seconds throughout the story. Timing regulations were programmed as follows: one second before a specific timing indication, the previous image panel faded out, at the timing per se, a black screen was shown, and one second after the timing, the image accompanying the specific timing appeared on the screen. When a timing indication was followed by the text “(*hoeft niet*)”, the given image was ignored. A “(*pause)*” specification indicated that the given transition should also pause the story, prompting the player to continue it manually by pressing on the green arrow. If a given list of timings did not have any *“(pause)”* flags, the story paused after every single image by default.

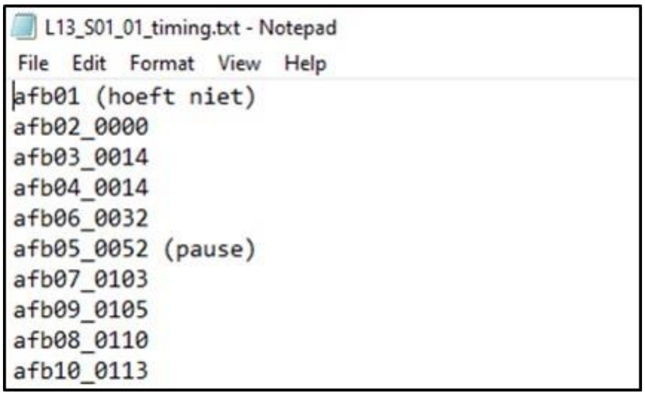

L13_S01_01_timing.txt - Notepad

File Edit Format View Help

```
afb01 (hoeft niet)
afb02_0000
afb03_0014
afb04_0014
afb06_0032
afb05_0052 (pause)
afb07_0103
afb09_0105
afb08_0110
afb10_0113
```

*Supplementary Figure 4.* Example of an audiovisual synchronization document.

Questions

Each “*vraagjes*” folder contained a document which specified the order of the questions for the particular story and their corresponding response alternatives. It was consistently named as “*Storyname_vragen.txt*” (e.g., *L03_S01_vragen.txt* for the questions of the story in the first sub-phase of the third game phase) (Supplementary Figure 5). This file first specified the name of the question audio (e.g., L03_S01_vraag01 is the first question for the story), followed by a written example of the actual question and three possible answers, rounded off by a white line. Specifying the text content of the question and responses in these files was not really relevant to the game, but it was used for data exportation on the accuracy of the questions. For each question, the correct answer was marked using the keyword *“(juist)”*. The relevant audio corresponded the question name as specified in the question files. For instance, the question *“L03_S01_vraag01”* had an audio file which was named *“L03_S01_vraag01.wav”*.

```
L03_S01_vragen.txt - Notepad
File Edit Format View Help
L03_S01_vraag01
Waarom moet Lotje in bed blijven?
Ze is stout geweest
Ze is ziek (juist)
Het is midden in de nacht

L03_S01_vraag02
Waarom is Lotje verdrietig?
Ze heeft honger
Ze heeft eng gedroomd
Ze mag niet in de sneeuw spelen van mama (juist)

L03_S01_vraag03
Wat zit er in de doos van Tommie?
Een hoopje sneeuw (juist)
Bloemen
Steentjes
```

*Supplementary Figure 5.* Example of a question file. L03_S01_vragen.txt

Virtual hub world

*Design details*

The virtual hub world showed a visual overview of the current story theme (e.g., one of the 18 game phases) as well as the overall progress of the player. A customizable avatar jumped from one cylindrical stage to the next, when finishing a story session. Each stage thus represented one story session. The virtual hub world was shown in a restrictive orthogonal perspective, which revealed only a handful of stages at a time. The reasoning behind this was to create anticipation towards what appeared next on the map and to motivate players to come back to see what would be revealed next. The virtual hub world was designed in a way that the art work matched the content of the stories (e.g., a chicken coop representing the home of the main character in the four story sessions with books of “Lotta De Kip”/ “Lotta the Chicken”, as shown in the upper panel of Supplementary Figure 6). When a story became available, a big green flashing button appeared on the screen, inviting the player to listen to it. The hub world also contained the ending animation, which was shown when all game sessions were completed. It brought together all the elements of the game, including all possible game

avatars and one star from each of the 18 game phases (see lower panel of Supplementary Figure 6).

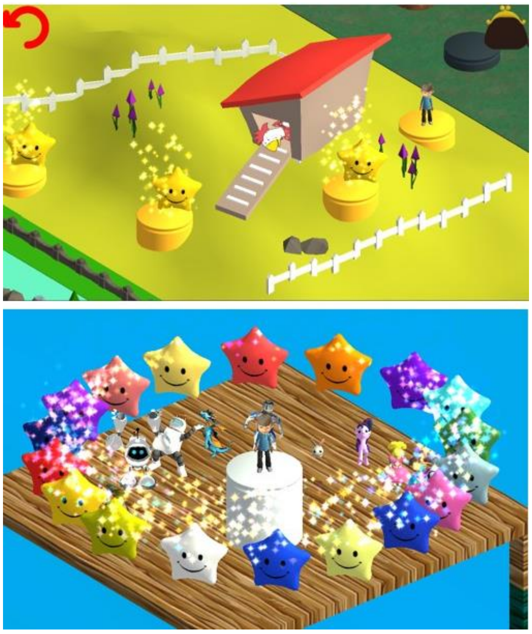

*Supplementary Figure 6.* Upper panel) Alignment of the hub world with the themes of the story series. Lower panel) Ending cut-scene in the hub world.

*Programming details*

Supplementary Table 3 provides an overview of the art work (props) implemented in the virtual hub world and their corresponding sources.

Supplementary Table 3. Props and their sources implemented in the virtual hub world.

| Props | Source |
|---|---|
| Forests | Unity Asset Store: Free low Poly Nature Pack [68] |
| Fall forest & woodland textures | Unity Asset Store: Low Poly Nature Pack Lite [69] |
| City buildings | Recycled from Diesel-X [58] |
| Spooky mansion | Sketchfab: World Skills Spooky Mansion by Philip Osborne [70] |
| Starting area | Unity Asset Store: Low Poly Style Environment [71] |

Avatar customization system

*Design details*

After the main intervention task (e.g., listening to one or two stories, the story rating, and the short content-related quiz), players automatically ended up in a so-called avatar customization system, where they could spend earned coins on new avatars and/or accessories for their existing avatars in a little shop. The ten possible avatars (i.e., young boy/girl, robot, dragon, farm animal, pony, popstar, princess, knight, American Indian, or astronaut) were not directly linked to the story contents of the game, but they had been selected based on (1) their attraction both for boys and girls, (2) the ease for game implementation, and (3) the possibility to deck them out with plenty of accessory options. Each avatar could be unlocked based on a certain amount of earned coins and had eight distinct accessories that could be equipped simultaneously. Once players bought avatars or accessories, it was displayed on the screen. Two types of avatars were considered as special cases. First, as from the start of the intervention, the young boy or girl avatar (depending on the gender of the player for which an account was created) was set as the default initial avatar. Second, the farm animal used its accessory options to swap the current farm animal model for a different animal. It also had more options than any other avatar. Prices for all the avatars and accessories were set so that a perfect player could still spend all of his/her coins in the avatar customization shop.

*Programming details*

The code for implementing avatars was streamlined as much as possible, so that new avatars could be added to the game with only little extra programming effort. All avatars were 3D models (either custom-made, acquired from Sketchfab, or the Unity asset Store) with textures (coloration) and rigging (tools which allowed the avatar to move in the game). For each avatar, the following properties were separately programmed:

- An animation system, which allowed different movements of the avatars, containing four possible animations:
    - Idle: looping animation that played by default when the avatar was in a resting state (e.g., when showed in the main menu)
    - Jump: played when a jump from one cylindrical stage to the next started. The animation played in reverse when the jump ended and the avatar landed.
    - Airborne: looping animation that played in the middle of a jump
    - StarPose: animation that played directly after a new star appeared on the cylindrical stage that was just abandoned.
- A shop entry for the avatar, which included its pricing and a 256x256 preview image
- A set of accessories connected to the avatar. For each accessory, two extra properties were programmed.
    - A shop entry for each unlockable accessory, which included its pricing and a 100x100 preview image
    - An optional ’ShopDisplayItem’. This was a non-functional version of the item that was visible in the shelves of the shop once it had been unlocked.

Supplementary Table 4 presents a list of the ten avatars, their accessories, and their sources. Supplementary Table 5 provides an overview of all sources of the available accessories. Accessories not mentioned in Supplementary Table 5 were custom made for the project.

Supplementary Table 4. Avatars and sources

| Avatar | Accessories | Source |
|---|---|---|
| Boy/girl (default) | Propeller or sun hat, backpack, skates, special outfit, other hair color, party hat, swimming floaty, outfit change | Unity Asset Store [72] |

| | | |
|---|---|---|
| Robot | Red paint, happy face, angry face, jetpack fire, mini robot, surprised face, shield bubble, blue paint | Unity Asset Store [73] |
| Astronaut | Green paint, robot arm, satellite dish, laser sword, star badge, screen on belly, rocket boots, star necklace | Custom made |
| Dragon | Red dragon, white dragon, propeller hat, winged shoes, mini dragon, dog collar, big wings, saddle | Unity Asset Store [74] |
| Knight | Sword, shield, helmet plume, crown, sparkles, red armor, green armor, star necklace | Unity Asset Store [75] (not available online anymore) |
| Farm animals | Variations in animal models act like accessories: duck, chicken, cat, dog, pig, sheep, goat, horse, lama, cow | Unity Asset Store [76] |
| Pony | Horn, open wings, sparkles, red pony, rainbow pony, star necklace, rainbow bracelets, mini pony | Sketchfab [77] |
| Princess | Skirt ribbon, rose, fairy hat, crown jewel, magic wand, blue outfit, star necklace, sparkles | Custom made |
| Popstar | Sunglasses, microphone, new guitar, flying speakers, rainbow spotlight, red outfit, black outfit, sparkles | Custom made |
| Native American | Feather, chieftain hat, bow, necklace, parrot, dream catcher, quiver, outfit change | Custom made |

Supplementary Table 5. Accessories and sources

| Accessory | Source |
|---|---|
| Backpack | Included in asset pack of boy/girl avatar |
| Knight crown | Sketchfab |
| Princess crown | Sketchfab |
| Dream catcher | Sketchfab |
| Swimming floaty | Sketchfab |
| Fairy hat | Sketchfab |
| Knight sword | Separated from base model |
| Knight shield | Separated from base model |
| Laser sword | Sketchfab |
| Mini robot | Sketchfab |
| Sun hat | Included in asset pack of boy/girl avatar |
| Quiver | Sketchfab |
| Rose | Sketchfab |
| Pony horn | Separated from base model |
| Pony open wings | Separated from base model |

Additional game features

*Main menu*

The main menu of the story game appeared as a static scene of a bedroom and menu elements were designed into a book, to imitate a bedtime story moment (Supplementary Figure 7). The artwork of the fairytale book and bedroom elements were acquired from Sketchfab. The main menu showed the active player account and the amount of total earned stars to inform the player on the progress throughout the game. The settings system, represented by the gear wheel icon, was locked with a password to prevent regular users from accessing it. A game session began by pressing on the large green arrow. To exit the game, players were required to press the red arrow.

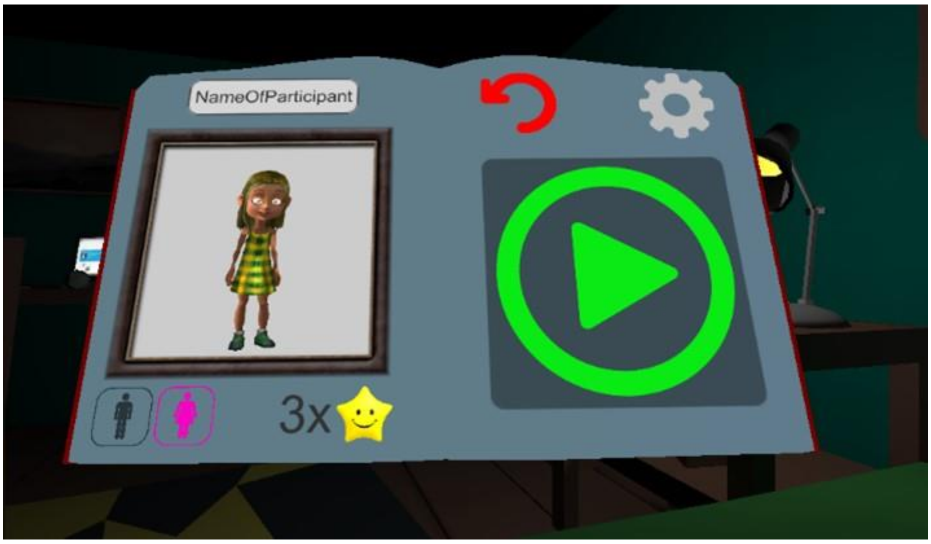


*Supplementary Figure 7.* Main menu of the story game.

*Settings*

Sound-related settings

Sound-related settings included options to calibrate the sound level of the stories in the game, and adjust the sound level of the sound effects, instructional voices, and music tracks (see Supplementary Figure 8). The 'Device volume' slider controlled the Android volume setting which could otherwise be adjusted via the buttons on the side of the tablet. This input was restricted during story game play to make sure players could not interfere with the

calibration. The other three sliders controlled gain values in dB for instruction speech (cfr. Instruction gain), music (cfr. Music gain) and sound effects (cfr. SFX gain) respectively. These were set by default to an agreed upon level, but could be adjusted if necessary. The calibration panel was used for calibrating the sound levels of the stories in the game. Story sound levels were calibrated to be played at 60 dB-A (see "*Experiment level*" box in Supplementary Figure 8). Calibration was performed in a sound proof booth by playing speech weighted noise that was constructed based on the average spectra of the nine different female story tellers at 90 dB-A (i.e., see "*Calib target level box*" in Supplementary Figure 8) through a headphone (ATH m20x). The measured output level was then filled in in the "*Measured level*" box and the resulting gain, specified in the "*Current gain"* box was automatically applied to the story recordings, but also to the instruction, music and sound effect gains. Calibration was iterated as long as necessary. The sample sounds panel allowed to play example recordings of the story speech sound (i.e., speech sample in Supplementary Figure 8), sound effects (SFX sample in Supplementary Figure 8), and music (Music sample in Supplementary Figure 8) to check whether the applied gains after calibration were acceptable. It also provided short audio recordings of white noise and the speech weighted noise, which was used for calibration of the story sound levels.

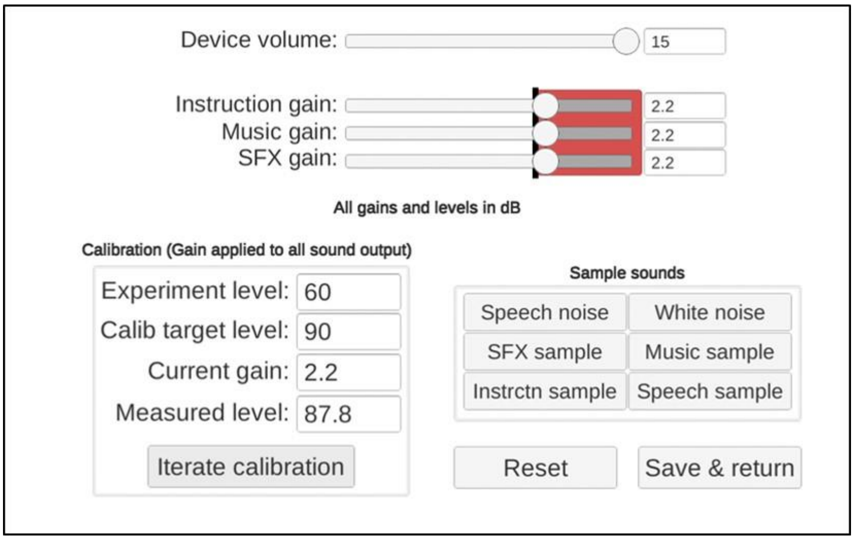


*Supplementary Figure 8*. Sound-related settings

## Game configuration settings

The game configuration settings (Supplementary Figure 9) offered a series of settings that could be used to adjust a certain game account behavior (consider Supplementary Table 6 for an overview of the different game settings). Game configuration settings also included panels to add or remove player accounts. Each account was recognized based on a name and ID number (e.g., Name-01 as specified in Supplementary Figure 9). The name was displayed to the player in the main menu, while the ID number was used internally by members of the research group to recognize and differentiate large numbers of test subjects. Experimental results of each account (e.g., play dates and hours, game progress, question response accuracy, and story ratings) were saved as binary .sav files. On Android, such files were stored in the data folder, under the package name for the game, which was set as "*be.kuleuven.med.exporl.InterventionGame*" in the current intervention study. Manually exporting the data (as specified by the "*Export now*" button in Supplementary Figure 9) made sure that the .sav files were stored in an easily accessible folder on the Android device, which was created by members of the research group and for which its location was specified by the path in the configuration panel. Experimental play data were also converted in a user-readable format, i.e., a text file (.txt) for each individual player containing detailed player information, and a simplified csv file (.csv) containing more general play data.

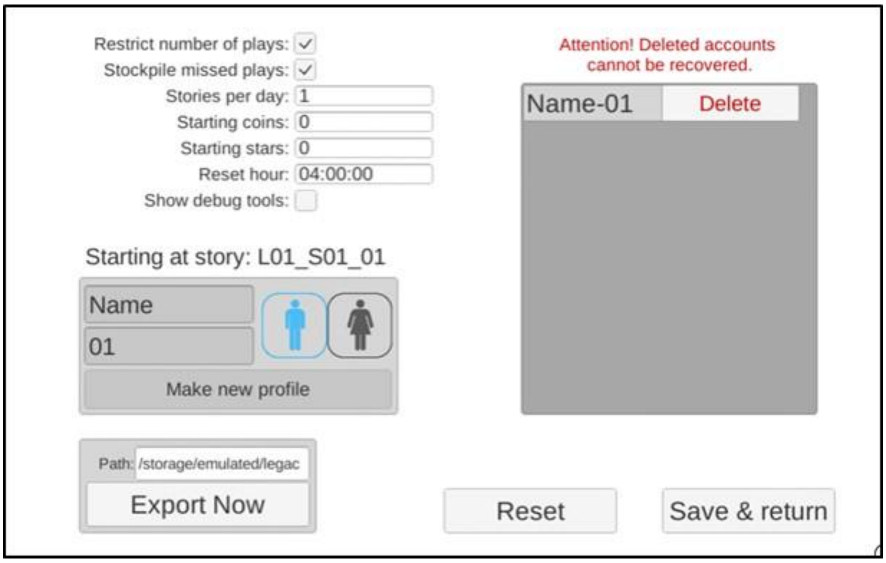

*Supplementary Figure 9.* Game configuration settings

Supplementary Table 6. Available game configuration settings

| Setting name | Purpose |
|---|---|
| Restrict number of plays | Blocked player from playing more than one game session per 24 hours. |
| Stockpile missed plays | Allowed players to catch up if they forgot to play a session, e.g., they could play three story sessions in a row if they forgot two days of playing. |
| Stories per day | The amount of story sessions that could be played per day. Note that “1” was set as default in the intervention study. |
| Starting coins | The amount of coins that were initially given to a newly created profile. This setting did not affect existing profiles. It was intended for debugging purposes and was set as “0” in the current intervention study. |
| Starting stars | The amount of stars that were initially given to a newly created profile. This setting did not affect existing profiles. It was intended to let players start at different game sessions in the game, but it was also useful for debugging purposes. In the current intervention study, it was always set as “0” so that every player started with the first story of the first sub-phase of the first game phase. |
| Reset hour | The hour at which the restriction counting was triggered. For instance, if this value was set as 04:00:00, the player was blocked from playing a second game session until 4 AM. |

| | |
|---|---|
| Show debug tools | Displayed tools for fast forwarding and skipping trough stories. It was useful for faster debugging, but was turned off in the current intervention study. |

*Music and sound effect implementation*

Music tracks implemented in the game were royalty free music written by Kevin Macleod [78] (see Supplementary Table 7 for the track titles and the occasion when they occurred in the game). Sounds effects where acquired from a freely accessible website and edited where necessary [79]. Supplementary Table 8 provides an overview of the different sound effects and their occurrences in the game.

Supplementary Table 7. Track names and occurrence in the story game.

| Track name | Occurrence in game |
|---|---|
| Melodie Victoria | Main menu |
| Carefree | Virtual hub world |
| Cheery Monday | Virtual hub world |
| Teddy Bear Waltz | Virtual hub world |
| Zazie | Avatar customization system |

Supplementary Table 8. Sound effects and their occurrence in the story game.

| Sound effect | Occurrence in the game |
|---|---|
| Button | Occurs when a player presses on the green arrow to start the game session in the main menu |
| Buy | Occurs when a player buys a new avatar or accessory |
| Chime | Occurs when a new story starts |
| Coin | Occurs when opening the avatar customization system. It represents the sound of falling coins |
| Correct | Occurs when the answer to a content-related question is correct |
| Equip | Occurs when an accessory is applied to an avatar |
| Error | Occurs when a player tries to buy accessories/avatars without enough coins |
| Select | Occurs when avatars change |
| Unequip | Occurs when an accessory is removed from the avatar |
| Wrong | Occurs when the answer to a content-related question is incorrect |

*Exporting data experimental results to server*

Experimental results (e.g., the generated .txt and .csv files for each account) were automatically logged to a University internet server, so that data were backed up during the home-based intervention and members of the research group could keep track of the intervention trajectory of all participants for a longer period of time. Collecting and synchronizing these experimental results was handled by an external so-called Android AutoSyncer application, which was specifically developed for the current intervention study and installed on all tablets. The AutoSyncer application could communicate with a basic server over the internet to send data files and sort them into a predefined data structure, thus maintaining in-game player data from many different users at the same time.

# Bugs

## Failure to delete active profiles

When a player exited the game via the intended red arrow in the main menu, the game saved the currently active profile one last time. Yet, if that active profile had been previously deleted for some reason in the main configuration system, it would just be rewritten back, making it seemingly impossible to delete that profile. This bug could be circumvented by closing the game externally, for example via the Android task manager, yet it should be fixed in future versions of the story game.

## Blank name saving and related "Bobby bug"

Account-related .sav files usually contained a name structure, such as "*Name-ID.sav*". However, they were sometimes inexplicably saved without any name or ID specification, e.g., ".sav" (i.e., blank name saving). This bug messed with any code trying to save, load or delete the existing account-related *Name-ID.sav* files and sometimes even replaced the existing *Name-ID.sav* file on the tablet, causing loss of data. Moreover, this blank name saving bug

sometimes resulted in the occurrence of another bug, i.e., the so-called “Bobby bug”. More specifically, when the code failed to load an account-related profile, the name “Bobby” appeared on the main menu screen, instead of the name of the participant’s account, as it was used as a place holder name to test the user interface of the game in the developmental phase. Moreover, in this case, the story game only played the first sub-phase of the first game phase over and over again. Occurrence of these bugs in the current intervention study resulted in a number of home visits where members of the research group fully reset the entire game and existing profiles. Yet, thanks to the existence of the AutoSyncer application, this bug only caused a limited amount of data loss (i.e., in seven participants). However, when applying the game in future studies, fixing these bugs must take the highest priority.
