# Supplementary material for: Feasibility, Enjoyment, and Language Comprehension Impact of a Tablet- and GameFlow-Based Story-Listening Game for Kindergarteners: Methodological and Mixed Methods Study
Source: JMIR Serious Games. 2022 Mar 23;10(1):e34698. doi: 10.2196/34698 (PMC8987971; doi:10.2196/34698)
Supplement: Multimedia Appendix 4 [file games_v10i1e34698_app4.pdf]

Multimedia Appendix 4: Overview of the child and parental categorical questions and response possibilities

Question		Response categories
**Parental questionnaire**		
	How motivated was your child when he/she had to play the story game?	Not motivated/Little motivated/No opinion/Relatively motivated/Very motivated
	Did your child need encouragement to play the story game?	My child always needed encouragement/My child mostly needed encouragement/My child only sometimes needed encouragement/My child never needed encouragement
	Was your child able to play the story game with sustained attention?	Yes/No
**Child questionnaire**		
	How much did you like the story game?	I did not like it at all/I did not like it/ I liked it/I liked it a lot/ I liked it very much
	Would you be willing to play the story game again?	Yes/No
